# Progesterone for Neurodevelopment in Fetuses With Congenital Heart Defects: A Randomized Clinical Trial

**DOI:** 10.1001/jamanetworkopen.2024.12291

**Published:** 2024-05-28

**Authors:** J. William Gaynor, Julie S. Moldenhauer, Erin E. Zullo, Nancy B. Burnham, Marsha Gerdes, Judy C. Bernbaum, Jo Ann D’Agostino, Rebecca L. Linn, Brenna Klepczynski, Isabel Randazzo, Gabrielle Gionet, Grace H. Choi, Antoneta Karaj, William W. Russell, Elaine H. Zackai, Mark P. Johnson, Juliana S. Gebb, Shelly Soni, Suzanne E. DeBari, Anita L. Szwast, Rebecca C. Ahrens-Nicklas, Theodore G. Drivas, Marin Jacobwitz, Daniel J. Licht, Arastoo Vossough, Susan C. Nicolson, Thomas L. Spray, Jack Rychik, Mary E. Putt

**Affiliations:** 1 Division of Cardiothoracic Surgery, Department of Surgery, Children’s Hospital of Philadelphia, and the Perelman School of Medicine, University of Pennsylvania, Philadelphia; 2 Center for Fetal Diagnosis and Treatment, Children’s Hospital of Philadelphia and the Perelman School of Medicine, University of Pennsylvania, Philadelphia; 3 Department of Psychology, Children’s Hospital of Philadelphia, and the Perelman School of Medicine, University of Pennsylvania, Philadelphia; 4 Department of Pediatrics, Children’s Hospital of Philadelphia, and the Perelman School of Medicine, University of Pennsylvania, Philadelphia; 5 Division of Anatomic Pathology, Children’s Hospital of Philadelphia, and the Perelman School of Medicine, University of Pennsylvania, Philadelphia; 6 Department of Biostatistics, Epidemiology, and Informatics, the Perelman School of Medicine, University of Pennsylvania, Philadelphia; 7 Division of Genetics, Department of Pediatrics, Children’s Hospital of Philadelphia, and the Perelman School of Medicine, University of Pennsylvania, Philadelphia; 8 Division of Cardiology, Department of Pediatrics, Children’s Hospital of Philadelphia and the Perelman School of Medicine, University of Pennsylvania, Philadelphia, Pennsylvania; 9 Division of Translational Medicine and Human Genetics, Department of Medicine, Perelman School of Medicine, University of Pennsylvania, Philadelphia; 10 Division of Neurology, Department of Pediatrics, Children’s Hospital of Philadelphia, and the Perelman School of Medicine, University of Pennsylvania, Philadelphia; 11 Division of Radiology, Children’s Hospital of Philadelphia, and the Perelman School of Medicine, University of Pennsylvania, Philadelphia; 12 Division of Cardiac Anesthesia, Department of Anesthesia and Critical Medicine, Children’s Hospital of Philadelphia, and the Perelman School of Medicine, University of Pennsylvania, Philadelphia

## Abstract

**Importance:**

Neurodevelopmental outcomes for children with congenital heart defects (CHD) have improved minimally over the past 20 years.

**Objectives:**

To assess the feasibility and tolerability of maternal progesterone therapy as well as the magnitude of the effect on neurodevelopment for fetuses with CHD.

**Design, Setting, and Participants:**

This double-blinded individually randomized parallel-group clinical trial of vaginal natural progesterone therapy vs placebo in participants carrying fetuses with CHD was conducted between July 2014 and November 2021 at a quaternary care children’s hospital. Participants included maternal-fetal dyads where the fetus had CHD identified before 28 weeks’ gestational age and was likely to need surgery with cardiopulmonary bypass in the neonatal period. Exclusion criteria included a major genetic or extracardiac anomaly other than 22q11 deletion syndrome and known contraindication to progesterone. Statistical analysis was performed June 2022 to April 2024.

**Intervention:**

Participants were 1:1 block-randomized to vaginal progesterone or placebo by diagnosis: hypoplastic left heart syndrome (HLHS), transposition of the great arteries (TGA), and other CHD diagnoses. Treatment was administered twice daily between 28 and up to 39 weeks’ gestational age.

**Main Outcomes and Measures:**

The primary outcome was the motor score of the Bayley Scales of Infant and Toddler Development-III; secondary outcomes included language and cognitive scales. Exploratory prespecified subgroups included cardiac diagnosis, fetal sex, genetic profile, and maternal fetal environment.

**Results:**

The 102 enrolled fetuses primarily had HLHS (n = 52 [50.9%]) and TGA (n = 38 [37.3%]), were more frequently male (n = 67 [65.7%]), and without genetic anomalies (n = 61 [59.8%]). The mean motor score differed by 2.5 units (90% CI, −1.9 to 6.9 units; *P* = .34) for progesterone compared with placebo, a value not statistically different from 0. Exploratory subgroup analyses suggested treatment heterogeneity for the motor score for cardiac diagnosis (*P *for interaction = .03) and fetal sex (*P *for interaction = .04), but not genetic profile (*P *for interaction = .16) or maternal-fetal environment (*P *for interaction = .70).

**Conclusions and Relevance:**

In this randomized clinical trial of maternal progesterone therapy, the overall effect was not statistically different from 0. Subgroup analyses suggest heterogeneity of the response to progesterone among CHD diagnosis and fetal sex.

**Trial Registration:**

ClinicalTrials.gov Identifier: NCT02133573

## Key Points

**Question:**

Does maternal progesterone therapy in fetuses with congenital heart defects (CHD) improve neurodevelopmental outcomes?

**Findings:**

In this randomized clinical trial of 102 mother-fetal dyads receiving maternal progesterone therapy, the treatment benefit of progesterone in the overall population was small and not statistically different from 0. Subgroup analyses suggested heterogeneity of treatment effect in Motor Scores by fetal sex and among fetuses with different types of CHD.

**Meaning:**

These results suggest that continued research into the possible benefits of progesterone for neurodevelopmental outcomes for a subset of patients with CHD appears warranted.

## Teaser

This randomized clinical trial investigates the magnitude of the effect of maternal progesterone therapy on neurodevelopment for fetuses with congenital heart defects (CHDs).

## Introduction

Despite improved survival after surgical repair, individuals with congenital heart defects (CHD) commonly display substantial neurodevelopmental problems.^1^ There has been minimal progress in improving neurodevelopmental outcomes for these children.^2,3^ Brain dysmaturity at birth, characterized by microcephaly, delayed maturation of white matter, and simplified cortical folding, is common. Brain dysmaturity is a primary mechanism underlying perioperative white matter brain injury, an important contributor to adverse neurodevelopmental outcomes.^4,5,6,7,8^

Sex steroid hormones, including progesterone, are critical to brain development, including the white matter.^9^ Progesterone and its metabolites promote the viability and regeneration of neurons and appear to enhance myelination and maturation of immature progenitor cells to mature oligodendrocytes, which are more resistant to hypoxic and/or ischemic injury.^9^ In addition, progesterone appears neuroprotective in several experimental models, including traumatic brain injury.^10^

In the OPPTIMUM study of women at increased risk for preterm birth, vaginal progesterone did not yield a significantly decreased risk of the composite outcome (preterm birth, adverse neonatal outcomes, or cognitive scores).^11,12^ However, the progesterone-treated group had fewer neonatal deaths and reduced brain injury. Recent meta-analyses indicate that progesterone therapy reduces the risk of preterm birth and some neonatal morbidities.^13,14^ However, these trials typically excluded pregnancies with a structural fetal anomaly.^11,14^ This single-center phase 2 study aimed to assess benefits of prenatal progesterone therapy on neurodevelopmental outcomes for fetuses with CHD and inform the design of subsequent multicenter trials.

## Methods

**Figure 1.**
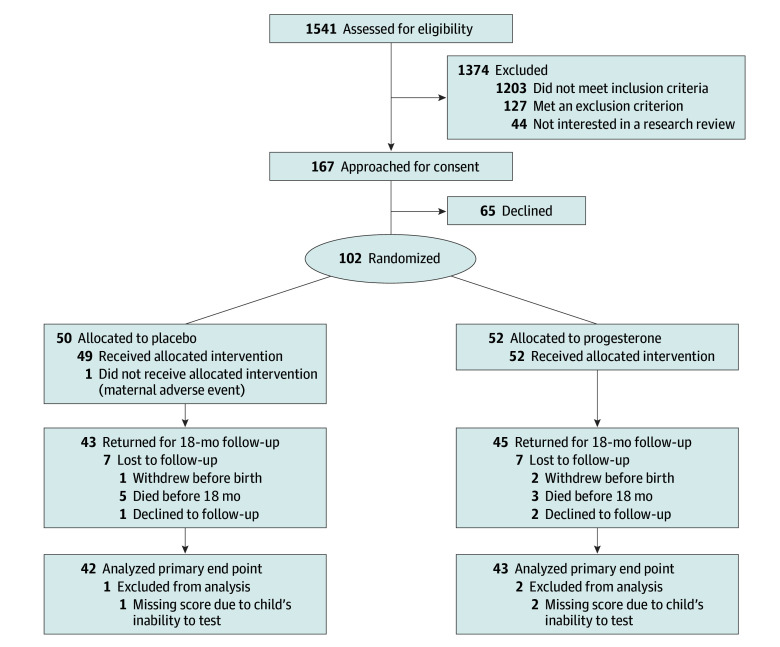
Consolidated Standards of Reporting Trials (CONSORT) Diagram

A placebo-controlled double-blinded individually randomized parallel-group phase 2 clinical trial of vaginal natural progesterone therapy vs placebo was conducted in participants carrying fetuses with CHD at a single center (Children’s Hospital of Philadelphia [CHOP]). The inclusion criteria were a maternal-fetal dyad with CHD identified before 28 weeks’ gestational age (GA) likely to need surgery with cardiopulmonary bypass prior to 44 weeks’ corrected GA. Exclusion criteria included a major genetic or extracardiac anomaly other than 22q11 deletion syndrome and known contraindication to progesterone (eMethods in Supplement 1). The CHOP institutional review board approved the study. A data safety monitoring board (DSMB) reviewed the trial at approximately 6-month intervals between May 2014 and May 2022. Written informed consent, including consent to publish, was obtained from both parents or guardians. This study followed the Consolidated Standards of Reporting Trials (CONSORT) reporting guideline (Figure 1).

Participants were stratified by fetal CHD diagnosis (hypoplastic left heart syndrome [HLHS], transposition of the great arteries [TGA], and other CHD diagnoses), and then 1:1 block-randomized (block size = 4). Treatment was initiated by 28 weeks’ GA. Mothers were to administer the study drug vaginally twice daily up to 39 weeks’ GA and record the date and time of each dose and any adverse effects in a diary.

At 18 months of age, participants underwent neurodevelopmental and genetic evaluations. The primary outcome was the motor scale of the Bayley Scales of Infant and Toddler Development-III (BSID-III).^15^ Secondary outcomes included language and cognitive scales, as well as fine and gross motor scores and receptive and expressive communication scores. Descriptions of reimbursement, randomization, drug administration, postnatal management, adverse event (AE) monitoring, and genetic testing, including apolipoprotein E (*APOE*) genotype, are provided in the eMethods in Supplement 1.

### Statistical Methods

The primary intention-to-treat analysis compared mean differences between the progesterone and placebo groups using CIs and *t* tests. In this phase 2 trial, we prespecified a 90% CI level (90%) and corresponding type I error rates of .10.^16,17^ Sensitivity analyses included (1) multiple imputation using full conditional specification to address incomplete data and (2) linear models with adjustment for baseline covariates. Per-protocol analyses using participants with 80% or 90% adherence were adjusted for baseline covariates.

Six subgroups (cardiac diagnosis, fetal sex, genetic profile, impaired maternal-fetal environment [MFE], carrier of 22q deletion, and neonatal surgery) were prespecified. Following published guidelines, an *F* test was used to compare a linear model including an interaction term between treatment and each subgroup with the reference model including only a main effect of treatment.^18^ Models were adjusted for all subgroup variables, other than the term involving the interaction. CIs for the subgroup treatment effect based on the interaction model were visualized using forest plots.^18,19^ For this exploratory analysis, *P* values were unadjusted for multiple comparisons and instead informally compared with the number of tests expected below a threshold of .10 by chance.

The target sample size for this phase 2 trial ranged from 110 to 130 participants anticipated to enroll with a goal of 80 evaluated at 18 months. Attrition was expected from death or other loss to follow-up. The primary goal was to estimate the mean difference between groups in the motor score. With 40 evaluable participants per treatment group, and an anticipated SD of 15, a half width of 5.6 for a 2-sided 90% CI on the mean difference was expected. No interim analyses were prespecified.

Variables related to study conduct and adherence, as well as outcomes related to the babies’ birth, were summarized and CIs constructed using linear or generalized linear models with cardiac diagnosis as a covariate (eMethods in Supplement 1) Post hoc, reasons for drug termination at less than 39 weeks’ GA were tabulated. To be consistent, the 2-sided type I error rate and CI levels are .10 and .90 throughout. We avoid the term statistical significance as this often implies *P* < .05. Analyses were carried out in R version 4.2.1 (R Project for Statistical Computing) from June 2022 to April 2024. The trial protocol and statistical analysis plan are included in Supplement 2.

## Results

Among the 102 enrolled fetuses, 52 (50.9%) had HLHS and 38 (37.3%) had TGA; 67 (65.6%) were male and 35 (34.3%) were female; 61 (59.8%) were without genetic anomalies. Among 102 pregnant participants, 7 (6.9%) were Asian, 9 (8.8%) were Black, 8 (7.8%) were Hispanic, 77 (75.5%) were White; and median (IQR) age was 31.0 (27.2-33.8) years. Participants (52 for progesterone group, 50 for placebo group) were enrolled between July 2014 and February 2020 (Figure 1). In February 2020, the DSMB recommended terminating the study based on an unblinded interim analysis concluding that (1) at least 80 participants, the target sample size, would complete the 18-month assessment and (2) additional participants would be unlikely to alter the determination of any treatment effect. At termination, 4 pregnant participants (3 progesterone and 1 placebo) were discontinued from treatment. Their infants were included in the intention-to-treat analysis.

Table 1 indicates similar baseline characteristics among treatment groups. Baseline characteristics by cardiac diagnosis, cardiac class, and genetic anomalies appear in eTables 1 to 3 in Supplement 1.^20^ Table 2 indicates characteristics of participants during the intervention and postnatal periods were also similar by treatment group. Between 74% (placebo) and 75% (progesterone) of participants had at least 80% adherence to the study drug. The drug was terminated before 39 weeks’ GA in 50% of progesterone (26 of 52) vs 66% of placebo (33 of 50) users (OR, 0.4 [90% CI, 0.2-0.8]). eTable 4 in Supplement 1 provides reasons for drug termination. eTable 5 in Supplement 1 describes the delivery and neonate. Induction of labor was more common in the progesterone group (odds ratio [OR], 2.3 [90% CI, 1.2-4.6]; *P* = .04]). The mean GA at delivery did not differ by group (mean [SE] of 0.3 [0.3] weeks later for progesterone vs placebo [*P* = .35]). In post hoc analyses, when categorized into early preterm (<32 weeks), preterm (32-36 weeks 6 days), early term (37-38 weeks 6 days) and full term (>39 weeks), babies of mothers using progesterone vs placebo had 0.6-fold lower cumulative odds (90% CI, 0.3-1.1; *P* = .13) of being preterm or early term compared with full term, with 57.7% of progesterone vs 44.0% of placebo babies born full term. Among 99 babies born during the study, 96 underwent a cardiac operation as neonates at a median (IQR) age of 4 (2-5) days; 1 died prior to surgery, and 2 underwent first surgery at 90 and 115 days of age. eTable 6 in Supplement 1 shows similar operative management across treatment groups.

**Table 1.** Baseline Characteristics of Maternal and Fetal Participants

Variables	No. (%)		
	Progesterone (n = 52)	Placebo (n = 50)	Overall (n = 102)
Maternal participants			
Age, median (IQR), y	31.0 (26.0-34.2)	31.0 (28.2-33.0)	31.0 (27.2-33.8)
Race			
American Indian or Alaska Native	2 (3.8)	0	2 (2.0)
Asian	4 (7.7)	3 (6.0)	7 (6.9)
Black	6 (11.5)	3 (6.0)	9 (8.8)
Multiracial	4 (7.7)	1 (2.0)	5 (4.9)
White	36 (69.2)	41 (82.0)	77 (75.5)
Unknown	0	2 (4.0)	2 (2.0)
Hispanic ethnicity			
Yes	3 (5.8)	5 (10.0)	8 (7.8)
No	48 (92.3)	43 (86.0)	91 (89.2)
Unknown	1 (1.9)	2 (4.0)	3 (2.9)
Education			
High school	7 (13.5)	3 (6.0)	10 (9.8)
College	23 (44.2)	33 (66.0)	56 (54.9)
Post graduate degree	22 (42.3)	14 (28.0)	36 (35.3)
Income			
<$50 000	7 (13.5)	4 (8.0)	11 (10.8)
$50 000-$100 000	14 (26.9)	16 (32.0)	30 (29.4)
>$100 000	19 (36.5)	25 (50.0)	44 (43.1)
Unknown	12 (23.1)	5 (10.0)	17 (16.7)
Maternal-fetal environment^a^			
Impaired	19 (36.5)	17 (34.0)	36 (35.3)
Unimpaired	33 (63.5)	33 (66.0)	66 (64.7)
Fetal participants			
Diagnosis^b^			
HLHS	27 (51.9)	25 (50.0)	52 (51.0)
TGA	19 (36.5)	19 (38.0)	38 (37.3)
Other	6 (11.5)	6 (12.0)	12 (11.8)
Sex			
Male	36 (69.2)	31 (62.0)	67 (65.7)
Female	16 (30.8)	19 (38.0)	35 (34.3)
Genetic classification^b^			
Normal	29 (55.8)	32 (64.0)	61 (59.8)
Suspect	12 (23.1)	5 (10.0)	17 (16.7)
Abnormal	9 (17.3)	11 (22.0)	20 (19.6)
Unknown	2 (3.8)	2 (4.0)	4 (3.9)
22q11.2 deletion^b^			
Yes	4 (7.7)	1 (2.0)	5 (4.9)
No	46 (88.5)	47 (94.0)	93 (91.2)
Unknown	2 (3.8)	2 (4.0)	4 (3.9)
*APOE* genotype^c^^,^^d^			
ε2	8 (15.4)	6 (12.0)	14 (13.7)
ε2/ε4	0 (0)	1 (2.0)	1 (1.0)
ε3	26 (50.0)	30 (60.0)	56 (54.9)
ε4	14 (26.9)	10 (20.0)	24 (23.5)
Missing	4 (7.7)	3 (6.0)	7 (6.9)

Abbreviations: *APOE*, apolipoprotein E; HLHS, hypoplastic left heart syndrome; TGA, transposition of the great arteries.

^a^ Impaired maternal-fetal environment indicates a mother with 1 or more of gestational hypertension, gestational diabetes, tobacco use, or hypothyroidism at baseline or preeclampsia during the pregnancy.

^b^ Thirty-three participants were cardiac class I (32 with TGA and 1 with other congenital heart defect); 13 participants were cardiac class II (10 with other, 2 with TGA, and 1 with HLHS); 2 participants were class III (1 each of HLHS and TGA); 50 participants were cardiac class IV (49 with HLHS, 1 with other) (eTable 2 in Supplement 1).

^c^ Genetic classification, 22q11.2 deletion, and *APOE* genotype are often not known until after birth. Three individuals withdrew before birth, contributing to the missing data.

^d^ ε2 indicates homozygous for ε2 or heterozygous ε2/ε3. ε3 indicates homozygous for ε3. ε4 indicates homozygous for ε4 or heterozygous ε4/ε3.

**Table 2.** Description of the Intervention and Postnatal Phases

Variables	Progesterone (n = 52)^a^	Placebo (n = 50)^a^	Treatment effect (90% CI)^b^
			Difference
Gestational age drug initiated, median (IQR), wk	27.9 (27.5 to 28.3)	27.9 (27.4 to 28.1)	<0.1 (−0.2 to 0.2)
Drug use duration, median (IQR), d	75.5 (65.5 to 78.0)	75.0 (69.8 to 77.2)	−3.9 (−11.5 to 3.7)
No. of doses taken, median (IQR)	155.0 (146.8 to 163.2)	151.5 (142.5 to 158.8)	6.0 (−2.0 to 14.0)
			**OR**
Adherence, %^c^^,^^d^			
>90	33 (63.5)	28 (56.0)	1 [Reference]
81-90	6 (11.5)	9 (18.0)	0.5 (0.2 to 1.4)
51-80	5 (9.6)	6 (12.0)	0.7 (0.2 to 2.0)
<50^d^	8 (15.4)	5 (10.0)	1.3 (0.2 to 3.8)
Drug terminated			
≥39 wk	26 (50.0)	15 (30.0)	1 [Reference]
<39 wk	26 (50.0)	35 (70.0)	0.4 (0.2 to 0.8)
Cardiac operation during first admission			
Yes	49 (94.2)	47 (94.0)	1 [Reference]
No	1 (1.9)	2 (4.0)	0.5 (<0.1 to 4.0)
Age at surgery, median (IQR), d	4.0 (2.0 to 5.0)	4.0 (3.0 to 5.0)	−0.3 (−5.4 to 4.9)
Returned for 18-mo follow-up			
Yes	45 (86.5)	43 (86.0)	1 [Reference]
No (deceased)	3 (5.8)	5 (10.0)	0.5 (0.1 to 1.9)
No (declined)	2 (3.8)	1 (2.0)	1.8 (0.2 to 14.0)

Abbreviation: OR, odds ratio.

^a^ Three participants (1 with transposition of the great arteries and 2 with hypoplastic left heart syndrome) had missing data for some variables. Percentages are based on the total number of participants assigned to the treatment group and do not add to 100% when there are missing data.

^b^ Values are model-based mean (90% CI) differences between progesterone and placebo for continuous variables and odds ratios for categorical variables. Each model included cardiac diagnosis as a stratification variable. Linear regression models were used for continuous variables, logistic or multinomial regression for categorical variables. ORs are relative to the reference category.

^c^ Adherence is the number of doses taken relative to the number of intended doses for the duration of the pregnancy. See description in eMethods in Supplement 1.

^d^ The less than 50% category includes 3 out of the 4 participants whose study drug was terminated when the data and safety monitoring board recommended termination of treatment.

A total of 88 out of 102 enrolled participants (86% [90% CI, 79%-92%]) returned for the 18-month evaluation. Three participants withdrew prior to birth, 8 infants died, and 3 did not return for follow-up. Additionally, 3 participants (1 placebo and 2 progesterone) who returned were unable to complete all BSID-III testing. Table 3 shows the mean (SD) composite and component scores by treatment group and the mean treatment difference, either pooled or stratified by diagnosis. Pooled across diagnoses, the mean difference in motor score between progesterone and placebo was 2.5 units (90% CI, −1.9 to 6.9 units; *P* = .34) with similar mean differences of 1.6 units for language (90% CI, −4.5 to 7.8 units; *P* = .64) and 1.2 units for cognitive scores (90% CI, −3.0 to 5.4 units; *P* = .66); none differed statistically from 0. Sensitivity analyses including stratification by diagnosis (eTable 7 in Supplement 1), adjustment for baseline covariates including cardiac diagnosis, fetal sex, presence of genetic anomalies, impaired MFE, and income, and using multiple imputation to address missing outcome data (eTables 8 and 9 in Supplement 1) yielded similar results. A per-protocol analysis yielded similar results (eTable 10 in Supplement 1).

**Table 3.** Analyses of Neurodevelopment Outcomes at 18 Months for Primary and Secondary End Points Using Pooled Data or Stratified by Cardiac Diagnosis

BSID-III score	Group means, mean (SD)^a^		Treatment effect			
	Progesterone	Placebo	Pooled^b^		Stratified^c^	
			Mean difference (90% CI)	*P* value	Mean difference (90% CI)	*P* value
Composite scores						
Motor	90.0 (11.5)	87.5 (12.7)	2.5 (−1.9 to 6.9)	.34	2.7 (−1.6 to 7.1)	.30
Language	86.1 (12.2)	84.5 (11.4)	1.6 (−4.5 to 7.8)	.66	1.7 (−4.4 to 7.9)	.64
Cognitive	92.4 (15.1)	91.2 (18.9)	1.2 (−3.0 to 5.4)	.64	1.5 (−2.7 to 5.7)	.56
Component scores						
Motor						
Fine	9.4 (2.1)	8.8 (2.5)	0.6 (−0.3 to 1.5)	.29	0.6 (−0.3 to 1.5)	.27
Gross	7.6 (2.7)	7.0 (2.3)	0.6 (−0.3 to 1.4)	.26	0.6 (−0.2 to 1.4)	.21
Language						
Expressive	7.5 (2.6)	7.5 (3.4)	<−0.1 (−1.1 to 1.1)	.97	<−0.1 (−1.1 to 1.1)	.98
Receptive	7.7 (3.0)	7.1 (3.3)	0.61 (−0.5 to 1.8)	.38	0.6 (−0.5 to 1.8)	.36

Abbreviation: BSID-III, Bayley Scales of Infant and Toddler Development-III.

^a^ Across hypoplastic left heart syndrome, other congenital heart defects, and transposition of the great arteries.

^b^ Data pooled across diagnoses and analyzed using a *t* test.

^c^ Using a linear model stratified by cardiac diagnosis with a Wald test.

### Exploratory Subgroup Analysis

**Figure 2.**
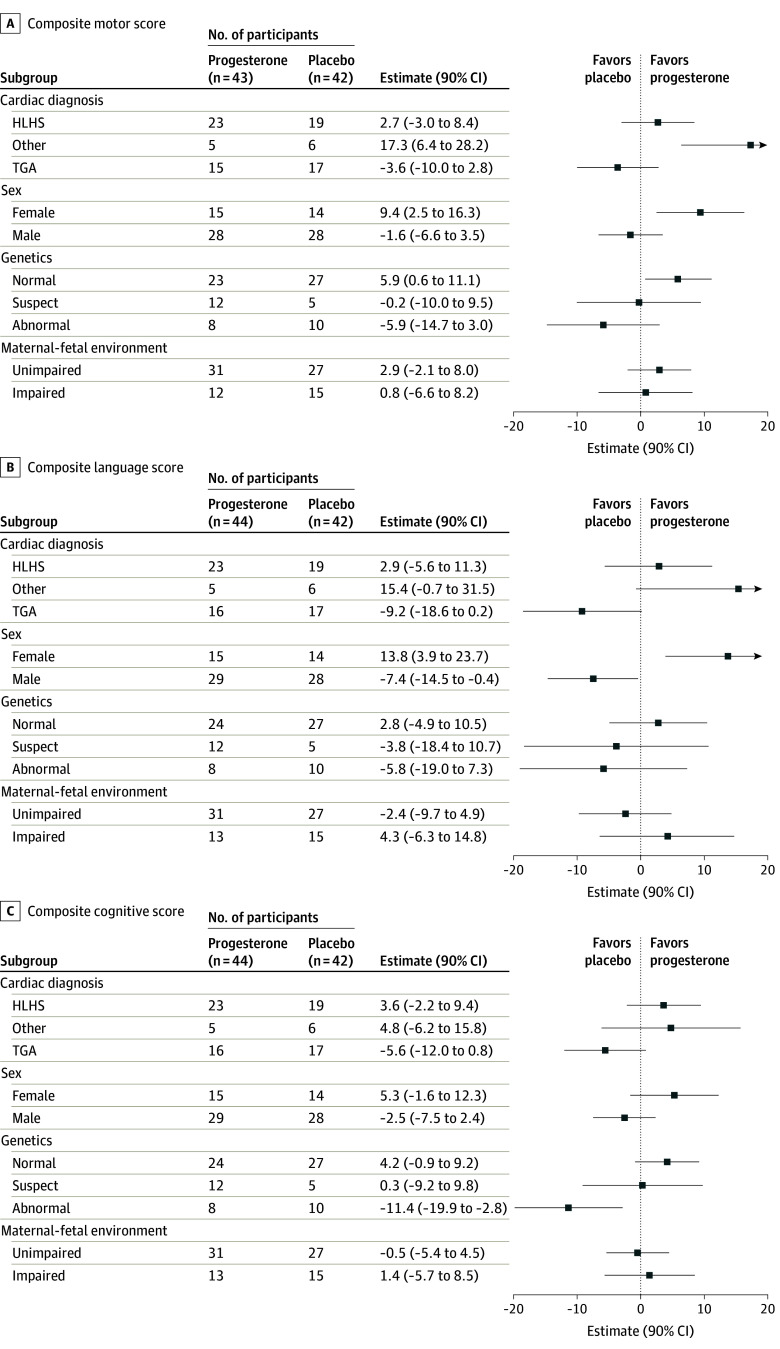
Forest Plots for Bayley Scales of Infant and Toddler Development-III Composite Scores by Prespecified Subgroups Exploratory subgroup analyses showing forest plots of mean difference (90% CI) for each composite score from the model including an interaction between the treatment effect and each of 4 prespecified subgroups (cardiac diagnosis, fetal sex, genetic profile, and maternal-fetal environment). The *P* values for the global test of an interaction for motor score were: *P* = .03 (diagnosis), *P* = .04 (sex), *P* = .16 (genetic profile), and *P* = .70 (maternal-fetal environment); for language score were: *P* = .03 (diagnosis), *P* = .005 (sex), *P* = .59 (genetic profile), and *P* = .39 (maternal-fetal environment); and for cognitive score were: *P* = .160 (diagnosis), *P* = .13 (sex), *P* = .04 (genetic profile), and *P* = .27 (maternal-fetal environment). Two prespecified subgroups, whether the infant underwent neonatal cardiac surgery or not, and whether the infant had 22q11 syndrome or not, were not analyzed due to small numbers of infants in 1 level of the subgroup. HLHS indicates hypoplastic left heart syndrome; TGA, transposition of the great arteries.

A global *F* test of the interaction between treatment and each subgroup (cardiac diagnosis, fetal sex, presence of genetic anomalies, and MFE) was used to identify subgroups of interest. Due to insufficient number of participants, the 22q deletion and the neonatal surgery subgroup analyses were not attempted. For the motor score, the global *F* test suggested possible heterogeneity of treatment effects for cardiac diagnosis (*P* for interaction = .03) and fetal sex (*P* for interaction = .04), but not genetic profile (*P* for interaction = .16) or MFE (*P* for interaction = .70); 2 out of 4 subgroups (50%) had *P* for interaction < .10. In the absence of heterogeneity for any subgroup, *P* for interaction < .10 is expected in 10% of these *F* tests. Larger proportions of *P* for interaction < .10 suggest treatment heterogeneity with the caveat that these exploratory results are intended to generate hypotheses for future study. Figure 2A suggests the other CHD diagnostic group and female neonates benefited from progesterone. For the motor score, mean scores for progesterone vs placebo were positive for HLHS participants and negative for TGA with a 90% CI that covered 0.

For the language score, cardiac diagnosis (*P* for interaction = .03) and fetal sex (*P* for interaction = .005), but not genetic profile (*P* for interaction = .59) or MFE (*P* for interaction = .39) had *P* for interaction < .10. On average, language scores for female children in the progesterone group were higher than placebo; the opposite was true for male children. Similar to the motor score, mean scores for progesterone vs placebo were positive for participants with HLHS and other CHD and negative for TGA with 90% CI that covered 0. For cognitive score, 1 out of 4 tests (genetic profile, *P* for interaction = .04) had *P* for interaction < .10. Those with abnormal genetic profiles had worse cognitive scores in the progesterone group compared with the placebo group. Across all 12 tests of an interaction (3 Bayley scores and 4 subgroups), 5 had *P* for interaction < .10 compared with an expected value of 1.2 by chance.

### Adverse Events

Rates of maternal and infant adverse events were similar by study group (eTables 11 and 12 and eFigures 2 and 3 in Supplement 1). Most deaths occurred before 6 months (eFigure 1 in Supplement 1). Of the 8 who died on study, 7 had HLHS and 1 had TGA. For HLHS, the 18-month mortality rate was 7.4% (90% CI, 1.3%-21.5%) for progesterone vs 20.0% (90% CI, 8.2%-37.5%) for placebo. Among the HLHS participants, those who died were born at a median (IQR) GA of 36.7 (33.1-37.8) weeks compared with 39.0 (38.4-39.3) weeks among those alive at 18 months (post hoc *P* = .001). eTable 13 in Supplement 1 includes more details.

## Discussion

To our knowledge, this is the first randomized clinical trial of fetal neuroprotection in the CHD population. Vaginal progesterone was well tolerated with few side effects in mothers carrying a fetus with CHD. When pooled across diagnoses, the mean difference in motor score between progesterone and placebo was not statistically different from 0. Differences between groups in language and cognitive scores showed a similar pattern of not being statistically different from 0. However, in the CHD population, with an increased prevalence of developmental disabilities, even relatively small improvements in mean outcomes could substantially reduce the number of severely impaired individuals.^21^

Exploratory subgroup analyses suggested substantial heterogeneity in treatment effect, specifically for the motor and language scores among cardiac diagnosis and fetal sex subgroups. Notably, motor scores for the other CDH diagnosis and female groups were substantially better for the progesterone vs placebo groups; language scores were also substantially better for females. Among the HLHS group, the small treatment effect benefit of progesterone for motor and language scores did not differ statistically from 0. Lastly, mean motor and language scores among the TGA group receiving progesterone showed no benefit vs placebo. Among male individuals, cognitive scores did not benefit from progesterone treatment and indeed mean language scores were worse.

Heterogeneity of treatment effect occurs when the response to an intervention differs systematically across individuals with different characteristics.^22,23,24,25^ Intrinsic biological characteristics of a participant (eg, diagnosis, fetal sex, genetics) or extrinsic factors (eg, socioeconomic factors) may systematically alter response to a therapy.^22,23,24,25^ Qualitative heterogeneity, where the direction of response varies within subgroups, complicates interpretation of an overall effect because the results for the component subgroups tend to cancel when pooled across participants. Our study suggests qualitative heterogeneity of treatment effect based on cardiac diagnosis and fetal sex; thus, the overall treatment effect may overestimate or underestimate the benefit or harm in some patients.

In this phase 2 trial, the subgroup analyses were aimed at hypothesis generation for a possible future trial. Subgroups were prespecified based on known and/or anticipated risk factors for poor neurodevelopmental outcomes in this population; the analysis followed recommended guidelines.^18,26,27,28^ Notably, cardiac diagnosis and fetal sex are known at baseline and could be used as exclusion and/or inclusion criteria for a future trial. For the motor and language scores, the observation that 2 of 4 (50%) of each subgroup analyses yielded *P* < .10 supports continued research into the benefit of progesterone, using a more restricted population.

Our results are consistent with increased recognition of the importance of sex as a biological variable and its effects on disease phenotypes and the response to treatment.^29,30,31^ The National Institutes of Health notes: “Failure to account for sex as a biological variable may undermine the rigor, transparency and generalizability of research findings.”^31^ Fetal sex moderates placental structure and function, in utero development, and outcomes of pregnancy.^32,33^ Developmental outcomes are often more impaired for males compared with females exposed to an adverse in utero environment.^32^ Maternal complications, except preeclampsia, are more common when the fetus is male.^34^ Multiple molecular and cellular processes in the placenta and developing fetus may lead to sex-based risk factors that manifest in context-specific ways (eg, the fetus with CHD).^33^

Previous studies of CHD have demonstrated sexual dimorphism in neurodevelopmental outcomes in infants after cardiac surgery and in children in response to environmental exposures.^35,36^ For example, across diagnoses, male neonates with CHD are more likely to have white matter injury prior to surgery.^8^ In contrast, the risk for pregnancy loss with fetal CHD is increased when the fetus is female.^37^ We did not find reports separated by fetal sex describing outcomes of progesterone for preterm birth in humans. However experimental models of brain injury suggest sexual dimorphism in the response to progesterone therapy with some showing benefit to males and others to females.^38,39,40^ In an experimental model, trophoblast-specific deletion of the gene-encoding synthetic enzyme for allopregnanolone demonstrated that placental allopregnanolone insufficiency in male animals was associated with cerebellar white matter anomalies and changes in social and motor behaviors.^41^ Treatment with allopregnanolone remediated these changes, suggesting that placental hormone replacement could benefit subsequent neurodevelopmental outcomes.^41^ In the same model, placental allopregnanolone insufficiency in female animals was associated with abnormal cortical development and somatosensory behavioral deficits.^42^

Heterogeneity of treatment effect among different types of CHD has been identified in previous randomized trials.^43,44^ In a randomized clinical trial, Clancy and colleagues^43^ found that allopurinol provided neuroprotection in newborns with HLHS undergoing cardiac surgery. Newborns without HLHS did not benefit from allopurinol.^43^

Placental insufficiency is a risk factor for brain dysmaturity and poor neurodevelopmental outcomes when the fetus has CHD.^45^ Placental structure and function vary by type of CHD; for example, placentas are abnormal more often in those with aortic obstruction vs those without (83% vs 42%).^46^ In a transcriptome analysis, placentas from individuals with TGA demonstrated upregulation of multiple genes compared to HLHS participants, with reduced cell activity and nutrient transport capability in individuals with HLHS.^47^ Considering the current findings, we hypothesize that differentially regulated compensatory mechanisms in the placenta could contribute to differences in outcomes between cardiac diagnoses.^47^ In our study, the HLHS and other CHD subgroups, but not TGA, were characterized by arch obstruction, and by implication were more likely to have abnormal placentas. We collected placental tissue from the participants; these results will be reported elsewhere.

While our study was not designed to address mortality, and the 18-month survival for the cohort was excellent, the on-study mortality rate for the HLHS participants on progesterone (7.4%) was noticeably better than for the placebo group (20%). The trend toward increased full-term birth in the progesterone group suggests a potential biological mechanism. All HLHS participants who died in the placebo group were born before 39 weeks’ GA, and intriguingly, 1 of the participants who died in the progesterone group, born at 36.9 weeks’ GA, had very poor adherence. Similarly, in the OPPTIMUM study neonatal death rates were lower in the progesterone group.^12^

### Limitations

This study has limitations. Our single-center randomized phase 2 trial was designed to evaluate the potential activity of progesterone for neuroprotection of fetuses with CHD, and to assess the value of a larger multicenter phase 3 trial for this agent. The sample size was intentionally limited and the CIs and the type I error rate were a priori set to 90% and .10 respectively.^17,48,49,50^ We balanced the possibility of higher false-positive rates than studies using a type I error rate of 0.05 with the need to restrict the sample size.^16,17^ With the small sample size and despite the randomization; the chance that differences between groups reflect imbalance in baseline characteristics is increased, particularly for subgroup analyses.^48^ The study population is largely non-Hispanic White with HLHS or TGA, which may limit generalizability. Additionally, the neurodevelopment assessment was performed early in life and may not predict later outcomes.

## Conclusions

In this randomized clinical trial of progesterone therapy in participants carrying fetuses with CHD, the effect of progesterone in the combined population was not statistically different from 0. Exploratory analyses suggest heterogeneity of treatment effect among types of CHD and by sex. We recommend continued study into possible benefits of progesterone for a targeted subset of patients with CHD.

## Notes

Supplement 1.
eMethods.

eReferences
**eTable 1.** Baseline Characteristics of Mothers and Fetuses Stratified by Cardiac Diagnosis **eTable 2.** Cardiac Class and Diagnosis **eTable 3.** Genetic Evaluation **eTable 4.** Reasons for Drug Termination Before 39 Weeks GA **eTable 5.** Delivery and Neonate Characteristics **eTable 6.** Operative Characteristics **eTable 7.** BSID-III Scales Overall and by Pre-specified Subgroups **eTable 8.** Treatment Effect Estimates for BSID-III Scales Using Linear Model Adjusted for Pre-specified Covariates **eTable 9.** Treatment Effect Estimates for BSID-III Scales Using Multiple Imputation to Address Incomplete Outcome Data **eTable 10.** Per-Protocol Analysis **eTable 11.** Adverse Events in Mothers **eTable 12.** Adverse Events in Infants **eTable 13.** Characteristics of Participants Who Died on Study by Cardiac Diagnosis and Treatment **eFigure 1.** Cumulative Mortality **eFigure 2.** Common Maternal Adverse Events **eFigure 3.** Common Infant Adverse Events

Supplement 2.
Trial Protocol and Statistical Analysis Plan

Supplement 3.
Data Sharing Statement
