# Supplementary material for: Progesterone for Neurodevelopment in Fetuses With Congenital Heart Defects: A Randomized Clinical Trial
Source: JAMA Netw Open. 2024 May 28;7(5):e2412291. doi: 10.1001/jamanetworkopen.2024.12291 (PMC11134212; doi:10.1001/jamanetworkopen.2024.12291)
Supplement: Supplement 1. — eMethods. eReferences eTable 1. Baseline Characteristics of Mothers and Fetuses Stratified by Cardiac Diagnosis eTable 2. Cardiac Class and Diagnosis eTable 3. Genetic Evaluation eTable 4. Reasons for Drug Termination Before 39 Weeks GA eTable 5. Delivery and Neonate Characteristics eTable 6. Operative Characteristics eTable 7. BSID-III Scales Overall and by Pre-specified Subgroups eTable 8. Treatment Effect Estimates for BSID-III Scales Using Linear Model Adjusted for Pre-specified Covariates eTable 9. Treatment Effect Estimates for BSID-III Scales Using Multiple Imputation to Address Incomplete Outcome Data eTable 10. Per-Protocol Analysis eTable 11. Adverse Events in Mothers eTable 12. Adverse Events in Infants eTable 13. Characteristics of Participants Who Died on Study by Cardiac Diagnosis and Treatment eFigure 1. Cumulative Mortality eFigure 2. Common Maternal Adverse Events eFigure 3. Common Infant Adverse Events [file jamanetwopen-e2412291-s001.pdf]

Supplemental Online Content

Gaynor JW, Moldenhauer JS, Zullo EE, et al. Progesterone for neuroprotection in fetuses with congenital heart defects: a randomized clinical trial. *JAMA Netw Open.* 2024;7(5):e2412291. doi:10.1001/jamanetworkopen.2024.12291

eMethods

eReferences

eTable 1. Baseline Characteristics of Mothers and Fetuses Stratified by Cardiac Diagnosis

eTable 2. Cardiac Class and Diagnosis

eTable 3. Genetic Evaluation

eTable 4. Reasons for Drug Termination Before 39 Weeks GA

eTable 5. Delivery and Neonate Characteristics

eTable 6. Operative Characteristics

eTable 7. BSID-III Scales Overall and by Pre-specified Subgroups

eTable 8. Treatment Effect Estimates for BSID-III Scales Using Linear Model Adjusted for Pre-specified Covariates

eTable 9. Treatment Effect Estimates for BSID-III Scales Using Multiple Imputation to Address Incomplete Outcome Data

eTable 10. Per-Protocol Analysis

eTable 11. Adverse Events in Mothers

eTable 12. Adverse Events in Infants

eTable 13. Characteristics of Participants Who Died on Study by Cardiac Diagnosis and Treatment

eFigure 1. Cumulative Mortality

eFigure 2. Common Maternal Adverse Events

eFigure 3. Common Infant Adverse Events

This supplemental material has been provided by the authors to give readers additional information about their work.

© 2024 Gaynor JW et al. *JAMA Network Open.*

1 eMethods

1.1 Exclusion Criteria

The exclusion criteria were: 1) major genetic or extra-cardiac anomaly other than 22q11 deletion; 2) language other than English spoken at home; 3) known sensitivity or listed contraindication to progesterone (known allergy or hypersensitivity to progesterone, severe hepatic dysfunction, undiagnosed vaginal bleeding, mammary or genital tract carcinoma, thrombophlebitis, thromboembolic disorders, cerebral hemorrhage, porphyria); 4) prescription or ingestion of medications known to interact with progesterone (e.g. bromocriptine, rifamycin, ketoconazole or cyclosporin); 5) maternal use of progesterone within 30 days of enrollment; 6) history of preterm birth or short cervix (defined as cervical length ≤ 25 mm at 18-24 weeks GA necessitating progesterone therapy; 7) multiple gestation; 8) maternal contraindication for magnetic resonance imaging (MRI); and 9) participants with a known history of non-compliance with medical therapy.

1.2 Family Reimbursement

For three study-related visits, families received financial reimbursement for per diem expenses, travel expenses (airfare, hotel, mileage, parking, tolls, etc.), and childcare. The family was additionally given a gift card ($50) after each visit.

1.3 Enrollment, Randomization, Study Drug Preparation, Administration and Adherence

A study nurse met weekly with the Fetal Heart Program to identify potential subjects. Potential subjects were screened for eligibility at 24-28 weeks GA by a study nurse using the protocol inclusion and exclusion criteria. Permission to review the research study in person was asked of eligible subjects at a prenatal visit. If subjects agreed to a research review, the informed consent process was initiated to assure subjects were able to comprehend the purpose of the study, the study procedures, and the risk-benefit profile.

© 2024 Gaynor JW et al. *JAMA Network Open.*

Participants who agreed to participate were 1:1 block-randomized to progesterone or placebo by fetal CHD diagnosis (hypoplastic left heart syndrome (HLHS), transposition of the great arteries (TGA), and other CHD (OTHER). The block size was four. The randomization scheme was created by the study statistician (MP) and uploaded into a REDCap database. Independent of the study team, a research pharmacist used the randomization table to assign eligible participants to either progesterone (8% gel) or placebo, a vaginal moisturizer. The study was double-blinded throughout; the statisticians were blinded to the identity of the intervention arms until the interim analysis requested by the DSMB in January 2020. The mothers received care in the Richard D Wood Jr. Center for Fetal Diagnosis and Treatment at the Children's Hospital of Philadelphia with planned delivery in the Garbose Family Special Delivery Unit.

1.4 Study Drug Preparation, Administration and Adherence

The treatments were packaged in identical prefilled applicators (Progesterone, 8% gel equivalent to 90 mg /applicator). Participants received the initial dispense of study drug at their initial visit and then monthly. Drug administration was initiated prior to 28 weeks GA. For this study, up to 28 weeks 3 days was considered as 28 weeks GA. Participants were to administer the medication vaginally twice daily until 39 weeks gestational age (GA) or delivery of the baby, whichever happened first. Participants were asked to record the date and time of each dose along with any side effects in a study diary. The study team met with mothers during each prenatal visit to dispense study drug, collect unused applicators, and answer questions. For study-related visits, families received financial reimbursement.

Adherence to the study drug administration was based on the returned applicators and the diary. Patients were asked to return all unused medication applicators at each prenatal visit. The number of doses taken was determined from the difference between the number of doses

© 2024 Gaynor JW et al. *JAMA Network Open.*

dispensed and returned unused. If a subject did not return their unused applicators, the number of doses taken was determined from their diaries. If the medication was stopped before 39 weeks GA due to delivery or study termination, the calculation was based on the total number of doses available from study enrollment up to medication termination. For 92 participants (90%), adherence was calculated as the percentage of doses taken (the number of doses available up until medication termination dispensed minus the number of doses returned) divided by the number of possible doses. Occasionally (n=7, 7%), calculated adherence exceeded 100%, apparently because some unused applicators were not returned after withdrawal or delivery. In this case, the reported adherence was truncated to 100%. For one subject who did not return any applicators, adherence was estimated solely based on the diary. Two participants did not return applicators or a diary and adherence could not be calculated. Adverse events were monitored throughout the study by regular contact with the participants, patient diaries, and review of the medical record. Maternal adverse event terms and grades were monitored and collected using the Common Terminology Criteria for Adverse Events, version 4.0 (CTCAE v.4) developed by the National Institutes of Health. Child adverse events were collected using an adverse event reporting system developed by the Pediatric Heart Network (established by the National Heart, Lung, and Blood Institute) for reporting adverse events in clinical trials for complex congenital heart disease.[5]

1.5 Delivery and Neonatal Hospitalization

After delivery, a cord blood sample was collected for genetic testing. If cord blood was not available, a sample was obtained at a later date. The baby was transferred to the Cardiac Intensive Care Unit. Post-natal care was determined by clinicians caring for the child. A CHOP-based team of dedicated pediatric cardiac surgeons and anesthesiologists performed

© 2024 Gaynor JW et al. *JAMA Network Open.*

all operations, except for two participants born unexpectedly at outside hospitals. Deep hypothermic circulatory arrest (DHCA) was used at the surgeon's discretion. Patients undergoing cardiopulmonary bypass (CPB) received modified ultrafiltration.

Demographic and clinical data were abstracted from patient records. Newborn length, weight, and head circumference were collected, and the z-score for each growth measurement was calculated using World Health Organization (WHO) standards for full-term infants and the Fenton growth chart for preterm infants.[1,2] Maternal education, socioeconomic-related variables that comprise the Hollingshead socioeconomic status (SES), and race/ethnicity were determined through parental report.[3] Impaired maternal-fetal environment (MFE) indicates a mother with a history of one or more of the following: gestational hypertension, gestational diabetes, tobacco use or hypothyroidism at baseline or pre-eclampsia during the pregnancy. In addition to the fetal CHD diagnosis, patients were assigned to a previously described classification based on neonatal physiology: Class I is 2 ventricle CHD with no arch obstruction, Class II is 2 ventricle CHD with arch obstruction, Class III is single ventricle CHD with no arch obstruction, and Class IV is single ventricle CHD with arch obstruction.[4] This classification has been shown to correlate with risk of mortality and with neurodevelopmental outcomes.

1.6 Genetic Evaluation and APOE Genotyping

Clinical genetic testing was performed as indicated. While major genetic anomalies were an exclusion criterion at enrollment, universal prenatal recognition of these anomalies is difficult. Thus, some enrolled infants received the diagnosis of a genetic syndrome only after the 18-month evaluation or by genetic testing. Whole exome sequencing and a microarray were performed on the participants, if not obtained clinically. Pathologic or likely pathologic genetic syndromes or chromosomal abnormalities were identified. Based on the clinical evaluation and

© 2024 Gaynor JW et al. *JAMA Network Open.*

genetic testing, patients were classified as normal (no pathogenic or likely pathogenic variants identified), as abnormal (pathogenic or likely pathogenic variants related to CHD or neurodevelopment identified), or as suspect (variants of unknown significance were identified or there were concerns on the clinical examination). Variant calls were queried for single nucleotide polymorphisms to determine apo-lipoprotein E (*APOE*) genotype. Variant call files were obtained from the clinical and research testing laboratories filtered using BCFtools v1.9 to contain only two variants, rs429358 and rs7412. *APOE* haplotypes were subsequently defined based on individual rs429358/rs7412 genotypes as follows: C/T, ε1; T/T, ε2; T/C, ε3; C/C, ε4. Patients were classified as ε2 (ε2ε2 and ε2ε3), ε3 (ε3ε3), or ε4 (ε3ε4 and ε4ε4). Patients with ε2ε4 were excluded.

1.7 Outcome Variables

Neurodevelopmental outcomes were assessed using The Bayley Scales of Infant and Toddler Development-III; which provide composite scores for Cognitive, Language and Motor Scales ($\mu = 100$ with $\sigma = 15$). For the Motor and Language composites, there are component scores: Motor (Fine and Gross) and Language (Expressive and Receptive), ($\mu = 10$ with $\sigma = 3$). Higher scores indicate better skills. [5]

Additional variables of interest pre-specified prior to unblinding and data analysis include: GA of neonate at time of birth (both continuous and whether born earlier than 37 weeks GA, delivery type (vaginal or cesarean-section, either elective or emergent), placental weight and placental weight <10th percentile, birthweight/placental weight ratio, presence of placental infarction, placental fetal or maternal vascular malperfusion and placental fetal vascular thrombi, cardiac operation during first admission, age at first operation, use of cardiopulmonary bypass (CPB), weight at first operation, Cardiac Class, deep hypothermia

© 2024 Gaynor JW et al. *JAMA Network Open.*

circulatory arrest (DHCA) time, total support time (TST), antegrade cerebral perfusion time, need for extracorporeal membrane oxygenation (ECMO), additional operations with CPB, additional TST, additional DHCA, mortality (in-hospital, on-study and post-study, length of stay and selected complications for first surgical admission.

2 Results

2.1 Per Protocol Analyses

Results for participants who used at least 80% or 90% of their intended dose for a 39-week pregnancy appears in Supplemental Table S9. Per-protocol models were adjusted for diagnosis, sex, and presence of genetic anomalies. In the ≥90% group, the mean improvement for the Motor Score for progesterone versus placebo was 4.0 units (90% CI -1.0, 8.9). For Language, the mean improvement after adjustment was 1.76 (90% CI -5.2, 8.7) and for Cognition 0.37 units (90% CI -5.1, 5.9). Results were similar for ≥ 80%.

3 eReferences

1. de Onis M, Garza C, Victoria C, Onyango A, Frongillo E, Martines J. The WHO Multicentre Growth Reference Study: Planning, Study Design, and Methodology. *Food and Nutrition Bulletin*. 2004;25:S15-26.
2. Fenton TR, Kim JH. A systematic review and meta-analysis to revise the Fenton growth chart for preterm infants. *BMC Pediatrics*. 2013;13. doi: https://doi.org/10.1186/1471-2431-13-59
3. Hollingshead AB. *Four factor index of social status*. New Haven, Connecticut: Department of Sociology, Yale University; 1975.
4. Clancy RR, McGaurn SA, Wernovsky G, Spray TL, Norwood WI, Jacobs ML, Murphy JD, Gaynor JW, Goin JE. Preoperative risk-of-death prediction model in heart surgery with deep hypothermic circulatory arrest in the neonate. *J Thorac Cardiovasc Surg*. 2000;119:347-357. doi: 10.1016/S0022-5223(00)70191-7.

© 2024 Gaynor JW et al. *JAMA Network Open.*

5. Virzi L, Pemberton V, Ohye RG, Tabbutt S, Lu M, Atz TC, Barnard T, Dunbar-Masterson C, Ghanayem NS, Jacobs JP, et al. Reporting adverse events in a surgical trial for complex congenital heart disease: the Pediatric Heart Network experience. J Thorac Cardiovasc Surg. 2011;142:531-537. doi: 10.1016/j.jtcvs.2010.11.052

6. Bayley N. *The Bayley Scales of Infant Development-III*. San Antonio, TX: The Psychological Corporation Google Scholar Open URL query; 2006

© 2024 Gaynor JW et al. *JAMA Network Open.*

4 Supplemental Tables

eTable 1: Baseline Characteristics of Mothers and Fetuses Stratified by Cardiac Diagnosis

	**HLHS**		**Other**		**TGA**	
	Progesterone (N=27)	Placebo (N=25)	Progesterone (N=6)	Placebo (N=6)	Progesterone (N=19)	Placebo (N=19)
	N (%)[a]		N (%)[a]		N (%)[a]	
**Maternal Participants**						
**Age (years)**						
Median [IQR]	31.0 [26.0, 34.0]	29.0 [27.0, 33.0]	31.0 [26.75, 34.5]	33.0 [32.0, 34.0]	33.0 [27.5, 34.0]	32.0 [30.0, 33.0]
**Race**						
White	16 (59.3%)	19 (76.0%)	4 (66.7%)	5 (83.3%)	16 (84.2%)	17 (89.5%)
Black	5 (18.5%)	3 (12.0%)	1 (16.7%)	0 (0%)	0 (0%)	0 (0%)
Asian	2 (7.4%)	1 (4.0%)	1 (16.7%)	1 (16.7%)	1 (5.3%)	1 (5.3%)
American Indian/ Alaskan Native	2 (7.4%)	0 (0%)	0 (0%)	0 (0%)	0 (0%)	0 (0%)
Unknown	0 (0%)	1 (4.0%)	0 (0%)	0 (0%)	0 (0%)	1 (5.3%)
Mixed Race	2 (7.4%)	1 (4.0%)	0 (0%)	0 (0%)	2 (10.5%)	0 (0%)
**Hispanic Ethnicity**						
Yes	2 (7.4%)	2 (8.0%)	0 (0%)	1 (16.7%)	1 (5.3%)	2 (10.5%)
No	24 (88.9%)	21 (84.0%)	6 (100%)	5 (83.3%)	18 (94.7%)	17 (89.5%)
Unknown	1 (3.7%)	2 (8.0%)	0 (0%)	0 (0%)	0 (0%)	0 (0%)
**Education**						
High School	5 (18.5%)	3 (12.0%)	0 (0%)	0 (0%)	2 (10.5%)	0 (0%)
College	12 (44.4%)	18 (72.0%)	3 (50.0%)	4 (66.7%)	8 (42.1%)	11 (57.9%)

© 2024 Gaynor JW et al. *JAMA Network Open.*

eTable 1: Baseline Characteristics of Mothers and Fetuses Stratified by Cardiac Diagnosis

	HLHS		Other		TGA	
Post graduate degree	10 (37.0%)	4 (16.0%)	3 (50.0%)	2 (33.3%)	9 (47.4%)	8 (42.1%)
**Income**						
<$50,000	6 (22.2%)	4 (16.0%)	1 (16.7%)	0 (0%)	0 (0%)	0 (0%)
$50,000-$100,000	8 (29.6%)	7 (28.0%)	1 (16.7%)	3 (50.0%)	5 (26.3%)	6 (31.6%)
>$100,000	8 (29.6%)	10 (40.0%)	3 (50.0%)	3 (50.0%)	8 (42.1%)	12 (63.2%)
Unknown	5 (18.5%)	4 (16.0%)	1 (16.7%)	0 (0%)	6 (31.6%)	1 (5.3%)
**Impaired Maternal Fetal Environment**[b]						
Yes	10 (37.0%)	11 (44.0%)	2 (33.3%)	2 (33.3%)	7 (36.8%)	4 (21.1%)
No	17 (63.0%)	14 (56.0%)	4 (66.7%)	4 (66.7%)	12 (63.2%)	15 (78.9%)
**Fetal Participants**[c]						
**Sex**						
Male	18 (66.7%)	14 (56.0%)	4 (66.7%)	4 (66.7%)	14 (73.7%)	13 (68.4%)
Female	9 (33.3%)	11 (44.0%)	2 (33.3%)	2 (33.3%)	5 (26.3%)	6 (31.6%)
**Genetic Classification** [d]						
Normal	17 (63.0%)	17 (68.0%)	2 (33.3%)	3 (50.0%)	10 (52.6%)	12 (63.2%)
Suspect	8 (29.6%)	3 (12.0%)	0 (0%)	0 (0%)	4 (21.1%)	2 (10.5%)
Abnormal	2 (7.4%)	4 (16.0%)	4 (66.7%)	3 (50.0%)	3 (15.8%)	4 (21.1%)
Unknown	0 (0%)	1 (4.0%)	0 (0%)	0 (0%)	2 (10.5%)	1 (5.3%)
**22q11.2 deletion** [e]						
Yes	0 (0%)	0 (0%)	4 (66.7%)	1 (16.7%)	0 (0%)	0 (0%)
No	27 (100%)	24 (96.0%)	2 (33.3%)	5 (83.3%)	17 (89.5%)	18 (94.7%)
Unknown	0 (0%)	1 (4.0%)	0 (0%)	0 (0%)	2 (10.5%)	1 (5.3%)

© 2024 Gaynor JW et al. *JAMA Network Open.*

eTable 1: Baseline Characteristics of Mothers and Fetuses Stratified by Cardiac Diagnosis

	HLHS		Other		TGA	
**APOE Genotype[d,3]**						
ε2	5 (18.5%)	4 (16.0%)	2 (33.3%)	1 (16.7%)	1 (5.3%)	1 (5.3%)
ε2/ε4	0 (0%)	1 (4.0%)	0 (0%)	0 (0%)	0 (0%)	0 (0%)
ε3	12 (44.4%)	14 (56.0%)	1 (16.7%)	3 (50.0%)	13 (68.4%)	13 (68.4%)
ε4	8 (29.6%)	4 (16.0%)	3 (50.0%)	2 (33.3%)	3 (15.8%)	4 (21.1%)
Missing	2 (7.4%)	2 (8.0%)	0 (0%)	0 (0%)	2 (10.5%)	1 (5.3%)

[a] N (%) represented unless otherwise indicated.

[b]Impaired MFE indicates mother with one or more of gestational hypertension, gestational diabetes, tobacco use or hypothyroidism at baseline or pre-eclampsia during the pregnancy.

[c] Thirty-three participants were Cardiac Class I; 13 participants were Class II; two s were Class III; 50 were Cardiac Class IV. (See Supplemental Table S2)

[d]Genetic classification, 22q11.2 deletion, and APOE genotype are often not known until after birth. Three participants withdrew before birth, contributing to the missing data.

[e] ε2 indicates homozygous for ε2 or heterozygous ε2/ ε3. ε3 indicates homozygous for ε3. ε4 indicates homozygous for ε4 or heterozygous ε4/ε3.

© 2024 Gaynor JW et al. *JAMA Network Open.*

eTable 2: Cardiac Class and Diagnosis

Cardiac Diagnosis	Cardiac Class			
	1 (N=33)	2 (N=13)	3 (N=2)	4 (N=50)
**HLHS [a]**	0 (0%)	1 (7.7%)	1 (50.0%)	49(98.0%)
**OTHER**	1 (3.0%)	10 (76.9%)	0 (0%)	1 (2.0%)
**TGA [b]**	32 (97.0%)	2 (15.4%)	1 (50.0%)	0 (0%)

The assignment to HLHS, TGA, or OTHER was based on fetal echocardiography prior to 28 weeks gestational age. Cardiac Class was assigned post-natally based on clinical findings and management. However, there may be changes over the third trimester of pregnancy and with better post-natal assessment. One subject assigned to HLHS underwent a 2-ventricular repair (Class II) and one had single ventricle physiology without arch obstruction (Class III). Also, two of the subjects assigned to TGA, had TGA/VSD with arch obstruction not appreciated at the time of randomization. Also, the majority of the OTHER patients had arch hypoplasia/obstruction with anatomy suitable for 2-ventricular repair (Class II). One was found to have single ventricle physiology with arch obstruction after birth and underwent a Norwood procedure (Class IV).

[a] One fetus in the HLHS category died prior to the surgery necessary to delegate which cardiac class it would have been assigned.

[b] Three TGA fetus' withdrew prior to the study initiation.

© 2024 Gaynor JW et al. *JAMA Network Open.*

eTable 3: Results of Genetic Evaluation and Testing

Genetic Evaluation	Genetic Testing	Genetic Classification
ABN	Deletion 22q11	Abnormal
ABN	Deletion 22q11	Abnormal
ABN	Deletion 22q11	Abnormal
ABN	Deletion 22q11	Abnormal
ABN	Deletion 22q11	Abnormal
Suspect	De novo variant EP300	Abnormal
Suspect	Duplication 22q13.31q13.33	Abnormal
NL	2q13 deletion	Abnormal
Suspect	Factor V Leiden	Abnormal
NL	Factor V Leiden	Abnormal
NL	Likely pathogenic SCN2A variant	Abnormal
ABN	MMP21 missense variant	Abnormal
Suspect	SEMA3E variant	Abnormal
NL	Likely pathogenic GDF1 variant	Abnormal
NL	Likely pathologic variant in DVL3	Abnormal
ABN	Deletion 6p22.3	Abnormal
Suspect	Pathogenic de novo PUM-1 variant	Abnormal
NL	Pathologic Variant in RSPH1	Abnormal
ABN	Deletion 1q21.1q21.2	Abnormal
Suspect	Pathogenic variant CFTR	Abnormal
Suspect	Deletion chromosome 16p12.2, NKX2-6 missense variant VUS	Suspect
Suspect	Deletion 12p12.2p12.1	Suspect
NL	Deletion 15q11.2, VUS in EVC, PRDM6, TFAP2B, A2ML1	Suspect
Suspect		Suspect
Suspect	15q15.3 deletion	Suspect
Suspect	heterozygous CCDC39 VUS	Suspect
NL	heterozygous DNAH11 (VUS)	Suspect
NL	heterozygous GATA 4 VOUS	Suspect
NL	Heterozygous HIPK2 frameshift variant	Suspect
NL	Heterozygous HIPK2 frameshift variant VUS	Suspect
Suspect	Heterozygous KMT2D VUS	Suspect
Suspect	Variant GDF1	Suspect
NL	VUS in A2ML1 and MED13L	Suspect
NL	VUS in KAT6B and DNAH1	Suspect
NL	VUS in RYR2, NRAS, DNAH1	Suspect
NL	VUS in TGFBR1, DMXL2, JAG1	Suspect

© 2024 Gaynor JW et al. *JAMA Network Open.*

Suspect	NA	Suspect
NA	NA	Unknown
Suspect	None	Normal
Suspect	None	Normal
Suspect	None	Normal
Suspect	None	Normal
Suspect	None	Normal
Suspect	None	Normal
Suspect	None	Normal
Suspect	None	Normal
Suspect	None	Normal
NR	None	Normal
NA	None	Normal
NR	None	Normal
NA	None	Normal
NA	None	Normal
Suspect	None	Normal
NL	None	Normal
Suspect	None	Normal
NA	None	Normal
NL	None	Normal
NL	None	Normal
NL	None	Normal
NL	None	Normal
NL	None	Normal
NL	None	Normal
NL	None	Normal
NL	None	Normal
NL	None	Normal
NL	None	Normal
NL	None	Normal
NL	None	Normal
NL	None	Normal
NL	None	Normal
NL	None	Normal
NL	None	Normal
NL	None	Normal
NL	None	Normal
NL	None	Normal
NL	None	Normal
NL	None	Normal
NL	None	Normal
NL	None	Normal

© 2024 Gaynor JW et al. *JAMA Network Open.*

NL	None	Normal
NL	None	Normal
NL	None	Normal
NL	None	Normal
NL	None	Normal
NL	None	Normal
NL	None	Normal
NL	None	Normal
NL	None	Normal
NL	None	Normal
NL	None	Normal
NL	None	Normal
NL	None	Normal
NL	None	Normal
NL	None	Normal
NL	None	Normal
NL	None	Normal
NL	None	Normal
NL	None	Normal
NL	None	Normal

VUS = variant of unknown significance

© 2024 Gaynor JW et al. *JAMA Network Open.*

eTable 4: Reasons for Drug Termination Before 39 Weeks GA

Reason	Progesterone[a] (N=26)	Placebo[a] (N=35)	Percentage Difference (90% CI)[b]
	N (%)	N (%)	
Obstetric Indication for Delivery	6 (23.1%)	17 (48.6%)	-25.5 (-48.3, -2.7)
Maternal Indication for Delivery	6 (23.1%)	2 (5.7%)	17.4 (-1.0, 35.8)
Fetal Indication for Delivery	3 (11.5%)	5 (14.3%)	-2.8 (-19.7, 14.2)
Study Drug AE	7 (26.9%)	4 (11.4%)	15.5 (-4.7, 35.7)
Noncompliance	1 (3.8%)	4 (11.4%)	-7.6 (-21.7, 6.6)
Withdrew	0 (0%)	1 (2.9%)	-2.9 (-10.3, 4.6)
Study halted by DSMB	3 (11.5%)	1 (2.9%)	8.6 (-6, 23.3)
Did not initiate	0 (0%)	1 (2.9%)	-2.9 (-10.3, 4.6)

[a]Percentage of participants who terminated study drug ≤39 weeks GA

[b]90% CI for the percentage difference using a Chi-square distribution

© 2024 Gaynor JW et al. *JAMA Network Open.*

eTable 5: Delivery and Neonate Characteristics

Outcomes[a]	Progesterone (N=52)[c]	Placebo (N=50)[d]	Treatment Effect Mean (90% CI)[b]	P-value
	N (%)[i]	N (%)[i]		
**Delivery Type**				0.85[e]
Vaginal	29 (55.8%)	26 (52.0%)	REF	
Cesarean (Elective)	10 (19.2%)	12 (24.0%)	0.8 [0.3,1.8]	
Cesarean (Emergent)	11 (21.2%)	10 (20.0%)	1.0 [0.4, 2.7]	
**Induction of Labor**				0.043[e]
No	20 (38.5%)	29 (58.0%)	REF	
Yes	30 (57.7%)	19 (38.0%)	2.3 [1.2,4.6]	
**Gestational Age (GA)**				
>=39 wks.	30 (57.7%)	22 (44.0%)	0.55 (0.3, 1.1)	0.13 [f]
37-38 wks. 6d	16 (30.8%)	21 (42.0%)		
32-36 wks. 6d	4(7.7%)	5 (10.0%)		
<32 weeks	0(0%)	1 (2.0%)		
**GA (weeks)**				
Median [IQR]	39.0[38.3, 39.4]	38.9 [38.4, 39.1]	0.3 [-0.2,0.8]	0.35[g]
**Birth Weight (kg)**				
Median [IQR]	3.4[2.9, 3.7]	3.3[3.0, 3.5]	0.8[-1.0,2.7]	0.44[g]
**Birth Weight (Z-scores)**				
Median [IQR]	0.26 [-0.75, 0.76]	-0.06 [-0.59, 0.32]	0.1[-0.2,0.4]	0.57[g]
**Placental Weight (g)**				
Median [IQR]	388 [350, 446]	424 [360, 486]	-15.4 [-46,16]	0.41[g]
**Placental Weight (Z-scores)**				
Median [IQR]	-1.7 [-2.2, -0.9]	-1.1 [-2.0, -0.2]	-0.3 [-0.7,0.1]	0.25[g]
**Placental Weight <10th Percentile**				
>=10th Percentile	19 (36.5%)	26 (52.0%)	REF	

© 2024 Gaynor JW et al. *JAMA Network Open.*

<10th Percentile	29 (55.8%)	22 (44.0%)	1.9 [0.9,3.9]	0.14[eg]
**BW/PW**				
Median [IQR]	8.3 [7.4, 9.4]	7.6 [6.6, 8.5]	1.1 [1.0, 1.2]	0.07[h]

[a] Cord blood progesterone pre-specified but not included due to inconsistent sample collection and differences in timing of blood collection relative to cessation of treatment.

[b] Treatment effect. P-value is for a univariate test or in the case of multiple categories, a global test. Specific model indicated for each feature. All models stratified by cardiac diagnosis.

[c] Two participants (3.8%) had missing data for delivery type, induction of labor, post-menstrual age, birthweight and 4 (7.7%) had missing placental weight and BW:PW. Percentages based on N=52.

[d] Two participants (4%) had missing data for delivery type, induction of labor, birthweight, placental weight, and BW:PW. Postmenstrual age missing in 1 subject. Percentages based on N=50.

[e] Multinomial or logistic regression model. Values are odds ratios for progesterone versus placebo for each category versus the reference group (REF).

[f] Ordinal logistic regression model. The youngest two GA (<32 weeks and 32-37wks 6 d) were combined for the regression model. The estimate is the odds of falling into the lower age categories for progesterone versus placebo e.g., the odds of being in a GA category less than 39 weeks is 0.6-fold smaller for progesterone versus placebo.

[g] Linear regression model. Values are mean differences for progesterone vs placebo.

[h] Linear regression model using log-transformed outcome variable. Values are ratio of BW:PW for progesterone vs placebo.

[I] Unit of measurement (N %) unless otherwise indicated.

© 2024 Gaynor JW et al. *JAMA Network Open.*

eTable 6: Operative Characteristics

Features[a]	Progesterone[b] (N=52)	Placebo[b] (N=50)	Overall[b] (N=102)
	N (%)[c]	N (%)[c]	N (%)[c]
**Cardiac Class**			
1	16 (30.8%)	17 (34.0%)	33 (32.4%)
2	7 (13.5%)	6 (12.0%)	13 (12.7%)
3	0 (0%)	2 (4.0%)	2 (2.0%)
4	27 (51.9%)	23 (46.0%)	50 (49.0%)
**Cardiac Operation During First Admission**			
Yes	49 (94.2%)	47 (94.0%)	96 (94.1%)
No	1 (1.9%)	2 (4.0%)	3 (2.9%)
**Weight at First Operation (kg)**			
Median [IQR]	3.50 [3.0, 3.7]	3.30 [3.1, 3.6]	3.40 [3.0, 3.7]
**Age at First Operation (days)**			
Median [IQR]	4.0 [2.0, 5.0]	4.0 [3.0, 5.0]	4.0 [3.0, 5.0]
**CPB [d] Used During First Operation**			
Yes	45 (86.5%)	43 (86.0%)	88 (86.3%)
No	5 (9.6%)	5 (10.0%)	10 (9.8%)
**TST for First Operation**			
Median [IQR]	88.5 [74.2, 104.5]	83.5 [64.8, 92.0]	85.0 [69.2, 101.5]
**Received DHCA [e]**			
No	20 (38.5%)	23 (46.0%)	43 (42.2%)
Yes	30 (57.7%)	25 (50.0%)	55 (53.9%)
**DHCA [e] time among those receiving DHCA**			
Median [IQR]	46.0 [38.2, 53.8]	39.0 [33.0, 46.0]	43.0 [35.0, 49.0]
**ECMO[f] Before 18 months**			
Yes	7 (13.5%)	4 (8.0%)	11 (10.8%)

© 2024 Gaynor JW et al. *JAMA Network Open.*

No	43 (82.7%)	44 (88.0%)	87 (85.3%)
**Length of Stay for First Operation**			
Median [IQR]	22.5 [14.2, 32.8]	17.5 [13.0, 24.8]	19.0 [14.0, 29.8]
**Number Additional Cardiac Operations with CPB[c] Before 18 Months**			
0	18 (34.6%)	24 (48.0%)	42 (41.2%)
1	25 (48.1%)	21 (42.0%)	46 (45.1%)
2	7 (13.5%)	4 (8.0%)	11 (10.8%)
**Received Additional TST [g]**			
No	18 (34.6%)	24 (48.0%)	42 (41.2%)
Yes	32 (61.5%)	25 (50.0%)	57 (55.9%)
**Received Additional DHCA [e]**			
No	37 (71.2%)	35 (70.0%)	72 (70.6%)
Yes	13 (25.0%)	14 (28.0%)	27 (26.5%)
**DHCA [e] Among those with Additional DHCA**			
Median [IQR]	30.0 [19.0, 44.0]	43.5 [27.8, 53.0]	37.0 [24.5, 52.0]
**TST [g] among those with Additional TST**			
Median [IQR]	64.0 [42.0, 118.8]	80.0 [67.0, 119.0]	72.0 [53.0, 119.0]

[a]Two participants had antegrade cerebral perfusion time for first operation. Values were 49 minutes (placebo) and 56 minutes (progesterone).

[b]Three participants with TGA (2 on progesterone and 1 on placebo) and one subject with HLHS (placebo) were missing operative data. These participants were included in the denominator when calculating percentages.

[c] Unit of measurement N (%) unless otherwise indicated.[d] CPB - Cardiopulmonary Bypass

[e] DHCA - Deep Hypothermia Circulatory Arrest

[f] ECMO - Extracorporeal Membrane Oxygenation

[g] TST - Total Support Time

© 2024 Gaynor JW et al. *JAMA Network Open.*

eTable 7: BSID-III Scales Overall and by Pre-specified Subgroups

Values shown are median [IQR]. N is number of participants in the group.

Subgroup	Feature	Test Group	Progesterone	Placebo
**All**		N	43	42
		Motor	91.0 [85.0, 97.0]	92.50 [82.0, 97.0]
		Language	84.5 [76.2, 94.8]	89.00 [71.75, 99.25]
		Cognitive	95.0 [83.8, 96.25]	90.00 [86.25, 98.75]
**Diagnosis**	HLHS	N	23	19
		Motor	91.0 [82.0, 97.0]	88.0 [79.0, 94.0]
		Language	86.0 [78.0, 93.0]	79.0 [68.0, 92.5]
		Cognitive	95.0 [85.0, 97.5]	90.00 [80.0, 92.5]
	OTHER	N	5	6
		Motor	94.0 [88.0, 97.0]	80.5 [71.5, 85.0]
		Language	97.0 [79.0, 103.0]	75.5 [61.25, 87.5]
		Cognitive	90.0 [90.0, 95.0]	92.5 [82.5, 98.75]
	TGA	N	15	17
		Motor	94.0 [89.5, 97.0]	94.0 [91.0, 97.0]
		Language	81.0 [73.25, 91.75]	94.0 [83.0, 106.0]
		Cognitive	90.0 [80.0, 96.25]	95.0 [90.0, 100.0]
**Fetal Sex**	Female	N	15	14
		Motor	91.0 [85.0, 97.0]	85.0 [70.75, 96.25]
		Language	86.0 [79.0, 95.5]	72.5 [62.75, 86.5]
		Cognitive	95.0 [82.50, 95.0]	90.0 [76.25, 95.0]
	Male	N	28	28
		Motor	92.5 [83.5, 100.0]	94.0 [85.0, 97.0]
		Language	82.0 [74.0, 94.0]	91.0 [82.75, 100.75]
		Cognitive	95.0 [85.0, 100.0]	92.5 [90.0, 100.0]

© 2024 Gaynor JW et al. *JAMA Network Open.*

eTable 7: BSID-III Scales Overall and by Pre-specified Subgroups

Values shown are median [IQR]. N is number of participants in the group.

Subgroup	Feature	Test Group	Progesterone	Placebo
**Genetic Classification**	Normal	N	23	27
		Motor	94.0 [91.0, 98.5]	94.0 [83.5, 97.0]
		Language	87.5 [78.5, 94.0]	83.0 [72.5, 98.5]
		Cognitive	95.0 [90.0, 100.0]	95.0 [90.0, 95.0]
	Suspect	N	12	5
		Motor	94.0 [78.25, 97.75]	94.0 [88.0, 94.0]
		Language	87.5 [78.5, 103.75]	91.0 [89.0, 106.0]
		Cognitive	95.0 [88.75, 102.5]	105.0 [90.0, 105.0]
	Abnormal	N	8	10
		Motor	80.5 [66.25, 85.75]	82.0 [76.75, 91.75]
		Language	71.0 [67.25, 79.0]	86.0 [59.75, 94.0]
		Cognitive	77.5 [75.0, 82.5]	90.0 [81.25, 97.5]
**Impaired MFE**	Unimpaired	N	31	27
		Motor	94.0 [85.0, 97.0]	94.0 [82.0, 97.0]
		Language	86.0 [75.5, 97.0]	89.0 [76.5, 104.5]
		Cognitive	95.0 [85.0, 95.0]	95.0 [90.0, 100.0]
	Impaired	N	12	15
		Motor	91.0 [80.5, 97.75]	85.0 [80.5, 94.0]
		Language	79.0 [77.0, 89.0]	83.0 [57.5, 95.5]
		Cognitive	90.0 [80.0, 100.0]	90.0 [77.5, 92.5]

Two participants returned for the 18-month follow-up visit but were missing all BSID-III Scores. An additional subject returned for follow-up but had no BSID-III Motor Score.

© 2024 Gaynor JW et al. *JAMA Network Open.*

© 2024 Gaynor JW et al. *JAMA Network Open.*

eTable 8: Treatment Effect Estimates for BSID-III Scales Using Linear Model Adjusted for Pre-specified Covariates

BSID-III Score	Treatment Effect	
	Mean (90% CI)[a]	P-value[b]
**Motor**	3.4 [-0.9,7.6]	0.19
**Language**	1.7 [-4.3,7.8]	0.64
**Cognitive**	1.6 [-2.6, 5.7]	0.53

[a] Mean difference between Progesterone and Placebo adjusted for baseline variables of cardiac diagnosis, sex, genetic classification, race, income.

[b] P-value from Wald Test from linear model

© 2024 Gaynor JW et al. *JAMA Network Open.*

eTable 9: Treatment Effect Estimates for BSID-III Scales Using Multiple Imputation to Address Incomplete Outcome Data[a]

BSID-III Scale	Pooled Across All Diagnoses[b]		Stratified by Diagnosis[c]	
	Mean Difference (90% CI)	P-Value	Mean Difference (90% CI)	P-Value
**Motor**	2.8(-1.6,7.2)	0.29	2.9(-1.5,7.2)	0.28
**Language**	1.4(-4.7,7.5)	0.70	1.5(-4.6,7.6)	0.69
**Cognitive**	1.4(-2.9,5.7)	0.58	1.5(-2.7,5.7)	0.56

[a]Variables in the imputation model were cardiac diagnosis, sex, genetic abnormality, and impaired maternal fetal environment

[b]Data pooled across all diagnoses

[c]Analyzed using a linear model stratified by diagnosis

© 2024 Gaynor JW et al. *JAMA Network Open.*

eTable 10: Per-Protocol Analysis

BSID-III Score	Mean (SD)[a]		Adjusted Treatment Effect[a]		
	Progesterone	Placebo	Mean	(90% CI)	P-value
**Per-protocol ≥80% Adherence (n=68-69)**					
**Motor**	89.5(14.9)	86.3(13.5)	3.4	(-1.4,8.2)	0.24
**Language**	86.3(12.5)	83.6(17.6)	1.8	(-4.6,8.1)	0.64
**Cognitive**	91.5(2.2)	91.1(11.6)	0.1	(-4.7,4.9)	0.97
**Per-protocol ≥90% Adherence (n=54-55)**					
**Motor**	91.1(11.6)	86.2(12.2)	4.0	(-1.0,8.9)	0.18
**Language**	88.3(15.2)	84.0(16.6)	1.8	(-5.2,8.7)	0.67
**Cognitive**	92.2(13.1)	91.0(10.4)	0.4	(-5.1,5.9)	0.91

[a]Analyzed using a linear model stratified by diagnosis, with sex, MFE, and genetic profile as additional covariates.

© 2024 Gaynor JW et al. *JAMA Network Open.*

eTable 11: Adverse Events in Mothers

Features	Progesterone (N=279)	Placebo (N=261)	Overall (N=540)
	N (%)[a]	N (%)[a]	N (%)[a]
**Infant Cardiac Diagnosis**			
HLHS	165 (59.1%)	117 (44.8%)	282 (52.2%)
OTHER	35 (12.5%)	23 (8.8%)	58 (10.7%)
TGA	79 (28.3%)	121 (46.4%)	200 (37.0%)
**Serious AE Needing Expedited Reporting**			
Yes	0 (0%)	3 (1.1%)	3 (0.6%)
No	279 (100%)	258 (98.9%)	537 (99.4%)
**AE Relationship to Treatment**			
Not Related	116 (41.6%)	130 (49.8%)	246 (45.6%)
Possibly	163 (58.4%)	131 (50.2%)	294 (54.4%)
**AE Expectedness**			
Expected	278 (99.6%)	256 (98.1%)	534 (98.9%)
Unexpected	1 (0.4%)	5 (1.9%)	6 (1.1%)
**AE Outcome**			
Resolved	276 (98.9%)	261 (100%)	537 (99.4%)
Ongoing	3 (1.1%)	0 (0%)	3 (0.6%)
Death	0 (0%)	0 (0%)	0 (0%)

[a] Unit of measurement N (%) unless otherwise indicated.

© 2024 Gaynor JW et al. *JAMA Network Open.*

eTable 12: Adverse Events in Infants

Features	Progesterone (N=534)	Placebo (N=479)	Overall (N=1013)
	N (%)[a]	N (%)[a]	N (%)[a]
**Cardiac Diagnosis**			
HLHS	306 (57.3%)	322 (67.2%)	628 (62.0%)
OTHER	46 (8.6%)	39 (8.1%)	85 (8.4%)
TGA	182 (34.1%)	118 (24.6%)	300 (29.6%)
**Type of Event**			
AE	395 (74.0%)	354 (73.9%)	749 (73.9%)
SAE	139 (26.0%)	125 (26.1%)	264 (26.1%)
**Serious AE Needing Expedited Reporting**			
Yes	0 (0%)	0 (0%)	0 (0%)
No	534 (100%)	479 (100%)	1013 (100%)
**AE Severity**			
Mild	351 (65.7%)	306 (63.9%)	657 (64.9%)
Moderate	179 (33.5%)	166 (34.7%)	345 (34.1%)
Severe	4 (0.7%)	7 (1.5%)	11 (1.1%)
**AE Relationship to Treatment**			
Not Related	407 (76.2%)	377 (78.7%)	784 (77.4%)
Possibly	127 (23.8%)	102 (21.3%)	229 (22.6%)
**AE Expectedness**			
Expected	533 (99.8%)	472 (98.5%)	1005 (99.2%)
Unexpected	1 (0.2%)	7 (1.5%)	8 (0.8%)
**AE Outcome**			
Resolved	510 (95.5%)	441 (92.1%)	951 (93.9%)
Ongoing	13 (2.4%)	9 (1.9%)	22 (2.2%)
Death	11 (2.1%)	29 (6.1%)	40 (3.9%)

© 2024 Gaynor JW et al. *JAMA Network Open.*

[a] Unit of measurement N (%) unless otherwise indicated.

© 2024 Gaynor JW et al. *JAMA Network Open.*

eTable 13: Characteristics of Participants Who Died on Study by Cardiac Diagnosis and Treatment

Variables	HLHS Progesterone (N=2)	HLHS Placebo (N=5)	TGA Progesterone (N=1)	Overall Progesterone (N=3)	Overall Placebo (N=5)
**Death (age in Days)**					
Median	40.5	75	28	28	75
[Min, Max]	[11, 70]	[1, 198]	[28, 28]	[11, 70]	[1, 198]
**Gestational Age (wks.)**					
Median	35.1	36.7	39.1	36.9	36.7
[Min, Max]	[33.3, 36.9]	[30.6, 38.9]	[39.1, 39.1]	[33.3, 39.1]	[30.6, 38.9]
**Sex**					
Female	0 (0%)	3 (60.0%)	0 (0%)	0 (0%)	3 (60.0%)
Male	2 (100%)	2 (40.0%)	1 (100%)	3 (100%)	2 (40.0%)
**Length of Stay (Days, First Operation)**					
Median	37	69.5	27	27	69.5
[Min, Max]	[10, 64]	[22, 198]	[27, 27]	[10, 64]	[22, 198]
**Genetic Classification**					
Normal	2 (100%)	3 (60.0%)	1 (100%)	3 (100%)	3 (60.0%)
Suspect	0 (0%)	0 (0%)	0 (0%)	0 (0%)	0 (0%)
Abnormal	0 (0%)	1 (20.0%)	0 (0%)	0 (0%)	1 (20.0%)
Unknown	0 (0%)	1 (20.0%)	0 (0%)	0 (0%)	1 (20.0%)
**22q11.2 deletion**					
Yes	0 (0%)	0 (0%)	0 (0%)	0 (0%)	0 (0%)
No	2 (100%)	4 (80.0%)	1 (100%)	3 (100%)	4 (80.0%)
Unknown	0 (0%)	1 (20.0%)	0 (0%)	0 (0%)	1 (20.0%)
**Cardiac operation during first admission**					
Yes	2 (100%)	4 (80.0%)	1 (100%)	3 (100%)	4 (80.0%)
No	0 (0%)	1 (20.0%)	0 (0%)	0 (0%)	1 (20.0%)

© 2024 Gaynor JW et al. *JAMA Network Open.*

eTable 13: Characteristics of Participants Who Died on Study by Cardiac Diagnosis and Treatment

Variables	HLHS Progesterone (N=2)	HLHS Placebo (N=5)	TGA Progesterone (N=1)	Overall Progesterone (N=3)	Overall Placebo (N=5)
**ECMO before 18 months [a]**					
Yes	1 (50.0%)	2 (40.0%)	1 (100%)	2 (66.7%)	2 (40.0%)
No	1 (50.0%)	2 (40.0%)	0 (0%)	1 (33.3%)	2 (40.0%)
**Number of Doses Taken[b]**					
Median	36	144	154	57	144
[Min, Max]	[15, 57]	[132, 152]	[154, 154]	[15, 154]	[132152]
**Percentage of doses missed [b]**					
Median	54.6	0.0	3.1	20.8	0.0
[Min, Max]	[20.8, 88.4]	[0.0, 19.0]	[3.1, 3.1]	[3.1, 88.4]	[0.0, 19.0]
**Birth Weight (kg)[c]**					
Median	2.29	2.50	3.09	2.93	2.60
[Min, Max]	[1.65, 2.93]	[1.21, 3.28]	[3.09]	[1.65, 3.09]	[1.21, 3.28]
**Birth Weight (Z-scores)[c]**					
Median	-0.53	-0.53	-0.54	-0.54	-0.53
[Min, Max]	[-1.08, 0.03]	[-1.22, -0.14]	[-0.54, -0.54]	[-1.08, 0.03]	[-1.22, -0.14]
**Placental Weight (Z-scores)[d]**					
Median	-0.23	-0.72	-2.54	-1.38	-0.72
[Min, Max]	[-0.23, -0.23]	[-2.30, -0.38]	[-2.54, -2.54]	[-2.54, -0.23]	[-2.30, -0.38]
**Impaired MFE**					
Yes	2 (100%)	1 (20.0%)	1 (100%)	3 (100%)	1 (20.0%)
No	0 (0%)	4 (80.0%)	0 (0%)	0 (0%)	4 (80.0%)

[a] Unit of measurement N (%) unless otherwise indicated.

[b]Two individuals both assigned placebo were missing adherence data (percent and number of doses taken).

© 2024 Gaynor JW et al. *JAMA Network Open.*

eTable 13: Characteristics of Participants Who Died on Study by Cardiac Diagnosis and Treatment

Variables	HLHS Progesterone (N=2)	HLHS Placebo (N=5)	TGA Progesterone (N=1)	Overall Progesterone (N=3)	Overall Placebo (N=5)

[c]One individual assigned to placebo was missing birthweight data.

[d]Two individuals, one assigned to placebo and one assigned to progesterone, are missing placental weight data.

© 2024 Gaynor JW et al. *JAMA Network Open.*

Supplemental Figure

eFigure 1: Cumulative Mortality

Cumulative mortality among all participants born on-study (left) and among those with an HLHS diagnosis (right panel). Follow-up ends at 18 months. All but one death, (a TGA subject allocated to progesterone), occurred in those with an HLHS diagnosis.

Figure S2: Common Maternal Adverse Events

Most common adverse events among mothers on Progesterone (right) and mothers on placebo (left). Adverse events were restricted to at least 5% prevalence among the population and further restricted to exclude those that were not related to either Progesterone or the Placebo.

eFigure 3: Common Infant Adverse Events

Most common adverse events among infants stratified by Progesterone (right) and Placebo (left). Adverse events were restricted to at least 5% prevalence among the population and further restricted to exclude those that were not related to either Progesterone or the Placebo.

© 2024 Gaynor JW et al. *JAMA Network Open.*

eFigure 1:

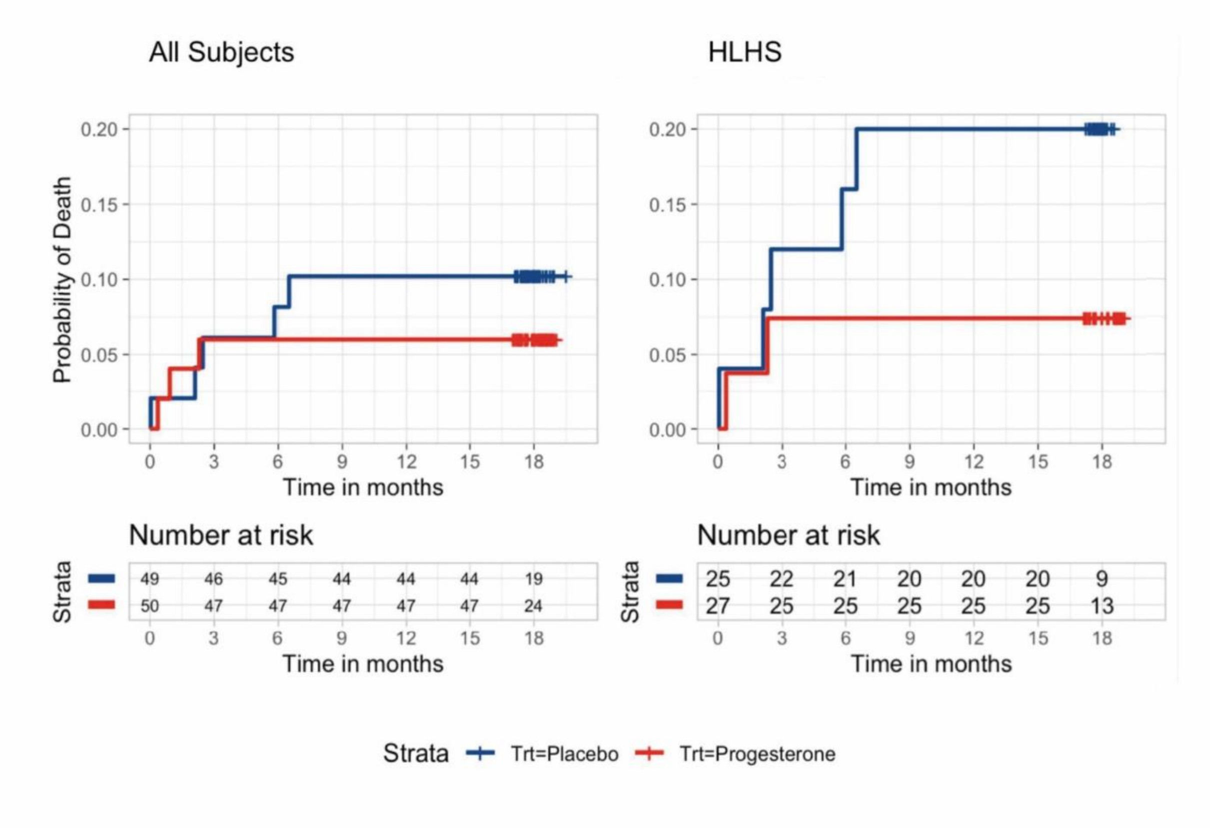

© 2024 Gaynor JW et al. *JAMA Network Open.*

Figure S2:

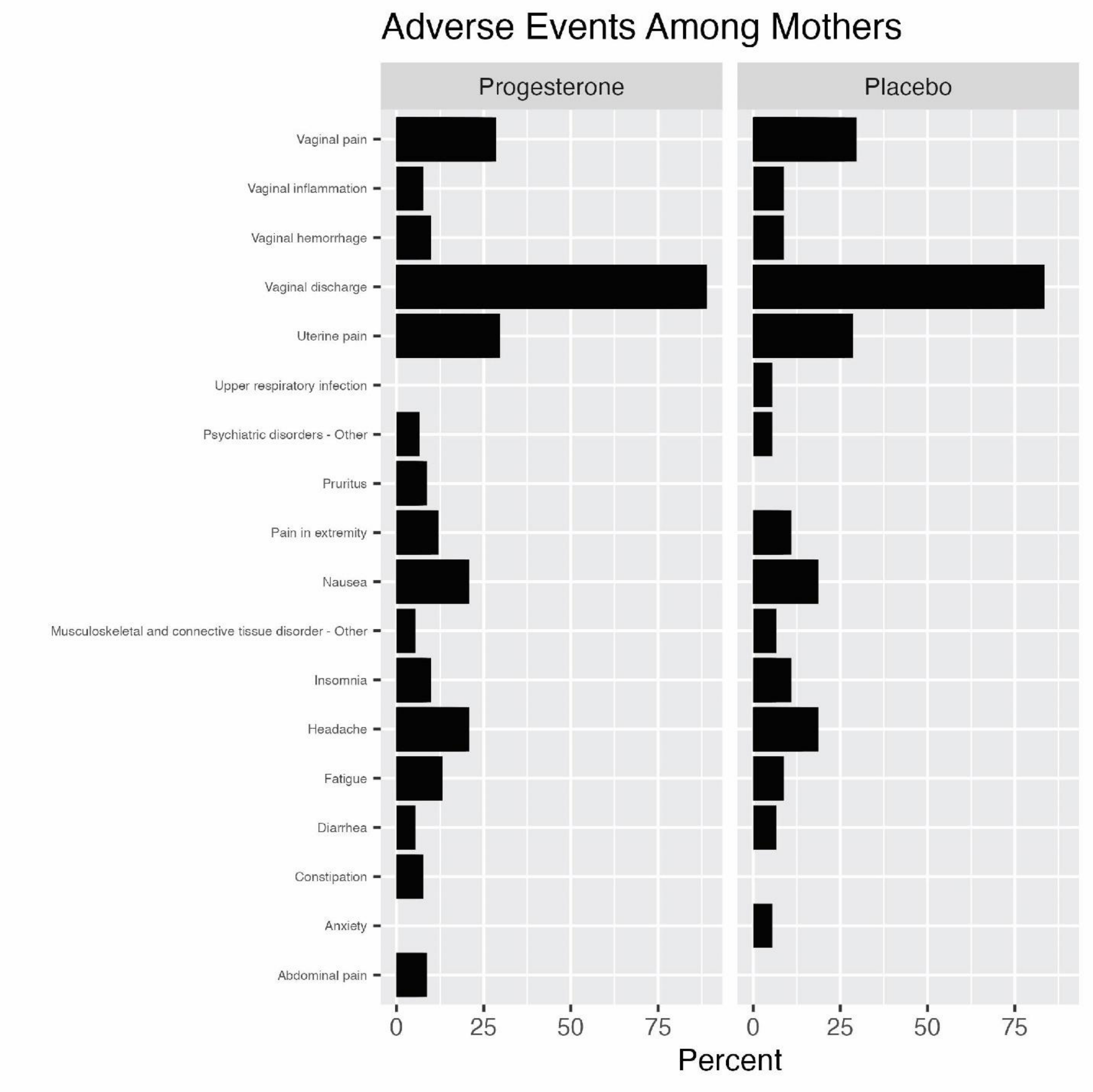

© 2024 Gaynor JW et al. *JAMA Network Open.*

Figure S3:

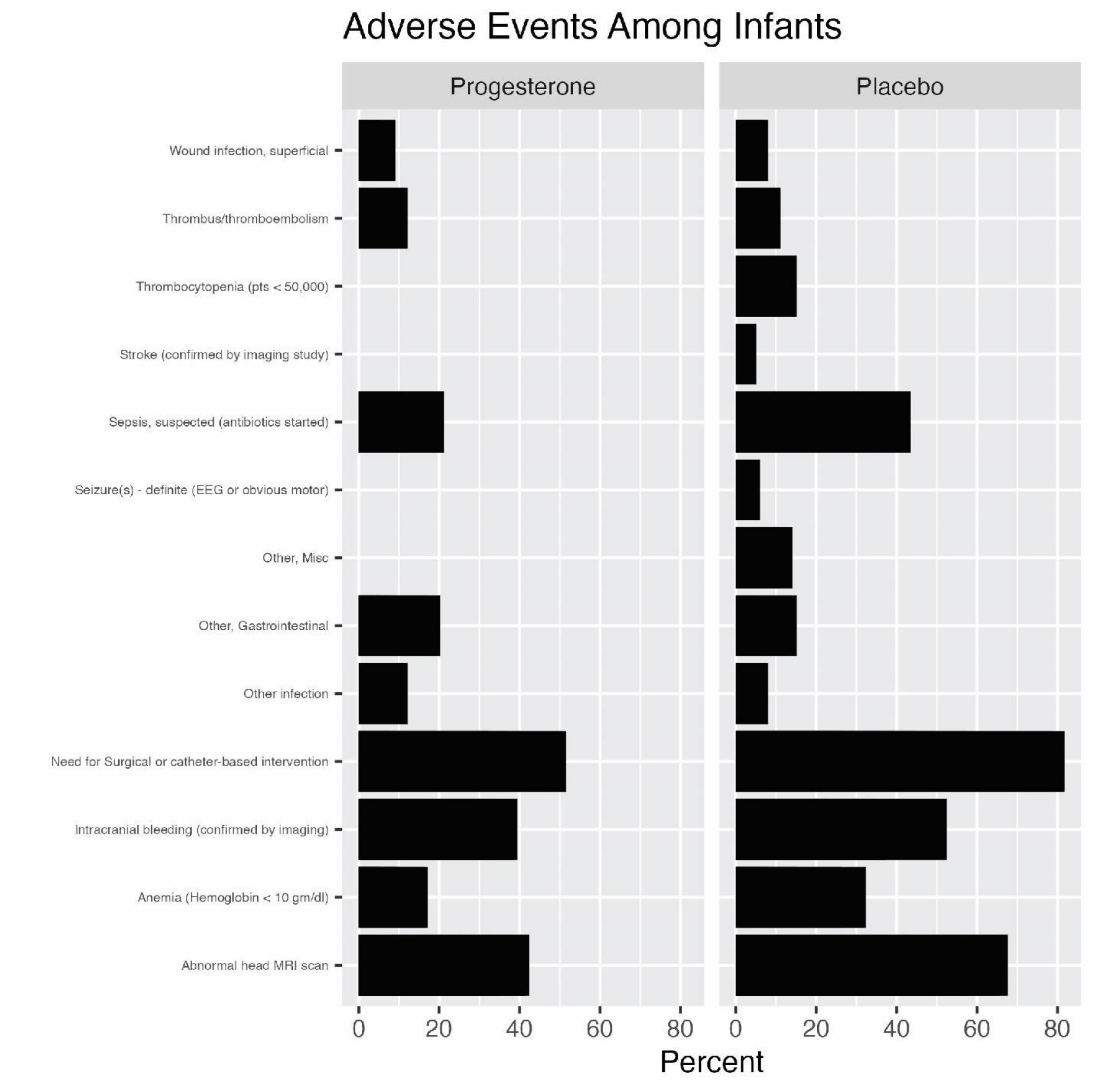

© 2024 Gaynor JW et al. *JAMA Network Open.*
