# Supplementary material for: Progesterone for Neurodevelopment in Fetuses With Congenital Heart Defects: A Randomized Clinical Trial
Source: JAMA Netw Open. 2024 May 28;7(5):e2412291. doi: 10.1001/jamanetworkopen.2024.12291 (PMC11134212; doi:10.1001/jamanetworkopen.2024.12291)
Supplement: Supplement 2. — Trial Protocol and Statistical Analysis Plan [file jamanetwopen-e2412291-s002.pdf]

1

Title: **Randomized Trial of Maternal Progesterone Therapy to Improve Neurodevelopmental Outcomes in Infants with Congenital Heart Disease**

Short Title Maternal Progesterone Study

Drug or Device Name(s): Vaginal natural progesterone

FDA IND (if applicable)XXX-XX

Regulatory Sponsor:

eIRB Number

Protocol Date: 4/3/14

Amendment 1 Date: 3/19/15

Amendment 2 Date: 12/13/2016

Amendment 3 Date: 3/29/2018

Amendment 4 Date: 10/26/2020

Amendment 5 Date: 2/9/2021

Amendment 6 Date: 5/6/2021

Amendment 7 Date: 6/6/2022

Study Principal Investigator

J. William Gaynor

Office Address
Philadelphia, PA 19104
Phone 215-590-2708
email: Gaynor@email.chop.edu

Site Principal Investigator
The Children's Hospital of Philadelphia
J. William Gaynor

Office Address
Division of Cardiac Surgery, Suite 12NW10
34th St. and Civic Center Blvd.
Philadelphia, PA 19104
Phone 215-590-2708
email: Gaynor@email.chop.edu

2

TABLE OF CONTENTS

Table of Contents ii
Abbreviations and Definitions of Terms v
Abstract vii
Protocol Synopsis ix
Table 1: Schedule of Study Procedures xiii
1.1 INTRODUCTION 1
1.2 NAME AND DESCRIPTION OF INVESTIGATIONAL PRODUCT OR INTERVENTION 1
1.3 FINDINGS FROM NON-CLINICAL AND CLINICAL STUDIES 2
1.4 SELECTION OF DRUGS AND DOSAGES 6
1.5 RELEVANT LITERATURE AND DATA 7
1.6 COMPLIANCE STATEMENT 14
2 STUDY OBJECTIVES 14
2.1 PRIMARY OBJECTIVE (OR AIM) 14
2.2 SECONDARY OBJECTIVES (OR AIMS) 14
3 INVESTIGATIONAL PLAN 15
3.1 GENERAL SCHEMA OF STUDY DESIGN 15
3.1.1 Screening Phase 15
3.1.2 In Utero Treatment Phase 15
3.1.3 Post-Natal Phase 15
3.1.4 Neurodevelopmental Evaluation 16
3.2 ALLOCATION TO TREATMENT GROUPS AND BLINDING 16
3.2.1 Randomization 16
3.2.2 Blinding 16
3.2.3 Unblinding 16
3.3 STUDY DURATION, ENROLLMENT AND NUMBER OF SITES 16
3.3.1 Duration of Study Participation 16
3.3.2 Total Number of Study Sites/Total Number of Subjects Projected 17
3.4 STUDY POPULATION 17
3.4.1 Inclusion Criteria 17
3.4.2 Exclusion Criteria 17
4 STUDY PROCEDURES 17
4.1 SCREENING VISIT 17
4.2 IN UTERO TREATMENT PHASE 18
4.2.1 Prenatal Visit 1 18
4.2.2 Subsequent Prenatal Visits (every 2 weeks until 36 weeks GA, then weekly) 18
4.2.3 Follow-up Fetal Brain MRI at 34-36 Weeks GA 18
4.2.4 Delivery 18
4.2.5 Post-Natal Phase 18
4.2.6 Post-Natal Hospitalization 19
4.2.7 Subsequent Hospitalization or Outpatient visit 18
4.2.8 Neurodevelopmental Evaluation 19
4.2.9 Neurodevelopmental Evaluation and End-of-Study 19
4.2.10 Subject's Home 19
4.3 CONCOMITANT MEDICATION 20

4.4 Rescue Medication Administration 20
4.5 Subject Completion/Withdrawal 20
4.5.1 Early Termination Study Visit 20

5 STUDY EVALUATIONS AND MEASUREMENTS 20

5.1 Screening and Monitoring Evaluations and Measurements 20
5.1.1 Pre-Natal Medical Record Review 20
5.1.2 Post-Natal Medical Record Review 20
5.1.3 Physical Examination 20
5.1.4 Vital Signs 21
5.1.5 Laboratory Evaluations 21
5.1.6 Other Evaluations, Measures 22
5.2 Efficacy Evaluations 24
5.2.1 Obstetric Ultrasound 24
5.2.2 Fetal Echocardiography 25
5.2.3 Fetal Cerebral Blood Flow 26
5.2.4 Fetal Brain MRI 27
5.2.5 Post-Natal Brain MRI 27
5.2.6 Neurodevelopmental Evaluation 28
5.3 Pharmacokinetic Evaluation 29
5.4 Safety Evaluation 29
6.1 Primary Endpoint 29
6.2 Secondary Endpoints 29
6.3 Statistical Methods 29
6.3.1 Baseline Data 29
6.3.2 Efficacy Analysis 30
6.3.3 Safety Analysis 30
6.4 Sample Size and Power 30
6.5 Interim Analysis 32

7 STUDY MEDICATION (STUDY DEVICE OR OTHER STUDY INTERVENTION) 32

7.1 Description 32
Packaging 32
7.1.2 Labeling 33
7.1.3 Dosing 33
7.1.4 Treatment Compliance and Adherence 33
7.1.5 Drug Accountability 33

8 SAFETY MANAGEMENT 33

8.1 Clinical Adverse Events 33
8.2 Adverse Event Reporting 33
8.3 Definition of an Adverse Event 34
8.4 Definition of a Serious Adverse Event (SAE) 34
8.4.1 Relationship of SAE to Study Drug 36
8.5 IRB Notification of SAEs and Other Unanticipated Problems 36
8.5.1 Follow-up report 36
8.6 Investigator Reporting of a Serious Adverse Event to Sponsor 36
8.7 Medical Emergencies 36

9 STUDY ADMINISTRATION 37

9.1 Treatment Assignment Methods 37

9.1.1 Randomization *37*
9.1.3 Unblinding *37*
9.2 DATA COLLECTION AND MANAGEMENT 37
9.3 CONFIDENTIALITY 38
9.4 REGULATORY AND ETHICAL CONSIDERATIONS 38
9.4.1 Data and Safety Monitoring Plan *38*
9.4.2 Risk Assessment *39*
9.4.3 Potential Benefits of Trial Participation *42*
9.4.4 Risk-Benefit Assessment *43*
9.5 RECRUITMENT STRATEGY (OR CASE ASCERTAINMENT) 43
9.6 INFORMED CONSENT/ASSENT AND HIPAA AUTHORIZATION 44
9.7 PAYMENT TO SUBJECTS/FAMILIES 44
9.7.1 Reimbursement for travel, parking and meals *44*
9.7.2 Payments to parent for time and inconvenience (i.e. compensation) *44*
9.7.3 Gifts *44*
10 PUBLICATION 45
11 REFERENCES 46
Appendix 53

116 **ABBREVIATIONS AND DEFINITIONS OF TERMS**

AC	Abdominal circumference
AE	Adverse event
APOE	Apo-lipoprotein E
ART	Assisted reproductive technology
ASO	Arterial switch operation
Bayley-III	Bayley Scales of Infant and Toddler Development-III
BCAS	Boston Circulatory Arrest Study
BDNF	Brain derived neurotrophic factor
BMI	Body-mass index
BPD	Biparietal diameter
CAG	Center for Applied Genomics
CPB	Cardiopulmonary bypass
CHD	Congenital heart disease
CHOP	Children’s Hospital of Philadelphia
CICU	Cardiac Intensive Care Unit
CNS	Central nervous system
CNV	Copy Number Variation
CRH	Corticotrophin releasing hormone
CTCAE	Common Terminology Criteria for Adverse Events
CVO	Combined ventricular output
DMS	Data management system
DNA	Deoxyribonucleic acid
DSMB	Data Safety Monitoring Board
DV	Ductus Venosus
EFW	Estimated fetal weight
FDA	Food and Drug Administration
FL	Femoral length
FSIQ	Full-Scale Intelligence Quotient
fTMS	Fetal Total Maturation Score
GA	Gestational age
HC	Head circumference
HL	Humeral length
HLHS	Hypoplastic left heart syndrome
IRB	Institutional review Board
ITSEA	Infant-Toddler Social and Emotional Assessment
ITT	Intention-to-treat
IVC	Inferior vena cava
IVH	Intra-ventricular hemorrhage
LVO	Left ventricular output
MCA	Middle cerebral artery
M-CHAT	Modified Checklist for Autism in Toddlers
MDI	Mental Developmental Index

MPA	Medroxyprogesterone acetate
MRI	Magnetic resonance imaging
NEC	Necrotizing enterocolitis
NICU	Neonatal Intensive Care Unit
PCR	Polymerase chain reaction
PDD	Pervasive Developmental Disorder
PDI	Psychomotor Development Index
PI	Pulsatility Index
PIQ	Performance Intelligence Quotient
PVL	Periventricular leucomalacia
RCT	Randomized clinical trial
RDS	Respiratory distress syndrome
RI	Resistive index
ROI	Region of interest
RR	Relative risk
RVO	Right ventricular output
SAE	Serious adverse event
SAR	Specific absorption ratio
SD	Systolic to diastolic ratio
SES	Socioeconomic status
SNP	Single-nucleotide polymorphism
SVC	Superior vena cava
TBI	Traumatic brain injury
TGA	Transposition of the great arteries
TMS	Total maturation score
UA	Umbilical artery
UtA	Maternal uterine arteries
VMWM	Volume of myelinated white matter
17-OHP-C	17 α-hydroxyprogesterone caproate

ABSTRACT

Context:

Neurodevelopmental disability is now recognized as the most common long-term complication after cardiac surgery in neonates. White matter injury is the most common finding on neuroimaging and pathological studies in this population. Delayed brain maturation during fetal development, especially involving white matter, has been identified as an important mechanism underlying the risk of neurodevelopmental disability. Progesterone has been shown to be neuroprotective by accelerating white matter development and improving myelination.

Objectives:

Develop preliminary evidence to support a multi-institutional study to determine whether, in women carrying fetuses (maternal-fetal dyad) with congenital heart defects (CHD), prophylactic vaginal natural progesterone therapy is neuroprotective, and compared to placebo:

1. improves neurodevelopmental outcomes at 18 months,
2. improves fetal brain growth and maturation,
3. increases myelination during fetal brain development,
4. reduces pre-operative brain white matter injury, and
5. reduces post-operative white matter injury.

Study Design:

Phase II single-center placebo-controlled randomized therapeutic exploratory trial.

Setting/Participants:

Setting:

The study will be performed in the Center for Fetal Diagnosis and Treatment/Fetal Heart Program, as well as inpatient and outpatient units at CHOP.

Inclusion Criteria:

Mother carrying a fetus with CHD (maternal-fetal dyad) requiring surgery with cardiopulmonary bypass (CPB) prior to 44 weeks corrected gestational age (GA) identified prior to 28 weeks GA.

Exclusion Criteria:

1) Major genetic or extra-cardiac anomaly other than 22q11 deletion
2) Language other than English spoken in the home
3) Known sensitivity or listed contraindication to progesterone (known allergy or hypersensitivity to progesterone, severe hepatic dysfunction, undiagnosed vaginal bleeding, mammary or genital tract carcinoma, thrombophlebitis, thromboembolic disorders, cerebral hemorrhage, porphyria)
4) Prescription or ingestion of medications known to interact with progesterone (e.g. Bromocriptine, Rifamycin, Ketoconazole or Cyclosporin)
5) Maternal use of progesterone within 30 days of enrollment
6) History of preterm birth or short cervix (defined as cervical length ≤ 25 mm at 18-24 weeks GA necessitating progesterone therapy
7) Multiple gestation
8) Maternal contraindication for magnetic resonance imaging (MRI)

9) Subjects with a known history of non-compliance with medical therapy.

Study Interventions and Measures:

Study drug:

Vaginal natural progesterone, 90 mg BID as a vaginal gel from 24–39 weeks gestation, compared to placebo.

Main study outcome measures:

1. Neurodevelopmental evaluation at 18 months of age:
    - Bayley Scales of Infant and Toddler Development-III
    - Modified Checklist for Autism in Toddlers (M-CHAT)
    - Infant-Toddler Social and Emotional Assessment (ITSEA).
    - Neurological examination
2. Fetal brain MRI at 24-28 weeks GA prior to randomization.
3. Fetal brain MRI at 34-36 weeks GA.
4. Routine prenatal visits (every 2 weeks until 36 weeks, then weekly).
5. Obstetric ultrasound (every 4 weeks).
6. Routine fetal echocardiography (every 4 weeks).
7. Maternal blood tests: progesterone, genetic testing.
8. Paternal blood tests: genetic testing.
9. Cord blood tests: progesterone.
10. Infant blood testing: genetic testing.
11. Early post-natal (preoperative) brain MRI (day of surgery).
12. Post-operative brain MRI (within 10 days of surgery).

PROTOCOL SYNOPSIS

**Study Title**	**Randomized Trial of Maternal Progesterone Therapy to Improve Neurodevelopmental Outcomes in Infants with Congenital Heart Disease**
**Funder**	Hospital and Departmental funds
**Clinical Phase**	Phase II
**Study Rationale**	In the United States, approximately 1 in every 100 newborns is diagnosed with congenital heart disease (CHD). Many of these newborns (25%-35%) will require either corrective or palliative open-heart surgery. As recently as the 1960's, only 20% of newborns with critical CHD survived to adulthood. Today, thanks to better diagnostic technologies and methods (including prenatal diagnosis), advances in surgery, and improved postoperative care, early survival is over 90%. However, with improved early outcomes has come the sobering recognition that there is an ongoing risk of late mortality, as well as significant morbidity for these children. In particular, neurodevelopmental disability is now recognized as the most common complication of critical CHD (i.e. those patients requiring cardiac surgery in infancy) and has the most negative impact on quality of life, academic performance, and opportunity for independence as an adult.<br>The altered fetal hemodynamics secondary to CHD lead to decreased blood flow and/or oxygen delivery to the fetal brain. In turn, this impairment in blood flow and oxygenation results in impaired brain growth and altered structural and cellular maturation, particularly of the white matter. Fetal MRI studies have shown that during the third trimester, normally a time of rapid brain growth and development, brains of infants with CHD fail to grow at the same rate as the brains of fetuses without CHD. This growth delay results in microcephaly, immature cellular elements of the white matter and decreased cortical folding at birth. It has been demonstrated that brain immaturity at birth is a primary major risk factor underlying the hypoxic-ischemic white matter brain injury and subsequent neurodevelopmental disability seen in over 50% of infants following surgery for CHD. In addition, there is increasing evidence in the CHD population that even late pre-term birth (prior to 39 weeks GA) is associated with increased mortality, increased peri-operative morbidity, and worse neurodevelopmental outcomes.<br>Progesterone is an essential hormone in the occurrence and maintenance of pregnancy. Progesterone administration has also been shown to be neuroprotective in a variety of clinical situations, including traumatic brain injury (TBI). Sex steroid hormones, including progesterone, are critically involved in axonal myelination, forming the basis of white matter connectivity in the central nervous system (CNS). Progesterone and its metabolites

	not only promote the viability and regeneration of neurons, but also act on myelinating glial cell oligodendrocytes in the CNS and play an important role in the formation of myelin sheaths. Progesterone has also been shown to increase myelination and enhance maturation of immature oligodendrocytes progenitor cells to mature oligodendrocytes, which are more resistant to hypoxic/ischemic injury. Therapeutic administration of progesterone has also been demonstrated to prevent preterm birth. Thus, there are two potential mechanisms by which pre-natal progesterone therapy may improve neurodevelopmental outcomes in neonates with CHD: 1) a direct neuroprotective effect, and 2) decreasing the occurrence of pre-term birth.
**Study Objective(s)**	**Primary**<br>Develop preliminary evidence to support a multi-institutional study to determine whether, in women carrying fetuses (maternal-fetal dyad) with CHD, prophylactic vaginal natural progesterone therapy is neuroprotective, and compared to placebo improves neurodevelopmental outcomes at 18 months of age.<br>**Secondary**<br>Develop preliminary evidence to support a multi-institutional study to determine whether, in women carrying fetuses (maternal-fetal dyad) with CHD, prophylactic vaginal natural progesterone therapy is neuroprotective, and compared to placebo:<br>1. improves fetal brain growth and maturation,<br>2. increases myelination during fetal brain development,<br>3. reduces pre-operative brain white matter injury, and<br>4. reduces post-operative white matter injury.
**Test Article(s)**<br>*(If Applicable)*	Prophylactic vaginal natural progesterone (90 mg as an 8% vaginal gel BID) from 24–39 weeks gestation, compared to placebo.
**Study Design**	Phase II single-center placebo-controlled randomized therapeutic exploratory trial.
**Subject Population**<br>**key criteria for Inclusion and Exclusion:**	**Inclusion Criteria**<br>Mother carrying a fetus with CHD (maternal-fetal dyad) requiring surgery with cardiopulmonary bypass (CPB) prior to 44 weeks corrected gestational age (GA) identified prior to 28 weeks GA.<br>**Exclusion Criteria**<br>1. Major genetic or extra-cardiac anomaly other than 22q11 deletion<br>2. Language other than English spoken in the home<br>3. Known sensitivity or listed contraindication to progesterone (known allergy or hypersensitivity to progesterone, severe hepatic dysfunction, undiagnosed vaginal bleeding, mammary or genital tract carcinoma, thrombophlebitis, thromboembolic disorders, cerebral hemorrhage, porphyria)

	4. Prescription or ingestion of medications known to interact with progesterone (e.g. Bromocriptine, Rifamycin, Ketoconazole or Cyclosporin)<br>5. Maternal use of progesterone within 30 days of enrollment<br>6. History of preterm birth or short cervix (defined as cervical length ≤ 25 mm at 18-24 weeks GA necessitating progesterone therapy<br>7. Multiple gestation<br>8. Maternal contraindication for MRI<br>9. Subjects with a known history of non-compliance with medical therapy.
**Number Of Subjects**	We plan to enroll sufficient patients to have 80 evaluable patients (40 per treatment group) at the 18-month neurodevelopmental testing. Based on known clinical outcomes for this patient population and experience with other studies with similar follow-up plans, we estimate a 5% early mortality and a further 20% attrition from all causes between hospital discharge and the 18-month evaluation. There may also be dropout prior to birth for a variety of reasons: fetal death, intolerance of therapy, withdrawal from study. We do not have accurate data to estimate this loss. We anticipate needing to enroll 110-130 patients to have a sufficient cohort at 18 months (50 per treatment group), with 60 and 52-56 patients, respectively, per treatment group available for the post-natal and post-operative brain MRIs.
**Study Duration**	The study begins during the pregnancy at 24 weeks GA and continues until the baby is 18 months of age.
**Study Phases**<br>**Screening**<br>**Study Treatment**<br>**Follow-Up**	1. Screening<br>2. Enrollment, baseline evaluation, randomization<br>3. Treatment phase (24 to 39 weeks gestational age)<br>4. Evaluation Phase (birth to 18 months of age)
**Efficacy Evaluations**	1. Motor Scale of the Bayley-III at 18 months of age.<br>2. Cognitive and Language Scales of the Bayley-III at 18 months of age.<br>3. M-CHAT and ITSEA at 18 months of age.<br>4. Neurological examination.<br>5. Total maturation score (TMS)<br>6. Global and regional brain volumes<br>7. Volume of myelinated white matter (VMWM)<br>8. Presence of periventricular leucomalacia (PVL) on early post-natal brain MRI (categorical y/n)<br>9. Presence of PVL on early post-operative brain MRI (categorical y/n)<br>10. PVL Volume on early post-natal brain MRI<br>11. PVL Volume on early post-operative brain MRI
**Pharmacokinetic Evaluations**	*(include only if applicable)*
**Safety Evaluations**	The mother and fetus will be seen every 1-2 weeks after

	enrollment and undergo physical examination, obstetric ultrasound. Fetal echocardiography will be performed every 4 weeks and more frequently if clinically indicated. After birth the baby will undergo physical examination, echocardiography, and brain MRI.
**Statistical And Analytic Plan**	The primary analysis will be intent-to-treat (ITT) and will estimate differences between group means and their associated confidence intervals. In parallel we will test the null hypothesis that treatment has no effect on each of three outcomes. Because of its clinical relevance, the study is primarily designed to provide an estimate of possible effect sizes for the Bayley-III Motor Scale at 18 months. Important information will also be provided on effect sizes for the Bayley-III Cognitive and Language Scales at 18 months, M-CHAT, and ITSEA, as well as total maturation score (TMS) and volume of myelinated white matter (VMWM).The analysis will use a two-sided two-sample T-interval (estimation) or T-test (hypothesis testing). The confidence level will be set at 90% and the Type I error at 10%. Normality will be assessed and a transformation, or alternatively a non-parametric test, will be used if needed.
**DATA AND SAFETY MONITORING PLAN**	Data quality management and ongoing assessment of safety will be the responsibility of the PI and multi-disciplinary Steering Committee. An independent Data Safety Monitoring Board (DSMB) will be established.

187 **TABLE 1: SCHEDULE OF STUDY PROCEDURES**

Study Phase	Maternal Interventions and Assessments									Child Assessments			
	Fetal Echocardiography	Obstetric Ultrasound	Fetal Brain MRI	Maternal Blood or Saliva Tests	Paternal Blood or Saliva Tests	Institution of Study Drug	Prenatal Visit (q 2 week until 36 weeks, then weekly)	Termination of Study Drug		Cord Blood Tests	Infant Blood (from existing blood drawing line/routine clinical or separate research study labwork) or Saliva Sample Tests	Brain MRI	Neurodevelopmental Evaluation
Screening and Enrollment<br><br>(24 to 28 weeks GA)	X	X											
Prior to Randomization			X	X	X		X						
Randomization						X							
After Randomization	q 4 weeks	q 4 weeks					X						
35 Weeks GA			X	X									
39 weeks GA								X					

**Birth**				X						X	X		
**Prior to Surgery**<br><br>**(day of surgery)**												X	
**After Surgery**<br><br>**(within 7 days)**												X	
**Subsequent Hospitalization or Outpatient Visit**				X	X						X		
**18 months of Age**				X	X						X		X

1 BACKGROUND INFORMATION AND RATIONALE

1.1 Introduction

In the United States, approximately 1 in every 100 newborns is diagnosed with congenital heart disease (CHD). Many of these newborns (25%-35%) will require either corrective or palliative open-heart surgery. As recently as the 1960's, only 20% of newborns with critical CHD survived to adulthood. Today, thanks to better diagnostic technologies and methods (including prenatal diagnosis), advances in surgery, and improved postoperative care, early survival is over 90%. However, with improved early outcomes has come the sobering recognition that there is an ongoing risk of late mortality as well as significant morbidity for these children. In particular, neurodevelopmental disability is now recognized as the most common complication of critical CHD (i.e. those patients requiring cardiac surgery in infancy) and has the most negative impact on quality of life, academic performance, and opportunity for independence as an adult.

Altered fetal hemodynamics secondary to presence of CHD may cause decreased blood flow and/or oxygen delivery to the fetal brain. In turn, this impairment in blood flow and oxygenation results in impaired brain growth and altered structural and cellular maturation, particularly of the white matter. Studies using fetal brain MRI have shown that during the third trimester, normally a time of rapid brain growth and development, the brains of infants with CHD fail to grow at the same rate as the brains of fetuses without CHD; resulting in microcephaly, immature cellular elements of the white matter and decreased cortical folding at birth. It has been demonstrated that brain immaturity at birth is a primary major risk factor underlying the hypoxic-ischemic white matter brain injury and subsequent neurodevelopmental disability seen in over 50% of infants following for CHD. In addition, there is increasing evidence in the CHD population that even late pre-term birth (prior to 39 weeks GA) is associated with increased mortality, increased peri-operative morbidity, and worse neurodevelopmental outcomes.

Progesterone is an essential hormone in the occurrence and maintenance of pregnancy. Progesterone administration has also been shown to be neuroprotective in a variety of clinical situations, including traumatic brain injury (TBI). Sex steroid hormones, including progesterone, are critically involved in axonal myelination, forming the basis of white matter connectivity in the central nervous system (CNS). Progesterone and its metabolites not only promote the viability and regeneration of neurons, but also act on myelinating glial cell oligodendrocytes in the CNS and play an important role in the formation of myelin sheaths. Progesterone has also been shown to increase myelination and enhance maturation of immature oligodendrocytes progenitor cells to mature oligodendrocytes, which are more resistant to hypoxic/ischemic injury. Therapeutic administration of progesterone has also been demonstrated to prevent preterm birth.

We propose a Phase II single-center placebo-controlled randomized therapeutic exploratory trial to determine whether there is evidence that progesterone, administered pre-natally, improves neurodevelopmental outcomes, improves brain development, and reduces perioperative white matter injury in neonates with CHD undergoing cardiac surgery. The data provided by this therapeutic exploratory study will aid in selection of appropriate outcomes and inform sample size calculations for a future multi-institutional study.

1.2 Name and Description of Investigational Product or Intervention

Natural progesterone (Crinone) *Intravaginal gel:* 90 mg (8% gel) twice daily

1.3 Findings from Non-Clinical and Clinical Studies

1.3.1 Progesterone Pharmacokinetics

Progesterone is produced in significant amounts by the luteal phase ovary and the fetoplacental unit in the reproductive-age woman.[1] It is produced by the adrenal gland in small amounts; but in large amounts in the postovulatory ovary. Progesterone is metabolized principally in the liver by the 5a-reductase pathway resulting in pregnanediol and pregnanetriol; and by conjugation with glucuronide.[1] Progesterone's precursors and metabolites do not have significant progestational activity. Progesterone circulates in the serum bound to cortisol-binding globulin, bound to albumin, or as free hormone.[1]

Oral progesterone produces poorly sustained plasma progesterone concentrations. There is considerable first-pass prehepatic and hepatic metabolism.[2, 3] The vaginal route for systemic drug administration is advantageous for administration of drugs such as progesterone, which exert their activity on the uterus and there is evidence of direct vaginal to uterus transport of progesterone. [2, 4] Uterine tissue concentrations of progesterone are significantly higher after vaginal administration than after systemic administration, despite higher blood levels with systemic administration.[3, 4] In a randomized trial, women undergoing hysterectomy received either vaginal or IM progesterone. Ratios of endometrial to serum progesterone concentrations were higher after vaginal administration of progesterone than after IM injections.[3] The lower blood levels after vaginal administration have been associated with a lower prevalence of systemic side effects.[3, 5]

Although vaginal progesterone is commonly used in conjunction with assisted reproductive technologies (ART) and to prevent preterm birth, there is very little data concerning placental levels, transplacental transfer or fetal blood levels. A study using ex vivo perfusion of placentas from term pregnancies showed that 17-α-Hydroxyprogesterone caproate is metabolized by placental lobules. Both the parent compound and the metabolites were transferred to the fetal circuit.[6]

1.3.2 Neuroprotective Effects of Progesterone

Progesterone is classified as a neurosteroid. The enzymes necessary for its synthesis are present in the brain and the progesterone receptor is widely distributed in the forebrain, limbic system and hypothalamus early in development.[7] The potential neuroprotective effects of progesterone were discovered because of sex differences in recovery from experimental brain injury. Progesterone is neuroprotective in animal models of stroke. Administration of progesterone prior to middle cerebral artery occlusion decreases infarct size.[8] Post-ischemic administration has also been shown to reduce injury. Progesterone reduces cerebral edema after experimental TBI.[9] Progesterone also has a positive effect after spinal cord contusion.[10] Following peripheral nerve injury, progesterone administration has been shown to induce re-myelination.[11] There is growing evidence, including recently published clinical trials, that progesterone and its metabolite allopregnanolone exert neuroprotective effects on the injured CNS in humans.[12] These positive effects include not only decreased mortality, but also reduced morbidity and improved functional outcomes.[13-15]

A recent Cochrane review evaluated all randomized controlled trials looking at the effects of progesterone in people with TBI.[16] The review focused on randomized controlled trials in which, in addition to standard treatment, the experimental group received progesterone and the control group received non-active treatment (placebo) or no additional treatment. The authors identified three studies with a total of 315 subjects. There was evidence of a beneficial effect of progesterone on mortality and disability after TBI. Subjects in the progesterone group had a risk of death around 40% lower than those in the

control group. The results of this review indicate that progesterone may reduce mortality and disability compared to the control group with no additional adverse events.

The effectiveness of progesterone as a neuroprotective agent is probably related to its role in brain development for both males and females. Fetuses of both sexes are exposed to high levels of maternal progesterone during almost the entire period of gestation.[14] Many of the processes involved in CNS repair after brain injury are thought to recapitulate normal brain development.[17] Progesterone is synthesized by oligodendrocytes and in excitatory neurons in the brains of both males and females in roughly equal amounts.[18] Progesterone and allopregnanolone reduce excessive excitotoxicity and inflammation while maintaining normal levels of cell proliferation and apoptosis in the developing brain.[19] Numerous potential mechanisms likely underlie the neuroprotective effects of progesterone and allopregnanolone.[9] These include regulation of neurotropin production; increased expression of brain derived neurotrophic factor (BDNF); activation of specific signaling pathways including the cAMP/PKA, MAPK (ERK1/2), and the PI-3K/Akt pathways, previously implicated in mediating neuroprotective effects; and regulation of the $GABA_A$ receptor.[20-24]

The prenatal period is a critical time for brain development and early organization of neural circuits.[25, 26] White matter development and myelination is essential for the conduction of brain signals. Communication between brain regions is established through white matter tracts consisting of myelinated axons.[26] Disruption of brain myelination processes during development can lead to lasting neurological, cognitive and motor effects. In the newborn with CHD, white matter injury is the most common lesion identified by neuroimaging studies, and is present in up to 50% of patients early after cardiac surgery.[27, 28] The primary mechanism of white matter injury is thought to be hypoxic/ischemic injury to vulnerable oligodendrocyte precursors. Thus, the protective effect of progesterone on oligodendrocytes, their vulnerable progenitors and myelination is of special interest.

Progesterone is involved in the regulation of oligodendrocyte precursor proliferation and maturation.[29] There is evidence that progesterone promotes myelination in the CNS, by stimulating the proliferation and maturation of oligodendrocyte precursors into myelinating oligodendrocytes.[30] Progesterone has also been shown to have positive effects on myelination in the developing CNS, likely by enhancement of the maturation of oligodendrocytes precursors into myelinating oligodendrocytes. Progesterone has pro-myelinating effects in cultures of glial cells and brain slice cultures.[30] Overall, progesterone increases the number of oligodendrocytes, the formation of myelin sheaths, and the synthesis of myelin proteins.[26, 31] Progesterone has also been shown to increase myelin basic protein in organotypic slice cultures of rat cerebellum.[32] Further evidence of the critical role of progesterone in fetal white matter development is provided by a recent study of chronic inhibition of allopregnanolone synthesis in the fetal brain.[33] Chronic administration of finasteride during late gestation was used to reduce allopregnanolone concentrations in the fetal guinea pig brain. Finasteride treatment was associated with significantly reduced myelination in the subcortical white matter.[33] In addition, neuroactive steroids are important in protecting the fetal brain against hypoxia.[19] Progesterone and its metabolites reduce excitotoxic cell death and may stimulate repair processes.[34] The fetal brain responds to acute hypoxia by increasing allopregnanolone synthesis, which may ameliorate white matter injury. The role of exogenous progesterone administration in protecting against hypoxia induced white matter injury is not known.

The neuroprotective effects are highly dependent on the type of progesterone administered. There are important differences in the neurobiology of progesterone and the synthetic medroxyprogesterone acetate (MPA), which is the most commonly used progestin

in hormone therapy regimens.[9] MPA is a synthetic progestin derived from 17α-hydroxyprogesterone. Both natural progesterone and MPA are used to prevent preterm birth. Progesterone and MPA are equally effective at reducing the uterotrophic effects of unopposed estrogen treatment, but their effects on the brain are not identical. It has become clear that while progesterone is neuroprotective, MPA is not.[9] The difference in neuroprotective potential may be explained by differential effects on BDNF expression; progesterone increases BNDF levels, while MPA has no effect.[35]

The post-natal effects of progesterone therapy during fetal life were evaluated in a multicenter randomized controlled trial of 17 alpha-hydroxyprogesterone caproate (17-OHP-C) vs. placebo to prevent preterm birth. Surviving children underwent physical examination and developmental screening at 48 months of age. Progesterone therapy was not associated with any adverse physical or neurodevelopmental sequelae.[36] The lack of improvement in neurodevelopmental outcomes is not surprising given the lack of neuroprotective effect of 17-OHP-C. But no adverse effects of therapy were identified.

The effects of post-natal progesterone and estradiol supplementation were evaluated in a randomized clinical trial of extremely low birth weight infants (n=30).[37] Therapy was initiated at a median gestational age of 26.6 weeks (corresponding to the third trimester of fetal development) and continued for 6 weeks. There were no early adverse effects of the therapy. The treatment group showed a trend towards less lung disease and better bone mineralization. Neurodevelopmental follow-up was performed at 15 months of age using the Bayley Scales of Infant Development-II.[38] Median Psychomotor Developmental Index (PDI) scores were 101 for the treatment group (n=11) and 71 for the control group (n=10). Median Mental Developmental Index scores were 89 and 93 respectively. At a 5 year evaluation, there was a trend towards larger head circumference in the treatment group.[39] Every additional day of therapy was associated with a reduced incidence of cerebral palsy and spasticity. The number of patients is small, but the results suggest a potential benefit of progesterone therapy.

1.3.3 Progesterone and Preterm Birth

In humans, progesterone is necessary for fertilization and maintenance of pregnancy (by maintaining myometrial relaxation).[40, 41] Progesterone is initially produced by the corpus luteum and from 7–8 weeks of gestation increasingly by the placenta. At term, in a singleton pregnancy, the placenta produces approximately 250–300 mg progesterone per day.[40] Inhibition of the production/effect of progesterone will induce parturition.[40] Progesterone seems to have three different potential modes of action which maintain pregnancy: 1) functional progesterone withdrawal has been suggested to be essential in humans, i.e. changes in receptor levels or metabolism of progesterone[40]; 2) maternal levels of placental corticotrophin releasing hormone (CRH) increase with gestational age and peak at delivery. The rise in placental CRH levels is more rapid in women who deliver preterm compared to women who deliver at term. Progesterone has an inhibitory effect on placental CRH production which may prevent preterm birth; 3) labor is preceded by maturation of the cervix through up-regulation of interleukins and increased prostaglandin synthesis. Progesterone attenuates these effects, possibly by an anti-inflammatory effect.[40]

A characteristic of human pregnancy is that circulating progesterone levels do not fall with the onset of labor. The mechanisms leading to functional withdrawal of progesterone effect likely involves the progesterone receptor.[41] There are several variants arising from transcription of the single progesterone receptor gene at alternative start sites. Progesterone receptor B, the most common transcript, mediates many of the actions of progesterone. However, there are shorter transcripts, including progesterone receptors A

and C. The shorter variant receptors (A and C) lack an N-terminal–activating domain, and can function as dominant repressors of the function of the progesterone receptor B.[41] The proportions of progesterone receptors A, B, and C change with the onset of labor in a way that could constitute a mechanism of progesterone withdrawal.

There is increasing evidence that administration of progesterone to women at increased risk of preterm birth (those with a short cervix and possibly those with a history of preterm birth) is an effective therapy to delay onset of parturition.[42] A Cochrane review of 36 randomized controlled trials involving 8,523 women found that progesterone therapy had benefits which include: prolonging the pregnancy, decreased neonatal mortality, decreased need for assisted ventilation and decreased risk of necrotizing enterocolitis.[43] A recent systematic review concluded that in women with a singleton pregnancy and previous preterm delivery, progesterone reduces the rates of preterm delivery before 32 weeks, perinatal death, as well as respiratory distress syndrome and necrotizing enterocolitis in the newborn.[40] A systematic review of interventions to prevent small-for-gestational age fetuses and to prevent perinatal mortality found that progesterone therapy to prevent preterm birth was one of the most effective therapies.[44]

The optimal progesterone preparation and dosage has not been fully determined. In a recent review, the authors concluded that women with a history of prior spontaneous preterm birth should be offered progesterone therapy.[45] Progesterone supplementation in women with a short cervix could be considered. Based on existing evidence, a dose of 250 mg of 17 α-hydroxyprogesterone caproate (17-OHP-C) given weekly starting between 16/0 and 20/6 weeks of gestation and continued until 34 to 36 weeks gestation would be reasonable. The use of 100 to 200 mg intravaginal progesterone nightly or 90 mg intravaginal progesterone gel nightly could be considered.[45] A recent study evaluated treatment with vaginal progesterone gel of up to 400 mg per day in twin pregnancies.[46] No benefit of 400 mg compared to 200 mg was found. Importantly, no serious systemic adverse effects were encountered and local tolerance was good in all but one woman. The rate of gestational problems was not higher with the larger dose, except for a small increase in the incidence of generalized pruritus and a dose-dependent, non-significant, trend towards a higher incidence of intrahepatic cholestasis.

The effects of progesterone on perinatal outcomes and the neonate are of special interest. A recent meta-analysis attempted to quantify the effect of progesterone therapy for the prevention of preterm birth on perinatal outcomes.[42] Sixteen studies (singletons, n=7; twins, n=7; triplets, n=2) were included. Outcomes evaluated were neonatal and perinatal death, respiratory distress syndrome (RDS), retinopathy, necrotizing enterocolitis (NEC), intraventricular hemorrhage (IVH) Grade 3–4, sepsis, admission to the neonatal intensive care unit (NICU) and composite adverse outcome. For singleton pregnancies, progesterone reduced the rates of neonatal death (risk ratio (RR) 0.487 (95% CI, 0.290–0.818)), RDS (RR 0.677 (95% CI, 0.490–0.935)), NICU admission (RR 0.410 (95% CI, 0.204–0.823)) and composite adverse outcome (RR 0.576 (95% CI, 0.373–0.891)). No favorable effect was observed in twin gestations. Progesterone was associated with increased rates of perinatal death (RR 1.551 (95% CI, 1.014–2.372)), RDS (RR 1.218 (95% CI, 1.038–1.428)), and composite adverse outcome (RR 1.211 (95% CI, 1.029–1.425)) in twin gestations. No significant effect was observed in triplet pregnancies. The authors concluded that progesterone administration in singleton pregnancies at risk for preterm birth improves perinatal outcomes, but may actually have adverse effects in multiple pregnancies.[42] Other reviews have also found that there do not appear to be any significant maternal or fetal risks associated with progestin therapy.[45] Longer term outcomes have also been evaluated.[36] Surviving children from a trial of 17-OHP-C vs. placebo to prevent preterm birth

underwent physical examination and developmental screening at 48 months of age. Progesterone therapy was not associated with any adverse physical or neurodevelopmental sequelae.[36]

1.3.4 Ongoing Clinical Trial

The OPPTIMUM study is a double blind randomized placebo controlled trial to determine whether progesterone prophylaxis to prevent preterm birth has long term neonatal or infant benefit.[47] Specifically it will study whether, in women with singleton pregnancy and at high risk of preterm labor, prophylactic vaginal natural progesterone, 200 mg daily from 22–34 weeks gestation, compared to placebo, improves obstetric outcome by lengthening pregnancy thus reducing the incidence of preterm delivery (before 34 weeks), improves neonatal outcome by reducing a composite of death and major morbidity, and leads to improved childhood cognitive and neurosensory outcomes at two years of age. Recruitment began in 2009 and is scheduled to close in spring, 2013. As of May 2012, over 800 women had been randomized in 60 sites. Fetuses with significant congenital structural (including CHD) or chromosomal fetal anomalies are excluded. The primary endpoints are 1) obstetric: delivery <34 completed weeks of gestation (Yes/No); 2) neonatal: a composite of death or two markers of neonatal morbidity – bronchopulmonary dysplasia in children born at <32 weeks of gestation and brain injury on cerebral ultrasound; 3) childhood: The Bayley Scales of Infant and Toddler Development-III Cognitive score at two years of chronological age. The study protocol does not include fetal or post-natal brain MRI, thus limiting the ability to quantify brain maturation or white matter injury.

1.4 Selection of Drugs and Dosages

Progesterone therapy has received approval for prevention of preterm birth. On February 3, 2011, the FDA approved the use of progesterone supplementation (hydroxyprogesterone caproate) during pregnancy to reduce the risk of recurrent preterm birth in women with a history of at least one prior spontaneous preterm delivery.[48] Recently, the Society for Maternal-Fetal Medicine reviewed the existing data and concluded that existing randomized studies indicate that in women with singleton gestations, no prior preterm birth, and short cervical length, vaginal progesterone, either 90-mg gel or 200-mg. suppository, is associated with reduction in preterm birth and perinatal morbidity and mortality, and can be offered in these cases.[49] This was rated as Level I evidence and a level A recommendation. Similarly, the Maternal Fetal Medicine Committee of the Society of Obstetricians and Gynaecologists of Canada reviewed the existing data and concluded that on the basis of the data from the RCTs and a meta-analysis, it is recommended: 1) that in cases where the clinician and the patient have opted for the use of progesterone the following dosages should be used: for prevention of preterm birth in women with history of previous preterm birth:17 alpha-hydroxyprogesterone 250 mg IM weekly (level of evidence I-B) or progesterone 100 mg daily vaginally (level of evidence I-A); and 2) for prevention of preterm birth in women with short cervix, progesterone 200 mg daily vaginally (level of evidence I-A).[50]

The proposed study will utilize a transvaginal Bioadhesive Delivery System consisting of progesterone in a polycarbophil-based gel (Crinone 8%; Serono Laboratories, Norwell, MA) which was designed to provide prolonged and controlled release of progesterone after vaginal application.[2] The labeled indications include secondary amenorrhea, ART in patients who require progesterone supplementation, and ART in patients with partial or complete ovarian failure. An unlabeled use is to reduce the risk of recurrent spontaneous preterm birth in appropriately selected women. The usual dosage is 90 mg (8% gel) once or twice daily. An ongoing multi-institution study to determine if progesterone prophylaxis

during pregnancy has long-term neonatal or infant benefit uses a daily natural vaginal suppository (200 mg).[47] Therapy will be initiated in the early third trimester (24-28 weeks GA). This is a period of rapid fetal brain growth and the period in which brain development in fetuses with CHD lags behind that of fetuses without CHD (see below). Therapy will be continued to 39 weeks GA in order to continue the potential neuroprotective effects, and potentially decrease the prevalence of birth prior to 39 weeks GS. The fetal brain continues to develop throughout gestation and there is evidence (see below) that delivery prior to 39 weeks is associated with increased mortality, morbidity and worse neurodevelopmental outcomes.[51-53]

1.5 Relevant Literature and Data

1.5.1 Congenital Heart Disease and Neurobehavioral Disability

In the United States, approximately 1 in every 100 newborns is diagnosed with CHD. Many of these newborns (25%-35%) will require either corrective or palliative open-heart surgery. As recently as the 1960's, only 20% of newborns with critical CHD survived to adulthood. Today, thanks to better diagnostic technologies and methods (including prenatal diagnosis), advances in surgery, and improved postoperative care, early survival is over 90%. However, with improved early outcomes has come the sobering recognition that there is an ongoing risk of late mortality as well as significant morbidity for these children. Neurodevelopmental disability is now recognized as the most common complication of critical CHD (i.e. those patients requiring cardiac surgery in infancy) and has the most negative impact on quality of life, academic performance, and opportunity for independence as an adult. [54-57]

On average, children with CHD, compared to children in the normal population, have significantly lower scores on IQ and achievement tests, delays in reaching motor milestones, as well as higher frequencies of learning disabilities, use of special services, and speech, language, and behavioral abnormalities.[54, 56] The factors underlying developmental morbidity in children with heart disease are complex and multi-factorial; ranging from genetic conditions and altered blood flow to the brain in utero to postnatal medical conditions that accompany CHD and its medical and surgical therapies. Regardless of their origin, neurodevelopmental problems adversely affect learning and the attainment of academic, social, and vocational skills. Ultimately, they undermine mental health and employment opportunities in adulthood. There is a wealth of disturbing data documenting the impact of neurodevelopmental deficits among children and adults affected by CHD. [57]

The most comprehensive data currently available on the neurodevelopmental sequelae of neonatal cardiac surgery are provided by the Boston Circulatory Arrest Study (BCAS) which examined the incidence of acute neurological injury, as well as later cognitive and behavioral outcomes, following the arterial switch operation (ASO) for transposition of the great arteries (TGA).[58-63] Developmental outcomes were evaluated at 1, 4, and 8 years of age. At the 8-year evaluation, Full-Scale Intelligence Quotient (FSIQ) and Performance IQ (PIQ), memory, visual-spatial skills, and academic achievement for the entire BCAS cohort were lower than population norms. A substantial number of the patients were experiencing academic difficulties and 37% had received special education services. There was a distinctive pattern of neurodevelopmental dysfunction characterized by mild cognitive impairment, impaired executive function, inattention and impulsive behavior, deficits in core communication and pragmatic language skills, as well as impaired social cognition.

Evaluation of the BCAS cohort at 16 years of age has recently been completed.[62, 64] The evaluation focused on neuropsychological domains that previous evaluations suggested

were areas of weakness, including executive functions, memory, attention, and visual-spatial skills. Not only were the mean scores in the cohort significantly lower than those expected in the general population, but the standard deviations were larger, suggesting marked variability among children in their outcomes. The deficits are particularly notable because the adolescents were of normal birth weight (>2.5 kg), did not have genetic syndromes or extracardiac anomalies, and rarely required reoperation or long-term cardiac medications. Evaluation of these adolescents with brain MRI revealed a very high prevalence of white matter injury and altered white matter microstructure.[64]

Most studies of neuroprotection in the CHD population have focused on the intra-operative period and management of CPB. Yet there is no evidence that any of these strategies have resulted in improvements in unadjusted neurodevelopmental outcomes. A recent analysis of 1,770 patients (23 centers/5 countries) born 1996-2009 who underwent cardiac surgery with CPB at age ≤ 9 mo. and were tested with the Bayley Scales of Infant Development-II revealed evidence of only slight improvement over time in Psychomotor Development Index (PDI) and Mental Developmental Index (MDI) scores since 1996, even after adjustment for patient factors. Surgery in more recent years was not associated with better unadjusted PDI or MDI scores.

A recent systematic review evaluated the strength of evidence for current practices for neuromonitoring and neuroprotection during cardiac surgery in infants.[65] The authors reviewed 162 manuscripts and concluded that the level of evidence is insufficient to support the effectiveness of most currently used neuroprotective therapies. These findings suggest that a new approach and new targets for neuroprotection are essential to improve outcomes.

1.5.2 Congenital Heart Disease, Brain Development and White Matter Injury

The utero-placental-fetal circulation is unique in a number of respects that have distinct impacts on fetal cerebral blood flow. In the fetus without CHD, gas exchange occurs in the placenta with oxygenated blood returning through the umbilical vein and ductus venosus (DV) to the portal vein, inferior vena cava (IVC) and right atrium and deoxygenated blood returning to the placenta via the umbilical artery. Prior to birth, blood flow to the lungs is very low due to elevated pulmonary vascular resistance and relatively low lung volumes. Two connections exist between the systemic and pulmonary circulations: 1) the foramen ovale connecting the right and left atria, and 2) the ductus arteriosus between the pulmonary trunk and descending aorta. Umbilical venous blood is preferentially directed through the DV into the left lobe of the liver. As a consequence, the oxygen saturation is higher in the left hepatic veins as they join the IVC resulting in streams of blood with different saturations. The higher saturated stream containing blood from the DV is preferentially directed across the foramen ovale to the left atrium. This blood mixes with the limited amount of pulmonary venous blood returning from the lungs, resulting in a saturation in the fetal left ventricle of approximately 65%, which is then ejected into the ascending aorta and cerebral vasculature. Blood ejected by the right ventricle consists of desaturated venous blood from the superior vena cava (SVC) as well as the relatively desaturated streams from the IVC and coronary sinus. The resulting saturation in the fetal right ventricle is approximately 55%. Blood from the right ventricle is preferentially ejected across the ductus arteriosus returning to the placenta via the umbilical arteries.

In two common forms of congenital heart disease, TGA and hypoplastic left heart syndrome (HLHS), alterations in fetal blood flow may lead to decreased brain oxygen delivery during fetal life. In TGA, the aorta arises from the right ventricle and thus receives

the relatively desaturated blood from the superior vena cava and lower saturation stream of blood in the inferior vena cava. The higher saturated stream from the left hepatic veins is directed normally across the foramen ovale to the morphologic left ventricle. The left ventricle however is connected to the pulmonary trunk and this higher saturated blood is delivered to the lungs and lower body. In HLHS, the fetal circulation is characterized by increased left atrial pressure, resulting in reversal of flow across the foramen ovale. Left ventricular filling is impaired and there is either no ventricular output or stroke volume is diminished with reduced or absent flow into the ascending aorta. All venous return mixes in the right ventricle and is ejected into the pulmonary trunk and ductus arteriosus. Although the resulting saturation is not as low as with TGA, a number of additional factors may restrict flow to the cerebral circulation. Most importantly, in patients with aortic valve atresia, flow to the head and neck vessels only occurs by retrograde flow from the ductus arteriosus via the transverse aorta. Brain growth and maturation are delayed in patients with both TGA and HLHS. Despite the differences in fetal blood flow and blood oxygen saturations between fetuses with TGA and HLHS, post-natal head circumferences and maturation scores are not statistically different. This suggests that oxygen delivery (oxygen content x cerebral blood flow) is the critical value that determines brain growth and maturation.

There is convincing evidence that in utero brain development is abnormal in fetuses with CHD leading to delayed maturation, poor growth, and white matter injury. Altered fetal hemodynamics resulting from the presence of CHD may cause decreased in-utero cerebral blood flow and oxygenation of the fetal brain.[66, 67] In turn, this impairment in blood flow and oxygenation results in impaired brain growth and altered structural and cellular maturation, particularly of the white matter.[68, 69] Fetal imaging studies show that during the third trimester, normally a time of rapid brain growth and development, that the brains of infants with CHD fail to grow at the same rate as brains of fetuses without CHD.[69] During this period of rapid brain growth in the normally developing fetal brain, there is a corresponding increase in the complexity of brain structure, characterized by cortical folding which progresses as a result of white matter volume expansion. During this phase of white matter growth, populations of glial cells called oligodendrocytes (the major constituent of white matter) are undergoing rapid differentiation and maturation. This increase in maturation and organizational complexity is delayed in fetuses with CHD, and is most delayed in those with the most complex defects (e.g. aortic atresia).[66, 68, 69] The figure shows the relationship between the Fetal Total Maturation Score (fTMS) derived from fetal brain MRI for normal fetuses and those with CHD. A lower fTMS implies a less mature brain. There is a delay in brain maturation in the fetuses with CHD in late gestation. Thus, there is convincing evidence for global brain growth impairment in the fetus with CHD resulting in

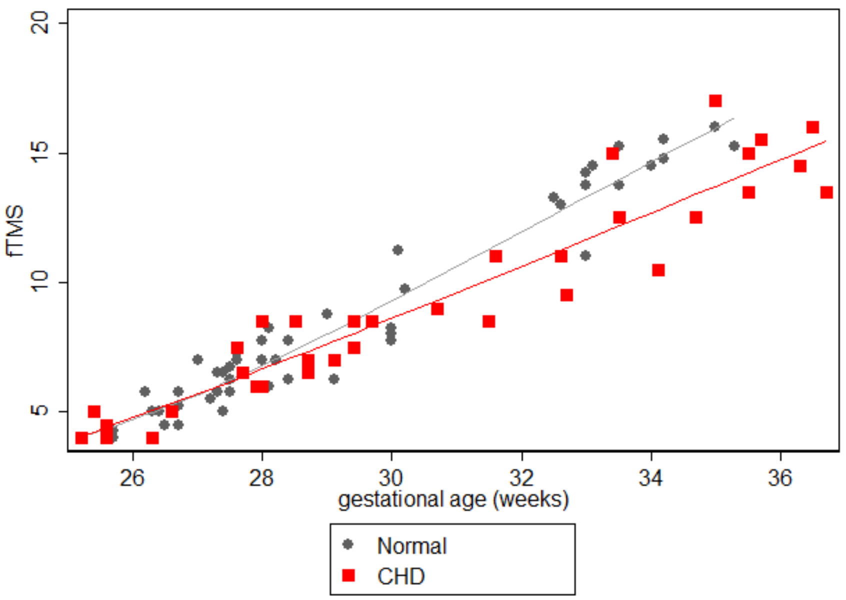

microcephaly, immature cellular elements of the white matter and decreased cortical folding at birth. It has been demonstrated in several prospective observational studies that this abnormal/delayed development is a primary risk factor underlying the hypoxic-ischemic white matter brain injury seen in over 50% of peri-surgical infants with CHD.[27, 28] Pathological examination of this white matter injury demonstrates that this injury (PVL) is identical to that seen in infants born prematurely. Using TMS, it has been demonstrated that risk for pre-operative and postoperative injury seen in infants with is highly correlated with the maturational state of the brain prior to surgery. [67, 68] In addition, brain immaturity at the time of cardiac surgery is a strong predictor of functional outcome at 2 years. [70, 71]

1.5.3 Assessment of Cerebral Blood Flow in the Fetus

Fetal brain growth and development depends upon an adequate supply of oxygen and other substrates. Autoregulatory mechanisms in the fetus alter cerebrovascular resistance to counteract changes in oxygen delivery.[72] Animal models and studies in human fetuses have shown a decrease in cerebrovascular resistance to compensate for fetal hypoxia. This redistribution of fetal circulation has been termed the ‘brain-sparing effect’ and may be an adaptive response to intrauterine hypoxia.

There is increasing evidence that cerebral blood flow (CBF) in the fetus is altered in the presence of CHD. Middle cerebral artery (MCA) blood flow velocities (peak systolic velocity, peak diastolic velocity, and mean velocity) and vascular resistance can be assessed using pulsed-wave Doppler. Pulsatility index (PI) is a measure of vascular resistance in the circulatory bed downstream from the point of Doppler sampling. It is calculated according to the equation, PI = (peak systolic velocity – end-diastolic velocity)/ time-averaged mean velocity.[72] The impact of CHD on fetal cerebrovascular flow patterns is dependent on the specific type of CHD. Studies at CHOP have shown that cerebrovascular resistance is lower than normal in fetuses with HLHS.[72] In fetuses with right sided obstructive lesions (tetralogy of Fallot), cerebrovascular resistance is significantly higher than in fetuses with HLHS and is higher than in normal fetuses, but not to a significant degree. The fetus with HLHS and aortic atresia has absent antegrade blood flow in the ascending aorta, with CBF supplied retrograde via the ductus arteriosus, likely limiting cerebral perfusion. The decreased cerebral vascular resistance seen in the fetus with HLHS may be an attempt to attract a greater amount of CBF to compensate for decreased oxygen delivery.

Fractional moving blood volume (FMBV) is an alternative method to assess the fetal cerebral circulation which uses Power Doppler to estimate quantitatively brain tissue perfusion.[73-76] Using defined regions of interest, FMBV allows assessment of regional differences in brain perfusion.[76] The method has been validated in animal models and is potentially a more sensitive method of brain blood flow redistribution in fetal growth restriction.[76] FMBV analyzes changes in amplitude (power) on the backscattered Doppler ultrasound signals originated from red blood cells. FMBV estimation aims to compensate for the effect that depth, tissue interfaces, and attenuation have on the power Doppler ultrasound signals. FMBV is more sensitive than MCA flow velocity or cerebral placental ratio (CPR) to identify SGA fetuses at risk of abnormal neonatal neurobehavioral performance. SGA fetuses with increased frontal perfusion showed a 30% risk of abnormal neurobehavior expressed in social interactive organization, state organization, and attention capacity.[75] FMBV has not been used to assess the fetal cerebral circulation in fetuses with CHD.

CBF in infants with CHD can also be assessed using a magnetic resonance imaging technique, pulsed arterial spin-label perfusion magnetic resonance imaging. At CHOP, early post-natal (pre-operative) CBF was measured under standard ventilation and repeated after

increased carbon dioxide in 25 terms infants with CHD.[67] Preoperative structural brain abnormalities were seen in more than 50% of the cohort, with the most common being PVL (28%) and microcephaly (24%). Observed CBF values were much lower than what is suggested by limited normal data and critically decreased in some patients. The presence of PVL was strongly associated with low baseline CBF and poor CO_2 reactivity.[67] The poor vascular responsiveness to inspired CO_2 may be due to maximal baseline vasodilation in response to low CBF in these patients. Impaired CO2 reactivity has been associated with poor neurodevelopmental outcome and a higher risk of death.[67]

1.5.4 Serum Progesterone Levels

In humans, progesterone is necessary for fertilization and maintenance of pregnancy (by maintaining myometrial relaxation).[40, 41] Progesterone is initially produced by the corpus luteum and from 7–8 weeks of gestation increasingly by the placenta. At term, in a singleton pregnancy, the placenta produces approximately 250–300 mg progesterone per day.[40] Prior to ovulation, serum levels are less than 1 ng/ml. Serum rise after ovulation for 6-10 days, then fall if fertilization does not occur. If a viable pregnancy occurs, serum levels rise throughout gestation. A characteristic of human pregnancy is that circulating progesterone levels do not fall with the onset of labor.

There is no data concerning fetal serum progesterone levels either during a normal pregnancy, or in response to exogenous progesterone. Although vaginal progesterone is commonly used for in conjunction with assisted reproductive technologies (ART) and to prevent preterm birth, there is very little data concerning placental levels, transplacental transfer or fetal blood levels. A study using ex vivo perfusion of placentas from term pregnancies showed that 17-α-Hydroxyprogesterone caproate is metabolized by placental lobules. Both the parent compound and the metabolites are transferred to the fetal circuit.[6] There is no data on serum progesterone levels during pregnancy in mothers carrying a fetus with CHD. Uterine tissue concentrations of progesterone are significantly higher after vaginal administration than after systemic administration, despite higher blood levels with systemic administration.[3, 4] In a randomized trial, women undergoing hysterectomy received either vaginal or IM progesterone. The ratio of endometrial to serum progesterone concentrations was higher after vaginal administration of progesterone than after IM injections.[3] The lower blood levels after vaginal administration have been associated with a lower prevalence of systemic side effects.[3, 5] Thus maternal serum progesterone levels after vaginal administration will not reflect endometrial or fetal serum concentrations.

1.5.5 Placental Function in Normal and CHD Pregnancies

The placenta is connected to the fetus by the umbilical cord and to the mother via the uterine wall. The umbilical cord contains two arteries and one vein. The placenta is primarily a vascular organ in which the uterine arteries bring maternal blood in contact with the placental villi in the intervillous space, where the trophoblastic epithelium surrounding the villi mediates the transfer of oxygen and other maternally-derived substances to the fetus. The normal placenta is long and thin, and contains numerous placental villi. The combination of a placental villous tree and its decidual artery is called a placentome (also a cotyledon). The diffusion barrier for transfer between mother and fetus is normally very small, made up by the trophoblast basement membrane and the fetal villous capillary endothelium. Fetal capillaries receive fetal deoxygenated blood and fetal waste products from arterioles arising from the umbilical arteries. Oxygenated and nutrient-enriched blood is returned to the fetus via a network of veins that drain into the umbilical vein. The maternal uterine arteries dilate significantly during the course of a normal pregnancy to increase maternal blood flow to the placenta and developing fetus; for example, 50 mL/minute of

maternal blood enters the non-gravid uterus, increasing to approximately 700 mL/minute at near-term. This large increase is made possible by increased maternal blood volume, increased cardiac output, uterine artery dilation, and low uterine and placental vascular resistance. Appropriate placental functioning at the anatomic/histologic level of the placentome requires: 1) maternal uterine and decidual artery integrity, patency, flow, and dilation during pregnancy; 2) increased maternal blood flow and low resistance through the intervillous space; 3) normal exposure of the villous trophoblastic endothelium to maternal blood; 4) low diffusion distance between maternal and fetal blood from the intervillous space across the trophoblast and villous endothelium; 5) integrity and patency of the villous vessels; 6) unobstructed flow in the umbilical cord.

There is increasing awareness of the importance of normal function and regulation of a placental synthetic, metabolic, and inflammatory functions to maintain placental integrity, maternal and fetal well-being, and normal fetal development. A variety of acute, sub-acute, and chronic abnormalities of placental anatomy and vascular function have been linked to maternal abnormalities such as preeclampsia and to fetal events such as fetal demise, IUGR, and perinatal brain injury; the types of injury have included white matter injury, stroke, or neonatal encephalopathy.[77-80] Overall, associations between the development of early and mid-term placental abnormalities and subsequent events such as IUGR, stroke, preeclampsia, and abnormal post-natal brain anatomy and neurodevelopmental outcomes are increasing and becoming more firmly established. Despite this information, examination of the placenta—histopathological or otherwise—is not routine, even in pregnancies complicated by IUGR or early evidence of neonatal brain injury.

There is a significant lack of information about placental anatomy and function in infants born with CHD, despite the increased frequencies of IUGR, pre-term birth, relative brain immaturity, reduced brain size, and brain imaging abnormalities (prior to any cardiac or other interventions), etc. that occur in these infants. A recent study found significantly reduced fetal somatic growth velocity in the later stages of pregnancy as well as reduced birth weight in HLHS infants and suggested suboptimal energy balance due to placental vascular pathology as a potential cause.[51] The increased risk of IUGR in fetuses with CHD was also suggested by a recent retrospective cohort study which found an approximately 2-to-3-fold increased risk of IUGR accompanied the fetal diagnosis of CHD (without chromosomal or extracardiac anomalies.[77] Preliminary data collected recently at CHOP suggests that various placental abnormalities with the potential to alter placental function and fetal homeostasis frequently occur in pregnancies complicated by CHD. Taken together, these data indicate that pregnancies complicated by fetal CHD may be at increased risk for abnormalities of placental anatomy and/or function that can compromise fetal growth and development and lead to increased risk of perinatal brain injury. Thus, a comprehensive and multimodal analysis of placental anatomy, vascularity, and biochemical function is warranted.

1.5.6 CHD and Preterm Birth

There is increasing evidence that even late preterm and early term birth is associated with worse perinatal and long-term neurodevelopmental outcomes in both the normal fetus and the fetus with CHD. In the fetus without CHD, there is significant neonatal morbidity associated with elective delivery at 37–38 weeks compared to deliveries occurring at 39–40 weeks.[81] Neonatal death, respiratory complications, hypoglycemia, sepsis, seizures, necrotizing enterocolitis, hypoxic-ischemic encephalopathy, cardiopulmonary resuscitation or ventilator support within 24 hours after birth, umbilical-cord-blood arterial pH below 7.0, a 5-min Apgar of 3 or less, admission to the neonatal intensive care unit (NICU) and prolonged hospitalization of at least 5 days were significantly less likely to occur as

gestational age increased from 37 (15.3%) to 39 weeks (8%), and plateaued at 40 weeks (7.3%).[81] For the fetus without CHD, postponing elective delivery to 39 weeks may prevent 48% of cases of complications among deliveries at 37 weeks and 27% among 38-week deliveries.

Preterm birth is common for mothers carrying fetuses with CHD. Among 397 babies with CHD delivered in the Special Delivery Unit (SDU) at CHOP in in fiscal years 2011 to 2013, 53% were delivered at < 39 weeks gestational age and 16% at ≤ 37 weeks. Among 971 neonates treated at Boston's Children Hospital from 2002 through 2008, 56% were born at < 39 weeks gestational age and 19% at ≤ 37 weeks.[52] Data from the National Vital Statistics Report (http://www.cdc.gov/nchs/data/nvsr/nvsr62/) show that in 2011, among all births in the United States, 38% were at < 39 weeks gestational age and 12% were at < 37 weeks. These data suggest that the risk of preterm birth is increased for the fetus with CHD.

Late preterm or early term birth also has serious adverse effects for the fetus with CHD. Investigators from Boston Children's Hospital evaluated 971 consecutive neonates with CHD and a known gestational age born from 2002 through 2008.[52] Compared with the referent group of neonates who were delivered at 39 to 40 completed weeks gestation, neonates born at 37 to 38 weeks had increased mortality (6.9% vs. 2.6%; adjusted P=0.049) and morbidity (49.7% vs. 39.7%; adjusted P=0.02) rates and tended to require a longer duration of mechanical ventilation (adjusted P=0.05). They concluded that for neonates with critical CHD, delivery before 39 weeks gestation is associated with greater mortality and morbidity rates and more resource use and that the ideal gestational age for delivery of these patients may be 39 to 40 completed weeks. Evidence from CHOP suggests that younger gestational age is associated with worse neurodevelopmental outcomes in children with CHD.[53] Neurodevelopmental outcomes were evaluated in 351 preschool children with CHD born at 36 weeks or greater gestational age. Performance for cognition, language, executive function, social skills, visual-motor, and fine-motor skills was better for those born at 39 to 40 weeks of GA or more versus those born at less than 39 weeks (all P<.05). These findings are consistent with the hypothesis that delivery before 39 to 40 weeks gestational age is associated with worse outcomes in patients with CHD. Early delivery of a child with CHD is often indicated because of maternal or fetal health issues. In the absence of these concerns, these data suggest that elective (or spontaneous) delivery at 39 to 40 weeks gestational age is associated with better neurodevelopmental outcomes.

The reasons for an increased incidence of preterm birth for fetuses with CHD have not been fully delineated. Early delivery is sometimes required for maternal or fetal health issues. However, there is evidence that placental structure and function are often abnormal in pregnancies where the fetus has CHD, which may contribute to the risk of preterm birth. Preliminary data from CHOP shows that there are multiple abnormalities in the placenta of the fetus diagnosed with CHD, which may impact growth and development, timing of delivery and long-term outcomes such as neurodevelopment and late vascular health. Placentas from 72 pregnancies were examined. Insertion of the umbilical cord into the placenta was eccentric in 61 (84%) and velamentous in 3 (4%). Abnormal maturation of villi was present in 29%; chorioangiosus in 14%. An abnormal fetal/placental weight ratio (as compared to published norms) was seen in 46%. Evidence for inflammation was present overall in 19% and vascular thrombosis in 16%. Infarction was present overall in 14%, but most commonly in HLHS and TGA (25%). There is evidence that the fetus with CHD is at increased risk for intra-uterine growth retardation (IUGR), which may be due in part to uteroplacental insufficiency.[77] In a retrospective cohort study of 67,823 patients, there were 193 cases of fetal CHD (0.3%) and 5,669 cases of IUGR (8.4%). Prenatal diagnosis of CHD

was associated with an increased risk of IUGR (23.8% vs. 8.5%, adjusted odds ratio [aOR] 3.3, 95% confidence interval [CI] 2.4–4.6), and the risk was greatest in fetuses with major CHD (16.5% vs. 8.5%, aOR 2.1, 95% CI 1.3–3.2).[77] Isolated CHD was also associated with an increased risk of IUGR (17.8% vs. 8.5%, aOR 2.2, 95% CI 1.4–3.7). The increased risk persisted after excluding extracardiac and chromosomal anomalies. There was a significantly increased incidence of single umbilical artery in fetuses with CHD, consistent with structural placental abnormalities. There is no data concerning progesterone levels and metabolism in pregnancies with a fetus with CHD. Overall, the existing data suggest that pregnancies with CHD are at increased risk for uteroplacental insufficiency and may be considered stressed or compromised pregnancies.

1.6 COMPLIANCE STATEMENT

This study will be conducted in full accordance all applicable Children's Hospital of Philadelphia Research Policies and Procedures and all applicable Federal and state laws and regulations including 45 CFR 46, 21 CFR Parts 50, 54, 56, 312, 314 and 812 and the Good Clinical Practice: Consolidated Guideline approved by the International Conference on Harmonization (ICH). All episodes of noncompliance will be documented.

The investigators will perform the study in accordance with this protocol, will obtain consent and assent, and will report unanticipated problems involving risks to subjects or others in accordance with The Children's Hospital of Philadelphia IRB Policies and Procedures and all federal requirements. Collection, recording, and reporting of data will be accurate and will ensure the privacy, health, and welfare of research subjects during and after the study.

2 STUDY OBJECTIVES

We propose a Phase II single-center placebo-controlled randomized therapeutic exploratory trial to determine whether there is evidence that progesterone, administered pre-natally, improves neurodevelopmental outcomes, improves brain development, and reduces perioperative white matter injury in neonates with CHD undergoing cardiac surgery. The data provided by this therapeutic exploratory study will aid in selection of appropriate outcomes and inform sample size calculations for a future multi-institutional study.

2.1 Primary Objective (or Aim)

Primary Aim: Develop preliminary evidence to support a multi-institutional study to determine whether, in women carrying fetuses with CHD (maternal-fetal dyad), prophylactic vaginal natural progesterone therapy is neuroprotective, and compared to placebo improves neurodevelopmental outcomes at 18 months of age.

Hypothesis:

Compared to placebo, progesterone administration results in a higher score on the Motor Scale of the Bayley Scales of Infant and Toddler Development-III (Bayley-III) at 18 months of age.

2.2 Secondary Objectives (or Aims)

Specific Aims: Develop preliminary evidence to support a multi-institutional study to determine whether, in women carrying fetuses with CHD (maternal-fetal dyad), prophylactic vaginal natural progesterone therapy is neuroprotective, and compared to placebo:

1. improves fetal brain growth and maturation,
2. increases myelination during fetal brain development,
3. reduces pre-operative brain white matter injury, and
4. reduces post-operative white matter injury.

Hypotheses:

Compared to placebo, progesterone administration results in

1. higher scores on the Cognitive and Language Scales of the Bayley Scales of Infant and Toddler Development-III (Bayley-III) at 18 months of age,
2. a higher total maturation score (TMS) on early post-natal MRI,
3. Increased global and regional brain volumes
4. a greater volume of myelinated white matter (VMWM) in the posterior limb of the internal capsule on early post-natal, pre-operative MRI,
5. lower periventricular leucomalacia (PVL) volume on early post-natal, pre-operative MRI,
6. lower PVL volume on post-operative MRI.

3 INVESTIGATIONAL PLAN

3.1 General Schema of Study Design

Phase II single-center placebo-controlled randomized therapeutic exploratory trial

3.1.1 Screening Phase

Potential subjects will be identified by clinically indicated fetal echocardiography in the Fetal Heart Program Clinic by one of the investigators (J.R. or A.S.). Potential subjects will be screened for eligibility at 24-28 weeks GA by a study nurse using the protocol inclusion and exclusion criteria. Subject permission (informed consent) will be obtained prior to any study related procedures being performed.

3.1.2 In Utero Treatment Phase

After informed consent is obtained, the subject will undergo baseline evaluation including medical history review, physical examination, obstetric ultrasound, fetal echocardiography, and fetal brain MRI. Maternal and paternal blood samples will be obtained. Progesterone levels will be assessed in the mother. Serum samples will be banked for later assessment of progesterone metabolites and biomarkers. The maternal and paternal samples will be used for genetic testing. Subjects will be randomized to study drug or placebo. For scheduling reasons, off site SD initiation may occur. After randomization, the subjects will return for routine prenatal visits every 2 weeks until 36 weeks GA and weekly thereafter. Obstetric ultrasound and fetal echocardiography will be performed every 4 weeks and as clinically indicated. Maternal blood tests will be repeated every 4 weeks. Fetal brain MRI will be obtained at 34-36 weeks GA. The study drug will be continued until 39 weeks GA, the onset of spontaneous labor if prior to 39 weeks GA, or the time of induction of labor or cesarean section for maternal or fetal health concerns. At the time of delivery, maternal and cord blood samples will be obtained. A portion of the placenta will be obtained.

3.1.3 Post-Natal Phase

After birth, the child will undergo clinically indicated evaluation and treatment. Data will be collected from the medical record concerning diagnosis, associated anomalies, medical and surgical management, and the hospital course. A blood (either from the cord or from an

existing blood drawing line during a hospitalization, routine clinical labwork or at the time of a research draw for a separate study) or saliva sample will be obtained for genetic testing. An early post-natal brain MRI will be obtained on the day of cardiac surgery, during the same anesthetic but prior to surgery. Post-operative brain MRI will be obtained within 10 days of surgery.

3.1.4 Neurodevelopmental Evaluation

All surviving infants will undergo neurodevelopmental evaluation at 18 months of age (corrected for prematurity). An interim medical history will be obtained, including details of any hospitalizations and surgical procedures. A neurological examination will be performed. Growth parameters will be assessed. The neurodevelopmental evaluation will include the Bayley Scales of Infant and Toddler Development-III, the Modified Checklist for Autism in Toddlers (M-CHAT), and the Infant-Toddler Social and Emotional Assessment (ITSEA).

3.2 Allocation to Treatment Groups and Blinding

3.2.1 Randomization

The study biostatistician, Dr. Putt, will generate a block randomization schedule based on stratification by fetal CHD diagnosis: TGA, HLHS, and other CHD. The randomization schedule will be based on equal allocation of subjects to the progesterone and placebo arms within each block. To ensure blinding, the randomization schedule will be generated using variable block sizes. Upon confirmation of eligibility in the DMS, the randomization module will be programmed to generate a randomization number that corresponds to a unique kit number of progesterone or placebo treatment. This kit number will be used to distribute treatment.

3.2.2 Blinding

All primary study team members will be blinded to treatment arm. The database administrator will have access to the information that links the kit number to the treatment arm. He/she will provide this information to the Investigational Pharmacy so that kits are prepared according to this scheme. Progesterone and placebo treatment kits will appear identical. They will be labeled with the following information:

1. Kit number
2. Study name and number
3. Subject ID # [blank line to record this number]

3.2.3 Unblinding

Unblinding can occur if there are concerns over maternal or fetal health, or if requested by the DSMB. A direct request from the PI to the Investigational Pharmacy will be used to initiate unblinding. All requests for unblinding will be documented and reported to the DSMB.

3.3 Study Duration, Enrollment and Number of Sites

3.3.1 Duration of Study Participation

The study duration for each mother will consist of the in-utero treatment phase beginning at 24-28 weeks GA and continuing until 30 days after stopping study drug or after delivery. The study duration for each fetus/child will include the in-utero treatment phase, the post-natal phase, and the neurodevelopmental evaluation. The study will begin at 24-28 weeks GA and continue to 18 months of age (corrected for prematurity).

3.3.2 Total Number of Study Sites/Total Number of Subjects Projected

The study will be conducted at a single site, the Children's Hospital of Philadelphia. We plan to enroll sufficient patients to have 80 evaluable patients (40 per treatment group) at the 18-month neurodevelopmental testing. Based on known clinical outcomes for this patient population and experience with other studies with similar follow-up plans, we estimate a 5% early mortality and a further 20% attrition from all causes between hospital discharge and the 18-month evaluation. There may also be dropout prior to birth for a variety of reasons: fetal death, intolerance of therapy, withdrawal from study. We do not have accurate data to estimate this loss. We anticipate needing to enroll 110-130 patients to have a sufficient cohort at 18 months (40 per treatment group), with 60 and 52-56 patients, respectively, per treatment group available for the post-natal and post-operative brain MRIs. Based on current patient volume in the Fetal Heart Program Clinic, we anticipate there will be 70-75 eligible patients each year. If the consent rate is 65%, we will be able to complete enrollment within 3 years.

3.4 Study Population

3.4.1 Inclusion Criteria

1) Mothers carrying a fetus with CHD (maternal-fetal dyad) requiring surgery with CPB prior to 44 weeks corrected GA identified prior to 28 weeks GA will be eligible.
2) Informed consent

3.4.2 Exclusion Criteria

1) Major genetic or extra-cardiac anomaly other than 22q11 deletion
2) Language other than English spoken in the home
3) Known sensitivity or listed contraindication to progesterone (known allergy or hypersensitivity to progesterone, severe hepatic dysfunction, undiagnosed vaginal bleeding, mammary or genital tract carcinoma, thrombophlebitis, thromboembolic disorders, cerebral hemorrhage, porphyria)
4) Prescription or ingestion of medications known to interact with progesterone (e.g. Bromocriptine, Rifamycin, Ketoconazole or Cyclosporin)
5) Maternal use of progesterone within 30 days of enrollment
6) History of preterm birth or short cervix (defined as cervical length ≤ 25 mm at 18-24 weeks GA necessitating progesterone therapy
7) Multiple gestation
8) Maternal contraindication for MRI
   Subjects with a known history of non-compliance with medical therapy.

Subjects that do not meet all of the enrollment criteria may not be enrolled. Any violations of these criteria must be reported in accordance with IRB Policies and Procedures.

4 STUDY PROCEDURES

4.1.1 Screening Visit

- Confirmation of Eligibility
- Informed Consent
- Medical Record Review
- Fetal Echocardiography (including assessment of CBF)

4.1.2 Randomization Visit (for scheduling reasons subjects may be offered off site study drug initiation)

- If not previously obtained at the screening visit, Informed Consent will be obtained prior to study procedures being performed
- Maternal Blood Tests
- Maternal and Paternal Blood for Genetic Testing
- Fetal Brain MRI

4.2 In Utero Treatment Phase

Subjects will be randomized to study drug or placebo. After randomization, the subjects will return for routine prenatal visits every 2 weeks until 36 weeks GA and weekly thereafter. Obstetric ultrasound and fetal echocardiography will be performed every 4 weeks and as clinically indicated. Maternal blood tests will be repeated every 4 weeks.

4.2.1 Prenatal Visit 1

- Medical Record Review
- Fetal Echocardiography (including assessment of CBF)
- Dispense Study Drug

4.2.2 Subsequent Prenatal Visits (every 2 weeks until 36 weeks GA, then weekly)

- Medical Record Review
- Fetal Echocardiography (including assessment of CBF) (every 4 weeks or as clinically indicated)
- Maternal Blood Tests (every 4 weeks)
- Collect unused study drug
- Dispense study drug
- Assess possible adverse events

4.2.3 Follow-up Fetal Brain MRI at 34-36 Weeks GA

4.2.4 Delivery

- Medical Record Review
- Maternal and Cord Blood Tests
- Collect unused study drug
- Assess possible adverse events

4.2.5 Post-Natal Phase

After birth, the child will undergo clinically indicated evaluation and treatment. Data will be collected from the medical record concerning diagnosis, associated anomalies, medical and surgical management, and the hospital course. An early post-natal brain MRI will be obtained on the day of cardiac surgery, during the same anesthetic but prior to surgery. Post-operative brain MRI will be obtained within 10 days of surgery.

If blood sample for genetic testing was not previously obtained during the post-natal hospitalization as a result of the discontinuation of all nonessential on-campus research due to COVID-19 or it was determined there was insufficient DNA for analysis, consent will be obtained using the consent addendum prior to obtaining additional samples.

4.2.6 Post-Natal Hospitalization

- Evaluation by dysmorphologist
- Infant Blood for Genetic Testing
- Medical Record Review
- Assess possible adverse events
- Early post-natal brain MRI (day of surgery)
- Post-operative brain MRI (within 10 days of surgery)

4.2.7 Subsequent Hospitalization/Outpatient visit

- Infant Blood for Genetic Testing from an existing blood drawing line or drawn at the time of routine clinical labwork (if cord or infant blood not previously obtained during the post-natal hospitalization as a result of the discontinuation of all nonessential on-campus research due to COVID-19 or if it was determined there was insufficient DNA for analysis)
- Maternal and Paternal Blood or Saliva for Genetic Testing (if blood not previously obtained as a result of the discontinuation of all nonessential on-campus research due to COVID-19 or if it was determined there was insufficient DNA for analysis)

4.2.8 Neurodevelopmental Evaluation

All surviving infants will undergo neurodevelopmental evaluation at 18 months of age (corrected for prematurity). An interim medical history will be obtained, including details of any hospitalizations and surgical procedures. Growth parameters will be assessed. A neurological examination will be performed. The neurodevelopmental evaluation will include the Bayley Scales of Infant and Toddler Development-III, the Modified Checklist for Autism in Toddlers (M-CHAT), and the Infant-Toddler Social and Emotional Assessment (ITSEA).

4.2.9 Neurodevelopmental Evaluation and End-of-Study

- Physical and neurological examination
- Evaluation by dysmorphologist (may be performed via telephone or video conference due to the potential risks of COVID-19)
- Vital signs and growth parameters
- Medical record review
- Assess possible adverse events
- Bayley Scales of Infant and Toddler Development-III
- Modified Checklist for Autism in Toddlers (M-CHAT)
- Infant-Toddler Social and Emotional Assessment (ITSEA).
- Child saliva sample for genetic testing (if cord or infant blood not previously obtained during the post-natal hospitalization as a result of the discontinuation of all nonessential on-campus research due to COVID-19 or if DNA quantity was determined to be insufficient)
- Maternal and paternal blood or saliva for genetic testing (if blood not previously obtained as a result of the discontinuation of all nonessential on-campus research due to COVID-19 or insufficient DNA for analysis)

4.2.10 Subject's Home

- Maternal, paternal or child saliva or buccal epithelial cell sample for genetic testing (if cord or infant blood not previously obtained during the post-natal hospitalization as a result of the discontinuation of all nonessential on-campus research due to COVID-19 or if DNA quantity was determined to be insufficient)

4.3 Concomitant Medication

All prior and concomitant medications used by the mother within 30 days prior to the screening visit and through the end of the study will be recorded. The dates of administration, dosage, and reason for use will be included.

4.4 Rescue Medication Administration

In previous studies, the vaginal progesterone gel has been well tolerated with few adverse effects. The most common effect is vaginal irritation which will be treated with sitz baths; and for severe cases a low potency topical steroid cream (hydrocortisone 1 % cream). If a woman goes into preterm labor, this will be treated according to standard clinical protocols.

4.5 Subject Completion/Withdrawal

Subjects may withdraw from the study at any time without prejudice to their care. They may also be discontinued from the study at the discretion of the Investigator for lack of adherence to study treatment or visit schedules and adverse events (AE). The Investigator may also withdraw subjects who violate the study plan, or to protect the subject for reasons of safety or for administrative reasons. It will be documented whether or not each subject completes the clinical study. If the Investigator becomes aware of any serious, related adverse events after the subject completes or withdraws from the study, they will be recorded in the source documents and on the CRF. For women without a history of preterm birth, there are no known risks to progesterone withdrawal.

4.5.1 Early Termination Study Visit

No procedures are required to be completed prior to withdrawal

5 STUDY EVALUATIONS AND MEASUREMENTS

5.1 Screening and Monitoring Evaluations and Measurements

5.1.1 Pre-Natal Medical Record Review

Please see Appendix

5.1.2 Post-Natal Medical Record Review

Medical Chart Review

Please see Appendix.

5.1.3 Physical Examination

Maternal Examination:

- General physical examination (at initial visit).
- Blood pressure/ pulse (at each visit).
- Height/Weight/BMI /Fundal height (at each visit).

- Pelvic examination /Clinical pelvimetry (third trimester).

Infant Evaluation:

CICU Admission: Each child will undergo clinically indicated physical examination, laboratory evaluation and imaging studies.

Genetic Screening by a Dysmorphologist: All children entered into the study will be evaluated for other syndromic diagnosis by Dr. Elaine Zackai, a dysmorphologist. In any study of the neurologic outcome of patients with CHD, it is important to determine whether there is an underlying syndromic diagnosis that in and of itself would impact on outcome.

5.1.4 Vital Signs

Vital signs will be monitored and recorded as clinically indicated.

5.1.5 Laboratory Evaluations

Maternal Evaluation: As part of routine prenatal care, each mother will undergo clinically indicated testing. The laboratory tests listed below should have been obtained by the referring physician at the initial prenatal care visit. If not previously collected, clinically indicated testing will be completed as standard of care with the timing determined by the clinicians caring for the mother.

Clinical Laboratory Tests:

- Hemoglobin/hematocrit /platelet count (to be repeated in the third trimester-36 weeks).
- Blood type and antibody screen (to be repeated at 24-28 weeks).
- Rubella titer.
- Urine (protein, sugar, blood, leukocyte esterase at each visit).
- Diabetes screen (at 24-28 weeks).
- Hepatitis B surface antigen.
- Hepatitis C antibodies (optional).
- HIV (to be repeated in the third trimester-36 weeks).
- Syphilis test (to be repeated in the third trimester-36 weeks).
- Pap smear.
- Urinary drug screen (as clinically indicated).
- Urine culture.
- Gonorrhea and Chlamydia cultures (to be repeated in the third trimester-36 weeks).
- Group B streptococcus culture (to be obtained at 36 weeks).

Other Evaluation (only if clinically indicated):

- Cytomegalovirus, herpes simplex, toxoplasmosis, and varicella maternal titers, hemoglobin electrophoresis, Tay-Sacks, fragile X, cystic fibrosis and spinomuscular carrier screen, and parental karyotype will be offered to some.
- Screening for chromosomal abnormalities: first trimester screen, Quad screen, non-invasive prenatal testing (NIPT).
- Fetal karyotype (by chorionic villous sampling or amniocentesis).

- Chromosomal or genetic evaluation through maternal serum assessment for free fetal DNA.

Infant Evaluation: As part of routine care, each infant will undergo clinically indicated testing.

5.1.5.1 Pregnancy Testing

Not applicable

5.1.6 Other Evaluations, Measures

Neonatal Admission Questionnaires

Demographic, Ethnicity and Socioeconomic Status (SES) Forms: demographic information will be collected in order for the study team to maintain contact for 18-month follow-up. In addition, self-report ethnicity, level of education, and type of employment will be obtained on both mother and father. This information will serve as data to calculate the Hollingshead score, a measure of SES.

Maternal and Cord Blood Tests

Maternal blood will be obtained at the first visit (unless off site study drug initiation has occurred), each subsequent prenatal visit and at the time of delivery. Maternal blood for genetic testing (if blood not previously obtained as a result of the discontinuation of all nonessential on-campus research due to COVID-19 or insufficient DNA for analysis) will be obtained during a child's subsequent hospitalization or outpatient visit to CHOP. Cord blood will be obtained at the time of delivery. Infant blood will be obtained during a hospitalization through the infant's existing blood drawing line or during routine clinical bloodwork if cord blood was not obtained at the time of delivery or DNA quantity was determined to be insufficient for analysis. Maternal blood will be collected into four plasma preparation tubes (K-EDTA, 4 mL whole blood each) on ice, centrifuged at 600 X g for 5 min at 4 ^{o}C, and the plasma supernatant aliquoted into separate 2 mL cryovials (to avoid repeated freeze-thaw events) and the red blood cells returned to each of the four preparation tubes (to avoid repeated freeze-thaw events). The cryovials containing separates plasma samples are rapidly frozen in liquid nitrogen and then stored at –80 ^{o}C (Clinical and Translational Research Center and CHOP Clinical Laboratory) for subsequent assay (ELISA or qRT-PCR). The same procedures will be used to collect and store umbilical venous blood at the time of delivery. One aliquot will be sent for assessment of progesterone levels. Additional samples will be stored at -80 C for later assessment of progesterone metabolites and biomarkers of placental function. The serum progesterone levels will be performed by the CHOP Clinical Laboratory. The total blood volumes over the course of the study will be: Maternal: 90 ml; Paternal: 5 ml; Cord Blood: 22 ml; Infant 5 ml (only if cord blood not obtained). If DNA quantity was determined to be insufficient an additional 5 mL maternal, paternal, and infant sample may be obtained. If blood will be obtained at the time of a research draw for a separate study the amount drawn will not exceed the lesser of 50 mL or 3 mL/kg, even when a participant is providing samples for multiple studies.

Saliva Samples

Blood is the preferred specimen for DNA collection. However, at times a blood draw will not be possible to obtain. In those instances, we will obtain a saliva sample or a sample from a swab of the inner aspect of the cheek. The saliva may be obtained from enrolled subjects by the study team either in person or by mail.

Placenta

Gross Examination:

The placenta will be weighed and examined visually to determine the location of umbilical cord insertion, the types and number of umbilical vessels, and to identify and quantify macroscopic areas, size, and location of abnormalities such as calcification, infarction, hemorrhage, etc.

Tissue Histology and Biochemistry:

The placenta is a heterogeneous organ with regard to normal functions and pathophysiologic injury. Specific tissue sampling strategies to limit bias and maximize analytical accuracy and precision have been described.[82-84] In brief, it is necessary to analyze multiple samples to ensure that the result is an adequate representation of the entire organ. To this end, a systematic uniform random stereologic sampling design will be employed to generate full-thickness samples (approximately 8-12 per placenta, depending upon the size of the organ the random orientation of the sampling grid).[82, 83] Each specimen will be divided in equal portions for 1) fixation and histochemical analyses and 2) rapid freezing and subsequent molecular and biochemical determinations.

Genetic Testing

Sample Procurement and Preparation: Whole blood will be obtained from the biological parents and from the infant through an existing blood drawing line during a hospitalization or during routine clinical bloodwork. The blood samples will be sent directly to Center for Applied Genomics (CAG) for DNA extraction and Cell Line preparation. The total blood volumes over the course of the study will be: Maternal: 86 ml; Paternal: 5 ml; Cord Blood: 22 ml; Infant 5 ml (only if cord blood not obtained). If DNA quantity was determined to be insufficient an additional 5 mL maternal, paternal, and infant sample may be obtained.

APOE Genotyping: Genomic DNA for analysis will be obtained from each peripheral blood sample or saliva collection kit. Crude leukocyte nuclei are prepared from peripheral blood by standard techniques. High molecular weight DNA will be extracted via the Genepure automated nucleic acid extractor (Applied Biosystem Inc.) utilizing the supplied reagents and protocol. A sample of genomic DNA will be used to determine APOE genotypes using a polymerase chain reaction (PCR) based restriction enzyme isoform genotyping protocol. The remaining DNA will be banked at 20 °C for future analyses. The *APOE* genotype will be used as a covariate in the analysis of the neurodevelopmental testing results.

Genome Wide Association Testing: Genome Wide Association Testing will be performed by CAG, at The Children's Hospital of Philadelphia. CAG is experienced in SNP genotyping at an industrial scale. The Illumina technology uses single tube whole genome amplification followed by primer-extension without PCR or ligation. Infant and biological parent blood samples will be processed by CAG. The GWAS will be used to evaluate the parent-child trios for possible copy number variations (CNV). The CNV data will be used as a covariate in the analysis of the neurodevelopmental testing results. We will ask the parents for permission to bank the DNA for future studies and to share the results with other investigators.

5.2 Efficacy Evaluations

5.2.1 Obstetric Ultrasound

The ultrasound examination will be conducted with real-time scanners, using a transabdominal or a transvaginal approach or both. All examinations will be performed by one of three experienced perinatal sonographers using the same ultrasound equipment, who will be blinded to the patient group assignment. The abdominal and transvaginal transducers to be used will include frequencies ranging from 3 MHz to 10 MHz or higher to provide superior resolution while allowing adequate penetration. The detailed obstetric ultrasound examination to be performed at the initial evaluation will include cardiac activity, fetal presentation, amniotic fluid index (AFI), placental position, fetal biometry, an anatomic survey. The maternal cervix (normal, funneled, short or long (in centimeters) and adnexa will also be examined (presence, location, and size of uterine or adnexal masses).

Cardiac activity: fetal heart rate and rhythm.

Fetal presentation: cephalic, breech, transverse, or variable.

Amniotic fluid index: less than 5 cm, oligohydramnios and more than 24 cm, polyhydramnios, or deepest amniotic fluid vertical pocket (less than 2 cm, oligohydramnios and more than 8 cm, polyhydramnios).

Placental position: anterior, posterior, fundal, lateral, low-lying or previa.

Placental cord insertion: central, marginal, eccentric, velamentous.

Fetal Biometry at initial Examination:

- Biparietal diameter (BPD) will be obtained at the level of the thalami and cavum septi pellucid, and the measurement will be taken from the outer edge of the proximal skull to the inner edge of the distal skull.
- Head circumference (HC) will be measured at the same level as the BPD, around the outer perimeter of the calvaria.
- Femoral length (FL) and humeral length (HL) will be measured with the beam of insonation perpendicular to the shaft, excluding the distal femoral or humeral epiphysis.
- Abdominal circumference (AC) will be determined at the skin line on a true transverse view at the level of the junction of the umbilical vein, portal sinus, and fetal stomach when visible.
- Estimated fetal weight (EFW) will be obtained using a combination of measurements such as BPD, HC, AC and FL from previously described formulas, expressed in grams, and compared with weight percentiles from published nomograms.

Elements of the Fetal Anatomic Survey:

- Head, Face, and Neck: Cerebellum, choroid plexus, cisterna magna, lateral ventricles, midline falx, cavum septi pellucidi, third ventricle, corpus callosum, posterior fossa, cerebellum, transcerebellar diameter (in centimeters), profile, nose, nasal bone, orbits, lips and palate.
- Chest-Heart: Four-chamber view, outflow tracts, situs, fetal heart rate and rhythm.
- Abdomen: Stomach (presence, size, and situs), kidneys, bladder, umbilical cord insertion into the fetal abdomen, umbilical cord vessel number.

- Spine: Cervical, thoracic, lumbar, and sacral spine.
- Extremities: Legs and arms (presence or absence). Femoral and humeral length in centimeters. Other long bone lengths will be obtained as clinically indicated.
- Genitalia (male, female, ambiguous).

Fetal Biometry at Repeat Examination: (ultrasound for growth to be performed at 4-week intervals)

- BPD, HC, FL, HL, AC, EFW.
- Fetal presentation.
- Amniotic fluid index and/or amniotic fluid deepest vertical pocket.
- Placental position (placental position at the initial evaluation may not correlate with its location in the third trimester).
- Maternal cervix (cervical length will be measured transabdominally or transvaginally at each sonographic evaluation until 32 weeks gestation) and presence, location, and size of uterine and adnexal masses.

Doppler Ultrasonography:

Doppler color flow mapping with reduced color scale will be used to identify the ductus venosus (DV) and the middle cerebral artery (MCA) and average color scale to locate the umbilical artery (UA) and maternal uterine arteries (UtA). Pulsed- wave Doppler will be used to determine blood flow velocities of the UA, UtA, DV and MCA. The peak systolic velocity, peak diastolic velocity, mean velocity, systolic to diastolic ratio (SD), pulsatility index (PI) resistive index (RI) and UA PI/MCA PI with a mixture from stable signals during fetal apnea will be obtained for each of the aforementioned vessels at the initial evaluation at 24-28 weeks and then every 4 weeks.

5.2.2 Fetal Echocardiography

All patients will have standard fetal echocardiographic examinations and Doppler flow studies at the time of enrollment and then every 4 weeks (or as clinically indicated) until 37-39 weeks of gestation. The studies will be performed by two to three dedicated cardiac sonographers under direct supervision of a pediatric cardiologist. The examinations will be performed using a Siemens ACUSON Sequoia 512 ultrasound system or equivalent coupled with either a 6 C2 or an 8V3 MHz transducer (Siemens, Erlangen, Germany). Multiple two-dimensional views will be obtained to evaluate fetal heart anatomy. Doppler interrogation will be performed to evaluate valve competence, stenosis, and shunting. M-mode will be used to assess cardiac rhythm. Color-flow mapping will be used to verify the anatomy, as well as intracardiac flow. The two-dimensional images will be recorded on standard video clips for subsequent analysis. Doppler samples will be obtained at the following sites: aortic valve, pulmonic valve, atrioventricular valve inflows, transverse aortic arch, ductus arteriosus, umbilical vein, DV, MCA, UA, and UtA.

For the aortic outflow, the sample volume will be placed at the level of the aortic valve on either a 5-chamber view of the fetal heart or on a long axis view of the fetal heart. For the pulmonic outflow, the sample volume will be placed at the level of the pulmonic valve on either a right ventricular outflow tract view or on a ductal view. The transverse aortic arch will be sampled on the aortic arch view while the ductus arteriosus will be sampled on the ductal view. The MCA will be sampled in the mid-portion of the vessel while the umbilical artery will be sampled in a free-floating loop. The UtA will be sampled below the crossing

point of the internal iliac artery. The velocity waveforms will be obtained using an angle of insonation lower than 20°. The length of the sample volume will range between 1 and 4 mm. All recordings will be performed during periods of both fetal rest and apnea. Aortic valve diameters will be assessed from the two-dimensional images of apical five-chamber views or long axis views of the fetal heart. Pulmonic valve diameters will be assessed from the two-dimensional images of the right ventricular outflow tract view or the ductal view of the fetal heart. The measurements will be performed from video clips during systole and only the maximum diameters will be selected. Measurements will be repeated on a minimum of 3 different cardiac cycles for each valve and the values obtained will be averaged. Valvular area of the aortic and pulmonic valves will be calculated assuming a circular cross-section, according to the formula π X (valve diameter/2)$^{2.}$ The velocity time integral across both the aortic and pulmonic valve will be calculated at the area under the curve of the aortic and pulmonic outflow Doppler samples, as described above. Absolute right (RVO) and left (LVO) ventricular output (mL/min) will be derived by multiplying aortic and pulmonic valvular area, fetal heart rate and time velocity integral across the respective semilunar valve. The combined ventricular output (CVO= RVO + LVO) and the ratio between RVO and LVO (RVO/LVO) will then be calculated.

The pulsatility index (PI) for the MCA, UA, UtA, and ductus arteriosus will be calculated according to the formula: (peak systolic velocity – end-diastolic velocity)/time-averaged mean velocity. The direction of flow in the transverse aortic arch will be recorded as all antegrade, all retrograde, or bidirectional. Any ductal constriction will be noted. A qualitative assessment of left and right ventricular cardiac function will be made. Finally, the presence or absence of hydrops fetalis will be noted.

5.2.3 Fetal Cerebral Blood Flow

MCA Flow Velocities and Pulsatility Index

Doppler color flow mapping with reduced color scale will be used to identify the circle of Willis and the MCA. Pulsed-wave Doppler will be used to determine blood flow velocities in the UA, ductus venosus (DV) and MCA. The peak systolic velocity, peak end- diastolic velocity, and time averaged mean velocity are calculated on three consecutive samples during fetal apnea, and the results averaged. The pulsatility index (PI), a measure of vascular resistance in the circulatory bed downstream from the point of Doppler sampling will be calculated according to the equation, PI = (peak systolic velocity – end diastolic velocity)/time averaged mean velocity. The ratio of UA-PI to MCA-PI will be designated the U/C-PI ratio. PI measurements of the aforementioned vessels will be converted to gestational age-based Z-scores to allow comparisons between the treatment and control group with previously published normal values.

Fractional Moving Blood Volume

Ultrasound and power Doppler studies will be performed transabdominally, using a Phillips iU 22 (Koninklijke Phillips N.V Eindhoven, The Netherlands) ultrasound system with a C-9 MHz linear curved array transducer in the absence of fetal respiratory and body movements, and with mother and fetus in suspended respiration. The following anatomical planes of the fetal brain will be included in the pulse Doppler ultrasound color box: anterior and complete mid-sagittal, and oblique at the level of the posterior fossa. During the mid-sagittal views, special care will be taken to insonate through the sagittal suture in order to avoid the bone barrier and have an uncluttered power Doppler ultrasound recording. The size of box will be maintained as small as possible, in order to cover the different regions under investigation and to improve the frame rate. The ultrasound and power Doppler ultrasound settings to be used in all examinations will include: standard gray-scale images

for obstetrics, medium persistence, high sensitivity, normal image display, pulse repetition frequency 610 Hz, medium wall filter, and gain level just below the presence of noise. The mechanical and thermal indices will always be maintained below 1. A minimum sequence of 10 consecutive good quality images without flash artifacts would be stored digitally and transferred to a personal computer for FMBV analysis in software designed specifically for the purpose. (MatLab, MathWorks, Natick, MA, USA). The regions of interest (ROI) will be defined and delimited as follows: 1) anterior cerebral, in an anterior mid sagittal view of the fetal head, delimited anteriorly by the internal wall of the skull, posteriorly by an imaginary line drawn at 90° at the level of the origin of the anterior cerebral artery and parallel to an imaginary line in the front of the face, and inferiorly by the base of the skull, 2) complete mid-cerebral, in a mid- sagittal plane delimited by the complete internal wall of the fetal skull and 3) posterior fossa, delimited anteriorly and by the base of the cerebellar hemispheres and posteriorly by the fetal skull. Each image will be rendered and the pixels containing color information within the ROI will be selected for analysis. FMBV will be calculated according to the methodology described elsewhere.[85, 86]

5.2.4 Fetal Brain MRI

Prenatal MRI examinations will be performed once at 25-28 weeks GA and once at 34-36 weeks GA.

The prenatal MRI sequences will include:

1. T2 weighted images including half-Fourier acquisition single shot turbo spin echo T2 images in axial, sagittal, and coronal planes.
2. Echoplanar images at short and long TE to assess for hemorrhage.
3. T1 fast low angle shot images (if bleeding or clotting is suspected on other images)
4. Magnetic resonance spectroscopy.
5. Diffusion imaging.

Analysis of the fetal Brain MRI examinations will include:

1. Evaluation for anatomic abnormalities (malformations) and in-utero gross insults.
2. Size of ventricles.
3. Assessment of hemorrhage.
4. Fetal total maturation score (fTMS) evaluation which assesses myelination, cortical folding, and the germinal matrix evolution.
5. Assessment of brain volumetrics.
6. MR spectroscopic assessment of choline, N-acetylaspartate, lactate, and creatine metabolites, including corresponding ratios.

5.2.5 Post-Natal Brain MRI

Postnatal MRI examinations will include an MRI on the day of surgery and one within 10 days after surgery.

The postnatal MRI sequences will include the following:

1. T1-weighted volumetric imaging.
2. T2-weighted imaging.
3. Susceptibility weighted imaging.
4. Diffusion imaging including diffusion tensor imaging.
5. Magnetic resonance spectroscopy

6. Phase contrast flow assessment in the great vessels of the neck and superior sagittal sinus.

Analysis of the postnatal MRI will include:

1. Evaluation for anatomic abnormalities (malformations) and gross insults.
2. Qualitative and quantitative assessment of overt white matter injury (PVL).
3. Assessment of hemorrhage.
4. Postnatal total maturation score (TMS) evaluation which included assessment of cortical folding, myelination, germinal matrix evolution, and migrating bands in the frontal lobes.
5. Assessment of whole brain and segmented brain substructure volumetrics including volume of myelinated white matter (VMWM).
6. Assessment of diffusion parameters.
7. Assessment of flow in the great vessels to and from the brain by phase contrast.
8. Assessment of choline, N-acetylaspartate, lactate, and creatine metabolites, including corresponding ratios in the white matter.

5.2.6 Neurodevelopmental Evaluation

Contact Information Verification Follow-Up

After discharge from Visit 1 (post-natal hospitalization) and prior to Visit 2 (18-month follow-up), a follow-up phone call/email will be made to verify current contact information at the child's 6-month, 12 month and 15-month birthday. In addition, brochures from the CHOP patient and family education manual about child development and tips for talking will be mailed at those intervals.

The Bayley Scales of Infant Development–III (BSID-III) is an individually administered instrument that assesses the developmental functioning of infants and young children between 1 month and 42 months of age. [68] It assesses development across five domains: cognition, language, motor, social-emotional and adaptive. The cognitive, language and motor composite will be used for this study. This is the most widely used standardized developmental test battery in medical and educational settings. The composite scores have a mean of 100 and a standard deviation of 15.

The Modified Checklist for Autism in Toddlers (M-CHAT) is a brief screening instrument that was designed to detect autism in very young children. It consists of 23 yes/no items that pertain to social relatedness and communication. [69] These items were found to have the best discrimination between children diagnosed with and without autism/pervasive developmental disorder (PDD). It is an easy questionnaire for parents to complete because of its brevity and the clarity of the items. Those children who reach the cutoff should be referred for further evaluation of autism.

The Infant-Toddler Social and Emotional Assessment (ITSEA) is an empirically validated tool for identifying social-emotional problems and competencies that may be areas of concern in children 12 months to 35 months 30 days old.[87] It yields standard t-scores and percentiles in four domains with subscales and item clusters as follows: externalizing-activity/impulsivity, aggression/defiance and peer aggression, internalizing – depression/withdrawal, general anxiety, separation distress and inhibition to novelty, dysregulation-negative emotionality, sleep, eating, sensory sensitivity and Competence-compliance, attention, mastery motivation, imitation/play, empathy and prosocial peer relations. Item clusters include maladaptive, social relatedness and atypical. The ITSEA

was standardized and normed based on a nationally representative sample that was stratified to match the 2002 US census.

Medical Follow-up will include obtaining growth measurements and an interim medical history followed by performance of a physical and neurological examination by a team of developmental pediatricians and a nurse practitioner.

5.3 Pharmacokinetic Evaluation

Not applicable.

5.4 Safety Evaluation

Subject safety will be monitored by adverse events, vital signs, physical examinations, and laboratory data. After randomization, participants will be instructed to contact the Investigator if any symptoms or side effects develop. In case of any events, the Investigator will initiate the appropriate treatment according to their medical judgment. Subject diaries will also be reviewed at prenatal visits for any symptom or side effects. Recommendations for clinical intervention will be provided in the event of any unanticipated finding on brain MRI.

Recommendations for clinical intervention will be provided to any family whose child who demonstrates a developmental delay or potential genetic abnormality at the 18-month Developmental Follow-Up.

6 STATISTICAL CONSIDERATIONS

6.1 Primary Endpoint

The primary endpoint is the Motor Scale of the Bayley-III at 18 months of age.

6.2 Secondary Endpoints

Secondary endpoints will include the following:

1. Cognitive and Language Scales of the Bayley-III at 18 months of age
2. Total maturation score (TMS)
3. Volumes of whole brain, white matter, gray matter, and myelinated white matter (VMWM)
4. Presence of PVL on early post-natal brain MRI (categorical y/n)
5. Presence of PVL on early post-operative brain MRI (categorical y/n)
6. PVL Volume on early post-natal brain MRI
7. PVL Volume on early post-operative brain MRI
8. Compliance with progesterone regimen
9. Compliance with pre- and post-natal MRI's
10. Failure to return for 18-month neurodevelopmental evaluation.

6.3 Statistical Methods

6.3.1 Baseline Data

Baseline and demographic characteristics will be summarized by standard descriptive summaries (e.g. means or medians and standard deviations or interquartile range for continuous variables such as age and percentages for categorical variables such as gender).

6.3.2 Efficacy Analysis

The primary analysis will be intent-to-treat and will estimate differences between group means and their associated confidence intervals. In parallel we will test the null hypothesis that treatment has no effect on each of three outcomes. Because of its clinical relevance, the study is primarily designed to provide an estimate of possible effect sizes for the Bayley-III Motor Scale at 18 months. Important information will also be provided on effect sizes for the Bayley-III Cognitive and Language Scales at 18 months, as well as total maturation score (TMS) and volume of myelinated white matter (VMWM). The analysis will use a two-sided two-sample T-interval (estimation) or T-test (hypothesis testing). The confidence level will be set at 90% and the Type I error at 10%. Normality will be assessed and a transformation, or alternatively a non-parametric test, will be used if needed.

Based on our earlier work, the occurrence of PVL, both pre- and post-operatively, has a complex distribution. At both time points, when occurrence of PVL is considered a categorical outcome, there are patients with no evidence of PVL (PVL=0), as well as patients with varying amounts of PVL (PVL=1). When occurrence of PVL is considered as a continuous variable (volume of PVL), there is a mixture distribution with a peak at zero and the remainder of patients demonstrating a continuous highly skewed distribution. Thus, it is of interest to know whether the prevalence of PVL (categorical outcome) prior to surgery and post-surgery differs between treatment groups (intention to treat). It will also be of interest to know if the volume of PVL (continuous outcome), among those who have PVL at baseline, differs between treatment groups, both at baseline and post-operatively.

In the secondary analyses, we will use an exact binomial confidence interval to estimate differences between groups in the percent of subjects with PVL pre-operatively and the rate of increase post-operatively. The null hypothesis that treatment does not reduce the frequency of occurrence of PVL pre-operatively and the rate of increase post-operatively will be tested using Fisher's Exact test. The confidence level will be set at 90% and the Type I error at 10%. The PVL data can also be modeled in a more unified approach using mixture models, where the categorical data for patients with no PVL, and the continuous data for patients with evidence of PVL are modeled jointly.[88] In addition to the these analyses, we will describe compliance, both with treatment and with MRI tests, as well as determine loss to follow-up for the 18 month time point. We will secondarily model TMS, VMWM and the Bayley-III Cognitive and Language Scales as a function of treatment, as well as other potential predictors of fetal brain maturation and development including: gestational age, type of CHD, ethnicity, age at surgery, operative management variables, maternal education, and socioeconomic status. Presence of 22q11 deletions or other identified genetic anomaly and apo-lipoprotein E (*APOE*) genotype will be included as covariates in the analysis of the Bayley-III scores.

6.3.3 Safety Analysis

All subjects entered into the study at will be included in the safety analysis. The frequencies of AE by type, body system, severity and relationship to study drug will be summarized for both the mother and the neonate. Serious adverse events (SAE), if any, will be described in detail.

AE incidence will be summarized along with the corresponding exact binomial 95% two-sided confidence intervals.

6.4 Sample Size and Power

Based on the formal statistical considerations described below, we plan to enroll sufficient patients to have 80 evaluable patients (40 per treatment group) at the 18-month

neurodevelopmental testing. Based on known clinical outcomes for this patient population and experience with other studies with similar follow-up plans, we estimate a 5% early mortality and a further 20% attrition from all causes between hospital discharge and the 18-month evaluation. There may also be dropout prior to birth for a variety of reasons: fetal death, intolerance of therapy, withdrawal from study. We do not have accurate data to estimate this loss. We anticipate needing to enroll 110-130 patients to have a sufficient cohort at 18 months (40 per treatment group); with 60 to 52-56 patients per treatment group available for the post-natal and post-operative brain MRIs respectively.

The primary goal is to provide sufficiently precise estimates of the mean difference in the 18-month Bayley-III Motor Scale to inform the design of a future pivotal trial**.** Secondarily we will provide estimates of differences in the TMS, VMWM, and the Bayley-III Cognitive and Language Scales. All primary analyses will be performed on an intention-to-treat (ITT) basis.

Table 1: Expected width of confidence intervals for continuous outcomes for several possible sample sizes			
N per group	Distance from Mean Difference to Limits of 90% CI based on T-interval		
	**SD**	**TMS***	**Bayley Score****
40	±0.372	±0.49	**±5.6**
50	±0.332	**±0.40**	±5.0
*based on a postulated SD=1.32			
**based on a postulated SD=15 for a normalized Bayley-III Motor Scale (mean=100)			

Table 1 shows that for the Bayley-III Motor Scale, assuming 40 evaluable subjects per treatment group, and an SD of 15, the ½ width of a two-sided 90% confidence interval on the mean difference is expected to be 5.6. Thus, for example, if the mean difference is 6.0 units, a clinically relevant value for this population, the 90% two-sided confidence interval is expected to be on the order of (0.4, 11.6), suggesting that while only a minor effect cannot be ruled out, important positive effects of progesterone appear possible. In contrast, if there is no effect of progesterone, we expect an interval centered around zero with i.e. (-5.6, 5.6). This initial study will thus provide important information about the magnitude of the effect of progesterone, and an indication of whether it is worthwhile to proceed with further trials. For TMS, results from Licht (personal communication) estimated the mean (median) value of TMS in a similar cohort to be 10.1 (10.0) with a standard deviation (SD) of 1.06 and range of 8.0 to 13.5. Conservatively assuming an SD that is 25% larger than that in the pilot data (SD=1.32), Table 1 shows that, with a sample size of 60 per group, two-sided 90% confidence intervals for the mean difference in TMS are anticipated to have a ½ width on the order of ±0.44. Thus, for TMS, if the mean difference is on the order of 2.0, the 90% confidence interval would be (1.56, 2.44). Estimated SD's for VMWM are not yet available.

Table 2: Expected endpoints of 90% confidence interval (CI) for different scenarios for PVL. Calculations are based on Pearson's Chi-square Method for n=60 (pre-surgical) or, conservatively) n=52 (post-surgical) per group.			
Proportion in Control Group	Proportion in Treated Group	True Difference in Proportions	Expected Endpoints of 90% CI

Post-natal PVL (pre-surgical, n=60)			
19.0%	8.5%	10.5%	**(0.3%, 20.7%)**
19.0%	19.0%	0%	**(-11.8%, 11.8%)**
Post-operative increase in PVL from baseline (n=52)*			
38.0%	24.0%	14%	**(-0.8%, 28.8%)**
38.0%	38%	0%	**(-14.6%, 14.6%) (-15.7%, 15.7%)**
***Conservatively as we expect n=52 to 56 post-operatively**			

Next, we consider the categorical outcomes. In pilot data (Licht, personal communication), 19% of subjects had some PVL prior to surgery and 38% of infants increased the level of PVL post-surgery. As part of our secondary aims, the pilot study is intended to estimate the differences in these outcomes between groups. To recruit 40 evaluable subjects at 18 months we anticipate having 60 patients available pre-surgery (post-natal) and 52 to 56 after surgery. Table 2 shows that if PVL at baseline is 19% in the control group, and the true effect is 10.5%, then the endpoints of the 90% CI are (0.3%, 20.7%)This would suggest that while we could not rule out essentially a null effect of progesterone, the evidence would suggest a possible substantial effect. In contrast if there were no effect, we expect the confidence interval to be centered near zero with endpoints of ±11.8%%. Similarly, if the post-operative increase in PVL is 38% in the control group, and the true effect size is 14%, then the 90% confidence interval is expected to have endpoints of the 90% CI are (-0.7%, 28.7%). Again, while we could not rule out essentially a null effect of progesterone, the evidence would suggest a possible substantial effect. In contrast if there were no effect, we expect the confidence interval to be centered near zero with endpoints of ±15.7%%.

6.5 Interim Analysis

No interim analysis is planned for this Phase II study.

7 STUDY MEDICATION (STUDY DEVICE OR OTHER STUDY INTERVENTION)

7.1 Description

Crinone® (progesterone gel) is a bioadhesive vaginal gel containing micronized progesterone in an emulsion system, which is contained in single use, polypropylene vaginal applicators. The carrier vehicle is an oil-in-water emulsion containing the water swellable, but insoluble polymer, polycarbophil. The progesterone is partially soluble in both the oil and water phase of the vehicle, with the majority of the progesterone existing as a suspension. Physically, Crinone has the appearance of a soft, white to off-white gel.

Replens is a hormone-free vaginal moisturizer. Ingredients include purified water, glycerin, mineral oil, polycarbophil, carbomer homopolymer type B, hydrogenated palm oil, glyceride, sorbic acid, and sodium hydroxide. This product will act as the placebo in this study.

7.1.1 Packaging

Crinone is supplied in a single use, disposable, white polypropylene vaginal applicator with a teal twist-off cap. Each applicator delivers 1.125 grams of Crinone gel containing 90 mg (8% gel) of progesterone in a base containing glycerin, mineral oil, polycarbophil,

carbomer 934P, hydrogenated palm oil glyceride, sorbic acid, purified water and may contain sodium hydroxide.

Replens Long-Lasting Moisturizer is supplied in pre-filled, sealed and individually wrapped applicators.

Both Crinone 8% and Replens will be obtained from commercial sources and stored according to package labeling. The investigational pharmacy will dispense the study medications in a blinded fashion by removing any commercial labeling that contains the identity of the product. The study medications will be dispensed as a 30 +/- 15-day supply. Occasionally the timing of prenatal visits to CHOP may require variation in the amount of drug supplied to a mother at one time.

7.1.2 Labeling

Study drug labeling will be in compliance with applicable local and national regulations

7.1.3 Dosing

Crinone 8% is administered vaginally at a dose of 90 mg twice daily. The rounded tip of the applicator is inserted into the vagina. After insertion, the plunger is pushed to release the gel into the vagina. The applicator is removed.

Replens Long-Lasting Moisturizer will also be dosed at one applicator intravaginally twice daily.

7.1.4 Treatment Compliance and Adherence

The Investigational Pharmacy will keep an Investigational Drug Accountability Record for the medication and placebo, which keeps track of study drug that is received, dispensed, and returned. Subjects will be asked to maintain a diary documenting each drug application. Compliance with the treatment protocol will be assessed at each visit. The subject diary will be reviewed. Patients will be asked to return all unused medication packages. Unused study drug will be returned to the Investigational Pharmacy to count the remaining drug to assess compliance. Subjects will not be terminated from the study form non-compliance. However, the compliance protocol will be considered in secondary analyses to determine if there is any difference in effect between compliers and non-compliers.

7.1.5 Drug Accountability

The Investigational Pharmacy will keep an Investigational Drug Accountability Record for the medication and placebo, which keeps track of anything that is received, dispensed, and returned.

8SAFETY MANAGEMENT

8.1 Clinical Adverse Events

Clinical AEs will be monitored throughout the study.

8.2 Adverse Event Reporting

Unanticipated problems related to the research involving risks to subjects or others that occur during the course of this study (including SAEs) will be reported to the IRB in accordance with CHOP IRB SOP 408: Unanticipated Problems Involving Risks to Subjects. AEs that are not serious but that are notable and could involve risks to subjects will be summarized in narrative or other format and submitted to the IRB at the time of continuing review.

8.3 Definition of an Adverse Event

An adverse event is any untoward medical occurrence in a subject who has received an intervention (drug, biologic, or other intervention). The occurrence does not necessarily have to have a causal relationship with the treatment. An AE can therefore be any unfavorable or unintended sign (including an abnormal laboratory finding, for example), symptom, or disease temporally associated with the use of a medicinal product, whether or not considered related to the medicinal product.

All AEs (including SAEs) will be noted in the study records and on the case report form with a full description including the nature, date and time of onset, determination of non-serious versus serious, intensity (mild, moderate, severe), duration, causality, and outcome of the event.

8.4 Definition of a Serious Adverse Event (SAE)

An SAE is any adverse drug experience occurring at any dose that results in any of the following outcomes:

- death,
- a life-threatening event (at risk of death at the time of the event),
- requires inpatient hospitalization or prolongation of existing hospitalization,
- a persistent or significant disability/incapacity, or
- a congenital anomaly/birth defect in the offspring of a subject.

Important medical events that may not result in death, be life-threatening, or require hospitalization may be considered a serious adverse drug event when, based upon appropriate medical judgment, they may jeopardize the subject and may require medical or surgical intervention to prevent one of the outcomes listed in this definition.

A distinction should be drawn between serious and severe AEs. A severe AE is a major event of its type. A severe AE does not necessarily need to be considered serious. For example, nausea which persists for several hours may be considered severe nausea, but would not be an SAE. On the other hand, a stroke that results in only a limited degree of disability may be considered a mild stroke, but would be an SAE.

The Common Terminology Criteria for Adverse Events (CTCAE) Version 4.0 (http://ctep.cancer.gov) provides a grading system that is used to categorize the severity of adverse events, as follows:

Grade	Severity	Definition
1	Mild	Asymptomatic or mild symptoms; clinical or diagnostic observations only; intervention not indicated.
2	Moderate	Minimal, local, or noninvasive intervention indicated; limiting age-appropriate instrumental activities of daily living (ADL).
3	Severe	Severe or medically significant but not immediately life-threatening; hospitalization or prolongation of hospitalization indicated; disabling; limiting self-care ADL.
4	Life-threatening	Life-threatening consequences; urgent intervention indicated.

5	Death	Death related to AE

A SAE, as defined above, encompasses CTCAE grades 4 and 5, and any Grade 3 event that requires or prolongs hospitalization, or that substantially disrupts the ability of the subject to conduct normal life functions.

Expectedness

The purpose of reporting is to provide new, important information on serious reactions or events previously unobserved or undocumented. Therefore, all AEs will be evaluated as to whether their occurrence was unexpected, using the following definitions:

•*Unexpected*: An unexpected AE or adverse reaction is one for which the nature or severity is not consistent with information in the protocol, consent form, or product brochure. An AE or adverse reaction also may be categorized as unexpected if the event has not previously been observed at the same specificity and/or severity.

•*Expected*: An event is considered expected if it is known to be associated with the study drug(s) and/or the disease state.

Pregnant subjects: The standard definition for SAE will be used (Section 7.4).

However, the following adverse events will not be classified an SAE for the purposes of this study as they are expected events associated with pregnancy and delivery:

a) Hospitalizations for treatment planned prior to randomization
b) Hospitalizations for elective treatment of a pre-existing condition
c) Miscarriage
d) Preterm labor / suspected preterm labor
e) Premature rupture of membranes (PROM) / suspected PROM
f) Preterm delivery
g) Preterm delivery in maternal interest
h) Preterm delivery in fetal interest
i) Hospitalization for pregnancy induced hypertension
j) Hospitalization for "maternal discomfort"
k) Hospitalization for "rest"
l) Hospitalization for "observation" or "monitoring" for which the women are admitted for a period of less than 12 hours.

Newborn subjects: It is important to note at baseline these newborns are medically fragile pediatric patients requiring high-risk neonatal cardiothoracic surgery. This puts them at risk for many expected adverse events and serious adverse events without any additional interventions. In response to a surgical randomized clinical trial (RCT) run by the Pediatric Heart Network at the NIH, a novel adverse event reporting system was developed for this medically fragile population.[89] The system is based on the concept of the identification of **"sentinel" SAEs**. For this study, these events will be

a. death
b. listing for cardiac transplantation
c. endocarditis
d. thromboembolic event
e. need for surgical or catheter-based intervention
f. heart block
g. hemolysis.

The remaining adverse events will be organized in a code list by body system. We have used this system of sentinel SAE reporting with another RCT study Protocol No 10-007750.

8.4.1 Relationship of SAE to Study Drug

The relationship of each SAE to the study intervention should be characterized using one of the following terms in accordance with CHOP IRB Guidelines: definitely, probably, possibly, unlikely, or unrelated.

Type of Unanticipated Problem	Initial Notification (Phone, Email, Fax)	Written Report
Internal (on-site) SAEs Death or Life Threatening	24 hours	Within 2 calendar days
Internal (on-site) SAEs All other SAEs	7 days	Within 7 business days
Unanticipated Problems Related to Research	7 days	Within 7 business days
All other AEs	N/A	Brief Summary of important AEs may be reported at time of continuing review

8.5 IRB Notification of SAEs and Other Unanticipated Problems

The Investigator will promptly notify the IRB of all on-site unanticipated, serious Adverse Events that are related to the research activity. Other unanticipated problems related to the research involving risk to subjects or others will also be reported promptly. Written reports will be filed using the eIRB system and in accordance with the timeline below. External SAEs that are both unexpected and related to the study intervention will be reported promptly after the investigator receives the report.

8.5.1 Follow-up report

If an SAE has not resolved at the time of the initial report and new information arises that changes the investigator's assessment of the event, a follow-up report including all relevant new or reassessed information (e.g., concomitant medication, medical history) should be submitted to the IRB. The investigator is responsible for ensuring that all SAE are followed until either resolved or stable.

8.6 Investigator Reporting of a Serious Adverse Event to Sponsor

Not applicable.

8.7 Medical Emergencies

These subjects are either pregnant mothers or newborns undergoing cardiac surgery requiring postoperative cardiac intensive care. Maternal Fetal Medicine professionals in the Center for Diagnosis and Treatment will be responsible for the care of pregnant subjects in the event of medical emergencies. Cardiac surgical, medical, anesthesia, and nursing professionals in the Cardiac Center will be responsible for the care and treatment of these infants in the event of medical emergencies.

9STUDY ADMINISTRATION

9.1 Treatment Assignment Methods

9.1.1Randomization

The study biostatistician, Dr. Putt, will generate a block randomization schedule based on stratification by fetal CHD diagnosis: TGA, HLHS, and other CHD. The randomization schedule will be based on equal allocation of subjects to the progesterone and placebo arms within each block. The schedule will be generated using variable block sizes. Upon confirmation of eligibility in the DMS, the randomization module will be programmed to generate a randomization number that corresponds to a unique kit number of progesterone or placebo treatment. This kit number will be used to distribute treatment.

8.1.2 Blinding

The study biostatistician, Dr. Putt, will generate a block randomization schedule based on stratification by fetal CHD diagnosis: TGA, HLHS, and other CHD. The randomization schedule will be based on equal allocation of subjects to the progesterone and placebo arms within each block. The schedule will be designed so that the block size is not fixed. Upon confirmation of eligibility in the DMS, the randomization module will be programmed to generate a randomization number that corresponds to a unique kit number of progesterone or placebo treatment. This kit number will be used to distribute treatment.

9.1.3 Unblinding

Unblinding can occur if the PI instructs the Investigational Pharmacy to reveal this information. A direct request from the PI to Investigational Pharmacy must be documented.

9.2 Data Collection and Management

The following non-research data sources will be queried for demographic and medical data etc.: ChartMax, CardioIMS, EPIC, CardioAccess, CompuRecord, PC4 and other applicable clinical databases.

1. Confidentiality: The following methods will be utilized to ensure confidentiality of research study data [including source documents, case report forms, & data in computerized systems]
    a. Access to identifiable study data, databases containing identifiers, and systems will be limited to study personnel who have valid CHOP enterprise password and login, who have been designated by the PI and approved by the IRB, and who are using a computer that is connected to the internal CHOP network.
    b. Password-protected data base will be utilized
    c. Data files copied to removable disk drives will be encrypted
2. Security: Data will be entered, cleaned, and maintained in a password-protected research database in the located in the Cardiothoracic Surgery suite. All data management applications, databases, and data are hosted on secure servers in the offsite CHOP data center, hosted by a commercial provider. Physical access to the data center is monitored, logged, controlled, and limited to approved CHOP personnel. System administration and support is provided by CHOP Research Information Systems. Servers and the Case Report Application are actively monitored 24x7x365 and replicated between two geographically separate fully redundant data centers. Server backup at the file system level is performed multiple

times per day, and a multi-day real-time accessible rolling archive will be maintained. After seven days, data will be compressed and moved to an online archive that may be accessed upon documented request to Research Information Systems. System and data recovery are accomplished through documented standard operating procedures.

3. Anonymization, de-identification, or destruction: Identifiers will be kept in conjunction with the study identification number for the purposes of the18 month follow-up and future investigation.

9.3 Confidentiality

All data and records generated during this study will be kept confidential in accordance with Institutional policies and HIPAA on subject privacy and that the Investigator and other site personnel will not use such data and records for any purpose other than conducting the study. Safeguards are described under Data Collection and Management (9.2).

9.4 Regulatory and Ethical Considerations

9.4.1 Data and Safety Monitoring Plan

The purpose of this Data and Safety Monitoring Plan is to ensure the safety of research participants and the integrity of the research data.

Safety of Research Participant:

Protection of the rights and welfare of subjects and their families will be maintained during the recruitment, consenting process, and study participation. Dr. Gaynor and his study team have over a 15-year experience in recruiting and enrolling patients for research studies in the Cardiac Center. Subject privacy and confidentiality will be maintained as described in Section 8.3. All research responsibilities delegated by the principal investigator to study team members will be carried out in accordance with the protocol, federal regulations, federal, state, and local laws and institutional policies and procedures. The study team has two research nurses, one of which is a Certified Clinical Research Coordinator (CCRC). If any the results of research procedures appear to be important for immediate clinical care, these results will be provided to the Principal Investigator (Dr. Gaynor) of the study who will forward the information to the subject's surgeon, cardiologist and/or primary physician.

The Principal Investigator (Dr. Gaynor) and Co-Investigators (Drs. Rychik, Szwast, Gebb, and Licht,) will be responsible for reviewing subject enrollment data, maternal events, and clinical event data as described in Section 7.4 every 2 weeks. Dr. Gaynor is a pediatric cardiothoracic surgeon. Dr. Licht is a pediatric neurologist. Drs. Rychik and Szwast are fetal cardiologists. Dr. Gebb is an expert in Maternal-Fetal Medicine. Data will be reviewed for adverse events and actions appropriate to the event will be initiated.

Data Safety Monitoring Board (DSMB):

An independent Data Safety Monitoring Board has been established. The members have expertise in maternal-fetal medicine, pediatric neurology, pediatric critical care, peri-operative care of the neonate with CHD, and neurodevelopmental outcomes for patients with CHD. The DSMB will review the protocol with respect to ethical and safety standards and making recommendations as necessary. This committee will monitor the emerging results for safety and efficacy. The DSMB will also review the performance of the trial in terms of recruitment, protocol adherence and data quality. The interval and timing of DSMB

meetings after the initial review of the protocol will be at the discretion of the committee itself.

DSMB Membership:

Jane Newburger, Pediatric Cardiology, Boston Children's Hospital (Chair)

Dean Andropoulos, Pediatric Cardiac Anesthesia, Texas Children's Hospital

Richard Ittenbach, Biostatistician, Cincinnati Children's Medical Center

Patrick McQuillen, Pediatric Cardiac Critical Care Medicine, University of California, San Francisco

Steven Miller, Pediatric Neurology, Hospital for Sick Kids, Toronto

David Stamilo, Maternal-Fetal Medicine, Wake Forest Baptist Health

9.4.2 Risk Assessment

Progesterone Therapy

The existing evidence suggests that progesterone therapy is associated with potential benefits to the mother and fetus in singleton pregnancies, with greater than minimal risk.

Maternal Risks

Multiple reviews have found that there do not appear to be any significant maternal or fetal risks associated with progestin therapy. , ,[40, 42, 43, 45] The lower blood levels after vaginal administration have been associated with a lower prevalence of systemic side effects.[3, 5] The most common side effect of vaginal progesterone administration is local irritation. A recent randomized trial of Crinone vaginal gel vs. intramuscular progesterone during ART showed that patient satisfaction in terms of ease of administration, convenience of administration and discomfort was significantly greater for patients receiving Crinone.[90] Additional supportive evidence of safety and efficacy is found in the 2011 FDA Advisory Committee Briefing Book. (Attached to application)

Stopping Rule

In previous studies, the vaginal progesterone gel has been well tolerated with few adverse effects. The most common effect is vaginal irritation which will be treated with sitz baths; and for severe cases a low potency topical steroid cream (hydrocortisone 1 % cream). If a woman goes into preterm labor, this will be treated according to standard clinical protocols. We anticipate that need to terminate study drug prior to 39 weeks or delivery (if earlier) will be infrequent.

Risks to Fetus and Newborn

The effects of progesterone on perinatal outcomes and the neonate are of special interest. A recent meta-analysis attempted to quantify the effect of progesterone therapy for the prevention of preterm birth on perinatal outcomes.[42] The authors concluded that progesterone administration in singleton pregnancies at risk for preterm birth improves perinatal outcomes, but may actually have adverse effects in multiple pregnancies.[42] Other reviews have also found that there do not appear to be any significant maternal or fetal risks associated with progestin therapy.[45] Longer term outcomes have also been evaluated.[36] Surviving children from a trial of 17-OHP-C vs. placebo to prevent preterm birth underwent physical examination and developmental screening at 48 months of age.

Progesterone therapy was not associated with any adverse physical or neurodevelopmental sequelae.[36]

Risk Assessment for Pre-Natal Evaluations

Fetal Magnetic Resonance Imaging:

All of the fetal brain MRI examinations performed in this study will be during the 25th week GA or beyond. No MRI will be performed in the first half of pregnancy or embryonic period. None of the studies will be performed with gadolinium contrast administration. None of the studies will be done with sedation or general anesthesia. General MRI safety guidelines for metallic objects in the MRI environment will be monitored as part of standard practice by the MRI technologists and radiology nursing staff. At the Children's Hospital of Philadelphia, clinical fetal MRI studies are routinely and safely performed at 16 weeks GA and beyond. To avoid conflict of interest concerning decisions about termination or continuation of a study, the anesthesiologist for the research MRIs will not be a member of the study team.

There are no verified or scientifically proven additional side effects of clinical MRI on human fetuses within the safety limits of performing MRI studies. The FDA has specific limits on the amount of energy deposition by MRI which is built in as a fail-safe warning mechanism on all of the MRI systems at CHOP, which used for all patients. There is little direct scientific evidence of harmful effects with short-term exposure to high static magnetic fields.[91] Two to three decades of practice generally supports this widely-held belief. [78, 79] FDA guidelines now classify MRI devices as "nonsignificant risk" when neonates (infants less than one month of age) are scanned at a static magnetic field of 4T or less. As a result, it has become widespread routine clinical practice to scan even the youngest premature infants who are stable for transport. Myers and coworkers reported no significant reduction in fetal growth in 74 volunteer subjects exposed in utero to MRI vs. matched controls.[92] Also the recent practice parameter from the American College of Radiology and the Society for Pediatric Radiology for fetal MRI states, "At this stage, the preponderance of research studies have failed to discover any reproducible harmful effects of exposure of the mother or developing fetus to the 3T or weaker magnetic fields used in the routine clinical MR imaging process."[93] Some MRI sequences are for research purposes and are not FDA approved. However, all the sequences will be below the FDA energy deposition limits and no gadolinium contrast will be used.

A "potential" side effect of the MRI environment is exposure to high sound levels. Baker and associates reported no demonstrable increase in disease, disability, or hearing loss in 20 children examined in utero using echoplanar MRI for suspected fetal compromise.[94] In a 2010 study, Reeves and colleagues found no evidence that exposure of the fetus to 1.5T MR imaging during the second and third trimesters is associated with an increased risk of substantial neonatal hearing impairment. [95]

Risk Assessment for Post-Natal Evaluations

Transport:

This is a high-risk group of subjects being transported from the cardiac intensive care unit (CICU) to the MRI suite. The risks of transport include exposure to cooling temperatures, dislodging arterial and venous lines and accidental extubation. To accomplish safe transport of subjects, all subject transports will be performed by a team of cardiac anesthesiologists including an attending, a fellow and a respiratory therapist. This team of physicians has a great deal of experience both in transporting high-risk neonates (n>200) to the MRI suite for preoperative and post-operative studies.

Magnetic Resonance Imaging:

MRI is an electromagnetic imaging technique that does not use ionizing radiation such as x-rays to obtain the images; as such there are minimal risks involved with this technique. The Food and Drug Administration (FDA) guideline for average specific absorption ratio (SAR) is 3W/kg over the head for 10min. The SAR level of this CASL sequence, calculated using quasi-static approximation (spherical head model), is 1W/kg on an average head size with radius of 10cm. The SAR level is also automatically calculated and monitored by integrated product program during MR scanning according to the legal requirement value (IEC 60601-2-33, accepted by the US FDA). A mean SAR level (percent of maximal allowable SAR) of 54.5±15.6% (40-80%) was observed on 13 healthy adult subjects (radiology in press paper). The SAR level actually is lower in children and infants than in adults because it is roughly proportional to the square of the head size (radius).[96] The 3T CASL method is therefore very safe for pediatric imaging.

Sedation for the Pre-Operative MRI:

The pre-operative MRI will be performed during the same anesthetic period as the planned surgery. On the morning of heart surgery, the patient will be induced for anesthesia using published protocols.[66, 97] Immediately prior to surgery, the patient will be brought to the MRI scanner located between the CICU and the operating rooms. Upon completion of the pre-operative MRI protocol, the infant will be transported to the operating room where the surgery will take place. During transport and the MRI procedure, the patient will be monitored by a team of cardiac anesthesiologist. To avoid conflict of interest concerning decisions about termination or continuation of a study, the anesthesiologist for the research MRIs will not be a member of the study team.

Sedation for the Post-Operative MRI:

For the post-operative MRI procedure, primary consideration will be given toward the possibility of a non-sedated, “sated sleep” protocol. All infants will be transported to and from the MRI scanner by a team of pediatric cardiac anesthesiologists. For a sated-sleep MRI the infant would be held NPO for 3 or 4 hours prior to the study. The subject would be transported awake to the scanner, ear protectors and ECG leads would be placed and the subject swaddled with warm blankets. The infant would then be offered formula/breast milk ad-lib or as recommended by the ICU protocol. When postprandial sleep is achieved the subject is placed in the scanner. The MRI sequences will be run in order of relative importance to the study, with T1 MPR and DWI taking highest priority.

The decision to use the sated sleep protocol will be made by either Dr. Susan Nicolson or Dr. Lisa Montenegro and it will be done on a patient-by-patient basis. There are some concerns that may make this procedure untenable: first, the patient must tolerate oral feeds to be eligible for such a protocol; second, agitation in a post-operative infant may cause unsafe increase in blood pressure. The advantages are clear and include primarily decreased risk of respiratory compromise when compared to sedation.

If a “sated sleep” MRI procedure is not an option (if the infant is not tolerating oral feeds, for instance), an individualized sedation protocol will be administered by Cardiac Anesthesia for the post-operative MRI scan. The sedation will follow protocols that have established safety and have been used successfully in the past. The medications used for sedation will be determined by the anesthesiologist responsible for the case but will likely include the use of pentobarbital. All subjects will have heart rate, blood pressure and oxygen saturations monitored throughout the study. Any deviation in oxygen saturations of

more than 10% would necessitate the discontinuation of the study and immediate medical evaluation of the subject.

Stopping Rule

Our previous experiences with pre-operative and post-operative MRI suggest that these are safe procedures with our research team. In this study we expect similar results. This notwithstanding, enrollment for this study will be halted temporarily if two subjects experience vital sign fluctuations that require termination of their MRI. Enrollment will not resume until independent review of procedures determines the safety for continuation. All adverse events will promptly be reported to the IRB. All SAEs will be reported to the board within 24 hours of occurrence

Review of Medical Record

Collecting data from the medical record may increase the risk of breach of privacy and confidentiality. All data and records generated during this study will be kept confidential in accordance with institutional policies and HIPAA on subject privacy and the Investigator and other site personnel will not use such data and records for any purpose other than conducting the study.

Blood Work for Mothers/Fathers

Taking blood may cause some pain, bleeding or bruising at the spot where the needle enters your body. Rarely, taking blood may cause fainting or infection.

Blood Work for Infant

The sample will be obtained from an existing central venous or arterial line, which is associated with minimal risk of pain or complication.

18-month Developmental and Medical Follow-Up

There are no physical risks but toddler-age children may become uncooperative. The Neonatal follow-up team has over 10 years of experience in developmental assessments of infants and children with CHD. Parents and child will remain together throughout this assessment in order to provide support and comfort to the child. Because the early assessment measures for children from birth to 3 are based on play, typically children enjoy these sessions.

9.4.3 Potential Benefits of Trial Participation

There is a significant risk of preterm birth for women carrying a fetus with CHD. A potential benefit to the mother and child is prolongation of the pregnancy with a reduction in perinatal mortality and morbidity.

There is a significant risk of brain injury and subsequent neurobehavioral disability for the newborn undergoing cardiac surgery. A potential benefit of the study is a reduction in prenatal and perioperative brain injury resulting in improved neurobehavioral outcomes.

The possibility of collateral benefit from participation in the post-operative MRI has been demonstrated in a related study. Over the course of this study, entitled “Hemodynamic Neuroimaging” (IRB # 4357), 7.4% of participants (2/27) from the same target population have benefitted directly from a post-operative brain MRI; including one patient whose MRI revealed an incidental finding of a pre-symptomatic hyperacute non-occlusive sino-venous thrombus of both transverse sinuses, also involving the torcula. This condition is life-threatening, and the post-operative MRI allowed physicians to intervene early with both primary and secondary prevention. The other patient’s MRI revealed an arterial ischemic

stroke, which allowed for early secondary prevention. For all subjects in the present study, Dr. Licht will meet with the parents to review all the imaging data. The significance of injury, if present, will be explained to the parents in layman language and interventional therapies will be put in place if they are deemed necessary.

Current data demonstrates that some degree of neurobehavioral disability is present on over 50% of survivors of cardiac surgery in the newborn period. Indeed, neurodevelopmental disability is now recognized as the most common complication of critical CHD (i.e. those patients requiring cardiac surgery in infancy) and has the most negative impact on quality of life, academic performance, and opportunity for independence as an adult. Regardless of their origin, neurodevelopmental problems adversely affect learning and the attainment of academic, social, and vocational skills. Ultimately, they undermine mental health and employment opportunities in adulthood. There is a wealth of disturbing data documenting the impact of neurodevelopmental deficits among children and adults affected by CHD. The need for special and rehabilitative services results in significant costs to the patients' families and to society. A potential benefit of the study is a reduced incidence of neurobehavioral disability leading to lower health care costs for society.

9.4.4 Risk-Benefit Assessment

Risk-Benefit Ratio:

There are significant potential benefits to the mother and fetus including prolongation of pregnancy, and improved brain development, with a reduction in peri-operative white injury leading to better long-term neurodevelopmental outcomes. Previous studies indicate that maternal progesterone therapy during the third trimester is associated with only a minor increase above minimal risk.

As outlined above, there is a possibility of collateral benefit from participation in the MRI assessments, however because of the potential need for sedation, this is a greater than minimal risk study with prospect for collateral benefit (Subpart D category *§46.406* or *§50.53*).

9.5 Recruitment Strategy (or Case Ascertainment)

Subjects will be recruited from the Cardiac Center at CHOP. Eligible mothers and with a fetus with a prenatal diagnosis of CHD, who are followed by the Fetal Heart Program, will be recruited in person during a prenatal visit.

We plan to enroll sufficient patients to have <u>80 evaluable patients (40 per treatment group) at the 18-month</u> neurodevelopmental testing. Based on known clinical outcomes for this patient population and experience with other studies with similar follow-up plans, we estimate a 5% early mortality and a further 20% attrition from all causes between hospital discharge and the 18-month evaluation. There may also be dropout prior to birth for a variety of reasons: fetal death, intolerance of therapy, withdrawal from study. We do not have accurate data to estimate this loss. We anticipate needing to enroll 110-130 patients to have a sufficient cohort at 18 months (40 per treatment group). We will use the IRB approved Cardiac Center Research Intro Sheet. This sheet is given to families in CICU and families followed by the Fetal Heart program at CHOP and lists <u>all</u> the studies in the Cardiac Center that recruit pre-operatively. [See current version attached in appendix section]. In addition, we plan to develop a study specific brochure similar to that used in the OPPTIMUM trial (attached in appendix), which we will submit for IRB approval.

9.6 Informed Consent/Assent and HIPAA Authorization

Dr. Gaynor and/or one of the research nurses will approach eligible families in person during a prenatal visit or upon admission to CHOP and ask their permission to review the research study. If families are willing, the following informed consent process will be initiated in order to assure subjects comprehend the purpose of the study, the study procedures, and the risk-benefit profile.

1. The research protocol will be reviewed verbally.
2. The consent form document will be reviewed.
3. The HIPAA authorization form will be reviewed.
4. Time will be provided for questions and decision making. There will be ongoing discussion with the parents to give them adequate time to consider participation.

Dr. Gaynor and/or one of the research nurses will approach eligible families in person during an outpatient visit or upon admission to CHOP and ask their permission to review the consent addendum. If families are willing, the following informed consent process will be initiated in order to assure subjects comprehend the purpose of the consent addendum, the additional study procedures, and the risk-benefit profile.

1. The research protocol will be reviewed verbally.
2. The consent addendum form document will be reviewed.
3. Time will be provided for questions and decision making.

9.7 Payment to Subjects/Families

9.7.1 Reimbursement for travel, parking, and meals

At the time of both fetal brain MRIs, mothers will be reimbursed for travel, parking, need for babysitting, and meals. This will average between $130 (local)-$900 (outside the tristate needing hotel and airfare) based on where mothers reside during study period.

At the 18-month follow-up families will be reimbursed for travel, parking, need for babysitting, and meals. We have budgeted $425-500 on average per family based on a range of $190 for families coming by car with no hotel stay (70% referral base), $600 for families coming by car with one night hotel stay or the need for round trip limo service (13% of referral base) to $1300 for families coming by air with one-two hotel night stay (17% of our referral base).

9.7.2 Payments to parent for time and inconvenience (i.e. compensation)

Upon completion of each fetal brain MRI each mother will be given a $50.00 gift card to compensate for time and inconvenience.

At the 18-month follow-up the parent will receive a $50.00 gift card to compensate for time and inconvenience.

9.7.3 Gifts

At the 18-month follow-up the child will receive a book or small toy as a token of appreciation.

10 PUBLICATION

Any publication or presentation of the data will be done in a de-identified manner and under the guidance of J. William Gaynor, Principal Investigator.

11 REFERENCES

1. Grow DR. Metabolism of endogenous and exogenous reproductive hormones. *Obstet Gynecol Clin North Am*. 2002;29:425-436
2. Levine H, Watson N. Comparison of the pharmacokinetics of crinone 8% administered vaginally versus prometrium administered orally in postmenopausal women(3). *Fertil Steril*. 2000;73:516-521
3. Cicinelli E, de Ziegler D, Bulletti C, Matteo MG, Schonauer LM, Galantino P. Direct transport of progesterone from vagina to uterus. *Obstet Gynecol*. 2000;95:403-406
4. De Ziegler D, Bulletti C, De Monstier B, Jaaskelainen AS. The first uterine pass effect. *Ann N Y Acad Sci*. 1997;828:291-299
5. Shantha SB-G, J.; Locke, J.P.; Warren, M.P. Natural vaginal progesterone is associated with minimal psychological side effetcs: A preliminary study. *j Women's Health Gender-Based Medicine*. 2001;10:991-997
6. Hemauer SJ, Yan R, Patrikeeva SL, Mattison DR, Hankins GD, Ahmed MS, Nanovskaya TN. Transplacental transfer and metabolism of 17-alpha-hydroxyprogesterone caproate. *Am J Obstet Gynecol*. 2008;199:169 e161-165
7. Quadros PS, Pfau JL, Wagner CK. Distribution of progesterone receptor immunoreactivity in the fetal and neonatal rat forebrain. *J Comp Neurol*. 2007;504:42-56
8. Jiang N, Chopp M, Stein D, Feit H. Progesterone is neuroprotective after transient middle cerebral artery occlusion in male rats. *Brain Res*. 1996;735:101-107
9. Singh M, Su, C. Progesterone and neuroprotection. *Hormones and Behavior*. 2013;63:284-290
10. Thomas AJ, Nockels RP, Pan HQ, Shaffrey CI, Chopp M. Progesterone is neuroprotective after acute experimental spinal cord trauma in rats. *Spine (Phila Pa 1976)*. 1999;24:2134-2138
11. Ibanez C, Shields SA, El-Etr M, Leonelli E, Magnaghi V, Li WW, Sim FJ, Baulieu EE, Melcangi RC, Schumacher M, Franklin RJ. Steroids and the reversal of age-associated changes in myelination and remyelination. *Prog Neurobiol*. 2003;71:49-56
12. Sayeed I, Stein DG. Progestorone as a neuroprotective factor in traumatic and ischemic brain injury. *Progress in Brain Research*. 2009;175
13. Stein DG. A clinical/translational perspective: Can a developmental hormone play a role in the treatment of traumatic brain injury? *Horm Behav*. 2013;63:291-300
14. Stein DG. Progesterone in the treatment of acute traumatic brain injury: A clinical perspective and update. *Neuroscience*. 2011;191:101-106
15. Doppenberg EM, Choi SC, Bullock R. Clinical trials in traumatic brain injury: Lessons for the future. *J Neurosurg Anesthesiol*. 2004;16:87-94
16. Ma J, Huang S, Qin S, You C. Progesterone for acute traumatic brain injury. *Cochrane Database Syst Rev*. 2012;10:CD008409

17. Stein DG, Hoffman, S.W. Estrogen and progesterone as neuroprotective agents in the treatment of acute brain injuries. *Pediatric Rehabilitation*. 2003;6:13-22

18. Baulieu EE. Neurosteriods: A new function in the brain. *Biol Cell*. 1991;71:3-10

19. Hirst JJ, Walker DW, Yawno T, Palliser HK. Stress in pregnancy: A role for neuroactive steroids in protecting the fetal and neonatal brain. *Dev Neurosci*. 2009;31:363-377

20. Kaur P, Jodhka PK, Underwood WA, Bowles CA, de Fiebre NC, de Fiebre CM, Singh M. Progesterone increases brain-derived neuroptrophic factor expression and protects against glutamate toxicity in a mitogen-activated protein kinase- and phosphoinositide-3 kinase-dependent manner in cerebral cortical explants. *J Neurosci Res*. 2007;85:2441-2449

21. Migliaccio A, Piccolo D, Castoria G, Di Domenico M, Bilancio A, Lombardi M, Gong W, Beato M, Auricchio F. Activation of src/p21/erk pathway by progesterone receptor via cross-talk with estrogen receptor. *EMBO*. 1998;17:2008-2018

22. Singh M. Ovarian hormones elicit phosphorylation of akt and extracellular-signal regulated kinase in explants of the cerebral cortex. *Endocrine*. 2001;14:407-415

23. Collado ML, Rodriquez-Manzo G, Cruz ML. Effect of progesterone upon adenylate cyclase activity and camp levels on brain areas. *Pharmacology biochemistry and behavior*. 1984;23:501-504

24. Hoffman GE, Moore N, Fiskum G, Murphy AZ. Ovarian steriod modulation of seizure severity and hippocampul cell death after kainic acid treatment. *Experimental Neurology*. 2003;182:124-134

25. Collaer ML, Hines M. Human behavioral sex differences: A role for gonadal hormones during early development? *Psychol Bull*. 1995;118:55-107

26. Peper JS, van den Heuvel MP, Mandl RC, Hulshoff Pol HE, van Honk J. Sex steroids and connectivity in the human brain: A review of neuroimaging studies. *Psychoneuroendocrinology*. 2011;36:1101-1113

27. Galli KK, Zimmerman RA, Jarvik GP, Wernovsky G, Kuypers MK, Clancy RR, Montenegro LM, Mahle WT, Newman MF, Saunders AM, Nicolson SC, Spray TL, Gaynor JW. Periventricular leukomalacia is common after neonatal cardiac surgery. *J Thorac Cardiovasc Surg*. 2004;127:692-704

28. Mahle WT, Tavani F, Zimmerman RA, Nicolson SC, Galli KK, Gaynor JW, Clancy RR, Montenegro LM, Spray TL, Chiavacci RM, Wernovsky G, Kurth CD. An mri study of neurological injury before and after congenital heart surgery. *Circulation*. 2002;106:I109-114

29. Miller RH. Regulation of oligodendrocyte development in the vertebrate cns. *Progress in Neurobiology*. 2002;67:451-467

30. Ghoumari AM, Baulieu EE, Schumacher M. Progesterone increases oligodendroglial cell proliferation in rat cerebellar slice cultures. *Neuroscience*. 2005;135:47-58

31. Baulieu EE, Schumacher. M. Progesterone as a neuroactive neurosteroid, with special reference to the effect of progesterone on myelination. *Human Reproduction*. 2000;15:1-13

32. Ghoumari AM, Ibanez, C., El-Etr, M., Leclerc, P.,Eychenne, B., O'Malley, B.W.,Baulieu, E.E.,Schumacher, M. Progesterone and its metabolites increase

myelin basic protein expression in organotypic slice cultures of rat cerebellum. *J Neurochem*. 2003;86:848-859

33. Kelleher MA, Palliser HK, Walker DW, Hirst JJ. Sex-dependent effect of a low neurosteroid environment and intrauterine growth restriction on foetal guinea pig brain development. *J Endocrinol*. 2011;208:301-309

34. Hirst JJ, Palliser HK, Yates DM, Yawno T, Walker DW. Neurosteroids in the fetus and neonate: Potential protective role in compromised pregnancies. *Neurochem Int*. 2008;52:602-610

35. Jodhka PK, Kaur P, Underwood W, Lydon JP, Singh M. The differences in neuroprotective efficacy of progesterone and medroxyprogesterone acetate correlate with their effects on brain-derived neurotrophic factor expression. *Endocrinology*. 2009;150:3162-3168

36. Northen AT, Norman GS, Anderson K, Moseley L, Divito M, Cotroneo M, Swain M, Bousleiman S, Johnson F, Dorman K, Milluzzi C, Tillinghast JA, Kerr M, Mallett G, Thom E, Pagliaro S, Anderson GD. Follow-up of children exposed in utero to 17 alpha-hydroxyprogesterone caproate compared with placebo. *Obstet Gynecol*. 2007;110:865-872

37. Trotter A, Maier L, Grill HJ, Kohn T, Heckmann M, Pohlandt F. Effects of postnatal estradiol and progesterone replacement in extremely preterm infants. *J Clin Endocrinol Metab*. 1999;84:4531-4535

38. Trotter A, Bokelmann B, Sorgo W, Bechinger-Kornhuber D, Heinemann H, Schmucker G, Oesterle M, Kohntop B, Brisch KH, Pohlandt F. Follow-up examination at the age of 15 months of extremely preterm infants after postnatal estradiol and progesterone replacement. *J Clin Endocrinol Metab*. 2001;86:601-603

39. Trotter AS, J., Kron, M., Pohlandt, F. Neurodevelopmental follow-up at five years corrected age of extremely low birth weight infants after postnatal replacement of 17-estradiol and progesterone. *Journal of Clinical Endocrinology and Metabolism*. 2012;97:1041-1047

40. Rode L, Langhoff-Roos J, Andersson C, Dinesen J, Hammerum MS, Mohapeloa H, Tabor A. Systematic review of progesterone for the prevention of preterm birth in singleton pregnancies. *Acta Obstet Gynecol Scand*. 2009;88:1180-1189

41. Smith R. Parturition. *N Engl J Med*. 2007;356:271-283

42. Sotiriadis A, Papatheodorou S, Makrydimas G. Perinatal outcome in women treated with progesterone for the prevention of preterm birth: A meta-analysis. *Ultrasound Obstet Gynecol*. 2012;40:257-266

43. Dodds JM, Flenady V, Cincotta R, Crowther CA. Prenatal administration of progesterone for preventing preterm birth in women considered to be at risk for preterm birth. *The Cochrane Collaboration*. 2013

44. Morris RK, Oliver EA, Malin G, Khan KS, Meads C. Effectiveness of interventions for the prevention of small-for-gestational age fetuses and perinatal mortality: A review of systematic reviews. *Acta Obstet Gynecol Scand*. 2013;92:143-151

45. Ransom CE, Murtha AP. Progesterone for preterm birth prevention. *Obstet Gynecol Clin North Am*. 2012;39:1-16, vii

46. Serra V, Perales A, Meseguer J, Parrilla JJ, Lara C, Bellver J, Grifol R, Alcover I, Sala M, Martinez-Escoriza JC, Pellicer A. Increased doses of vaginal progesterone for the prevention of preterm birth in twin pregnancies: A randomised controlled double-blind multicentre trial. *BJOG*. 2013;120:50-57

47. Norman JE, Shennan A, Bennett P, Thornton S, Robson S, Marlow N, Norrie J, Petrou S, Sebire N, Lavender T, Whyte S. Trial protocol opptimum-- does progesterone prophylaxis for the prevention of preterm labour improve outcome? *BMC Pregnancy Childbirth*. 2012;12:79

48. Norwitz ER, A.B. C. Progesterone supplementation and the prevention of preterm birth. *Reviews in obstetrics and gynecology*. 2011;4:60

49. Progesterone and preterm birth prevention: Translating clinical trials data into clinical practice. *Am J Obstet Gynecol*. 2012;206:376-386

50. Farine D, Mundle WR, Dodd J. The use of progesterone for prevention of preterm birth. *J Obstet Gynaecol Can*. 2008;30:67-71

51. Cnota JF, Gupta R, Michelfelder EC, Ittenbach RF. Congenital heart disease infant death rates decrease as gestational age advances from 34 to 40 weeks. *J Pediatr*. 2011;159:761-765

52. Costello JM, Polito A, Brown TF, McElrath TF, Graham RR, Thiagarajan EA, Bacha CA, Allen CK, Cohen JN, Laussen PC. Birth before 39 weeks' gestation is associated with worse outcomes in neonates with heart disease. *Pediatrics*. 2010;126:277-284

53. Goff DA, Luan X, Gerdes M, Bernbaum J, D'Agostino JA, Rychik J, Wernovsky G, Licht DJ, Nicolson SC, Clancy RR, Spray TL, Gaynor JW. Younger gestational age is associated with worse neurodevelopmental outcomes after cardiac surgery in infancy. *J Thorac Cardiovasc Surg*. 2012;143:535-542

54. Ballweg JA, Wernovsky G, Gaynor JW. Neurodevelopmental outcomes following congenital heart surgery. *Pediatr Cardiol*. 2006:1450-1459

55. Shillingford AJ, Glanzman MM, Ittenbach RF, Clancy RR, Gaynor JW, Wernovsky G. Inattention, hyperactivity, and school performance in a population of school-age children with complex congenital heart disease. *Pediatrics*. 2008;121:e759-767

56. Wernovsky G, Shillingford AJ, Gaynor JW. Central nervous system outcomes in children with complex congenital heart disease. *Current Opinion in Cardiology*. 2005;20:94-99

57. Marino BS, Lipkin PH, Newburger JW, Peacock G, Gerdes M, Gaynor JW, Mussatto KA, Uzark K, Goldberg CS, Johnson WH, Jr., Li J, Smith SE, Bellinger DC, Mahle WT. Neurodevelopmental outcomes in children with congenital heart disease: Evaluation and management: A scientific statement from the american heart association. *Circulation*. 2012;126:1143-1172

58. Bellinger DC, Newburger JW, Wypij D, al. e. Behaviour at eight years in children with surgically corrected transposition: The boston circulatory arrest trial. *Cardiol Young*. 2009;19:86-97

59. Bellinger D, Rappaport L, Wypij D, Wernovsky G, Newburger J. Patterns of developmental dysfunction after surgery during infancy to correct transposition of the great arteries. . *J Dev Behav Pediatr*. 1997;18:75-83

60. Bellinger DC, Wypij D, duPlessis AJ, Rappaport LA, Jonas RA, Wernovsky G, Newburger JW. Neurodevelopmental status at eight years in children with dextro-transposition of the great arteries: The boston circulatory arrest trial. *J Thorac Cardiovasc Surg*. 2003;126:1385-1396

61. Bellinger DC, Wypij D, Kuban KC, Rappaport LA, Hickey PR, Wernovsky G, Jonas RA, Newburger JW. Developmental and neurological status of children at 4 years of age after heart surgery with hypothermic circulatory arrest or low-flow cardiopulmonary bypass. *Circulation*. 1999;100:526-532

62. Bellinger DC, Wypij D, Rivkin MJ, DeMaso DR, Robertson RL, Jr., Dunbar-Masterson C, Rappaport LA, Wernovsky G, Jonas RA, Newburger JW. Adolescents with d-transposition of the great arteries corrected with the arterial switch procedure: Neuropsychological assessment and structural brain imaging. *Circulation*. 2011;124:1361-1369

63. Newburger JW, Jonas RA, Wernovsky G, Wypij D, Hickey PR, Kuban KC, Farrell DM, Holmes GL, Helmers SL, Constantinou J, et al. A comparison of the perioperative neurologic effects of hypothermic circulatory arrest versus low-flow cardiopulmonary bypass in infant heart surgery. *N Engl J Med*. 1993;329:1057-1064

64. Rivkin MJ, Watson CG, Scoppettuolo LA, Wypij D, Vajapeyam S, Bellinger DC, DeMaso DR, Robertson RL, Jr., Newburger JW. Adolescents with d-transposition of the great arteries repaired in early infancy demonstrate reduced white matter microstructure associated with clinical risk factors. *J Thorac Cardiovasc Surg*. 2013;146:543-549

65. Hirsch JC, Jacobs ML, Andropoulos D, Austin EH, Jacobs JP, Licht DJ, Pigula F, Tweddell JS, Gaynor JW. Protecting the infant brain during cardiac surgery: A systematic review. *Ann Thorac Surg*. 2012;94:1365-1373; discussion 1373

66. Licht DJ, Shera DM, Clancy RR, Wernovsky G, Montenegro LM, Nicolson SC, Zimmerman RA, Spray TL, Gaynor JW, Vossough A. Brain maturation is delayed in infants with complex congenital heart defects. *J Thorac Cardiovasc Surg*. 2009;137:529-536

67. Licht DJ, Wang J, Silvestre DW, Nicolson SC, Montenegro LM, Wernovsky G, Tabbutt S, Durning SM, Shera DM, Gaynor JW, Spray TL, Clancy RR, Zimmerman RA, Detre JA. Preoperative cerebral blood flow is diminished in neonates with severe congenital heart defects. *J Thorac Cardiovasc Surg*. 2004;128:841-849

68. Miller SP, McQuillen PS, Hamrick S, Xu D, Glidden DV, Charlton N, Karl T, Azakie A, Ferriero DM, Barkovich AJ, Vigneron DB. Abnormal brain development in newborns with congenital heart disease. *New Engl J Med*. 2007;357:1928-1938

69. Limperopoulos C, Tworetzky W, McElhinney DB, Newburger JW, Brown DW, Robertson RL, Guizard N, McGrath E, Geva J, Annese D, Dunbar-Masterson C, Trainor B, Laussen P, du Plessis AJ. Brain volume and metabolism in fetuses with congenital heart disease: Evaluation with quantitative magnetic resonance imaging and spectroscopy. *Circulation*. 2010;121:26-33

70. Andropoulos DB, Hunter JV, Nelson DP, Stayer SA, Stark AR, McKenzie ED, Heinle JS, Graves DE, Fraser CD, Jr. Brain immaturity is associated with brain injury before and after neonatal cardiac surgery with high-flow bypass and cerebral oxygenation monitoring. *J Thorac Cardiovasc Surg*. 2010;139:543-556

71. Beca J, Gunn, J.K., Coleman, L., Hope, A., Reed, P.W., Hunt, R.W., Finucane, K., Brizard, C., Dance, B., Shekerdemian, L.S. New white matter brain injury after infant heart surgery is associated with diagnostic group and the use of circulatory arrest. *Circulation*. 2013;127:971-979

72. Kaltman JR, Di H, Tian Z, Rychik J. Impact of congenital heart disease on cerebrovascular blood flow dynamics in the fetus. *Ultrasound in Obstetrics & Gynecology*. 2005;25:32-36

73. Heck S, Schindler T, Smyth J, Lui K, Meriki N, Welsh A. Evaluation of neonatal regional cerebral perfusion using power doppler and the index fractional moving blood volume. *Neonatology*. 2012;101:254-259

74. Cruz-Martinez R, Figueras F, Hernandez-Andrade E, Benavides-Serralde A, Gratacos E. Normal reference ranges of fetal regional cerebral blood perfusion as measured by fractional moving blood volume. *Ultrasound Obstet Gynecol*. 2011;37:196-201

75. Cruz-Martinez R, Figueras F, Oros D, Padilla N, Meler E, Hernandez-Andrade E, Gratacos E. Cerebral blood perfusion and neurobehavioral performance in full-term small-for-gestational-age fetuses. *Am J Obstet Gynecol*. 2009;201:474 e471-477

76. Hernandez-Andrade E, Jansson T, Figueroa-Diesel H, Rangel-Nava H, Acosta-Rojas R, Gratacos E. Evaluation of fetal regional cerebral blood perfusion using power doppler ultrasound and the estimation of fractional moving blood volume. *Ultrasound Obstet Gynecol*. 2007;29:556-561

77. Wallenstein MB, Harper LM, Odibo AO, Roehl KA, Longman RE, Macones GA, Cahill AG. Fetal congenital heart disease and intrauterine growth restriction: A retrospective cohort study. *J Matern Fetal Neonatal Med*. 2012;25:662-665

78. Redline RW. Cerebral palsy in term infants: A clinicopathologic analysis of 158 medicolegal case reviews. *Pediatr Dev Pathol*. 2008;11:456-464

79. Chang T, du Plessis A. Neurodiagnostic techniques in neonatal critical care. *Curr Neurol Neurosci Rep*. 2012;12:145-152

80. Elbers J, Viero S, MacGregor D, DeVeber G, Moore AM. Placental pathology in neonatal stroke. *Pediatrics*. 2011;127:e722-729

81. Ashton DM. Elective delivery at less than 39 weeks. *Curr Opin Obstet Gynecol*. 2010;22:506-510

82. Mayhew TM. Taking tissue samples from the placenta: An illustration of principles and strategies. *Placenta*. 2008;29:1-14

83. Mayhew TM. A stereological perspective on placental morphology in normal and complicated pregnancies. *J Anat*. 2009;215:77-90

84. Sadovsky Y, Wyatt SM, Collins L, Elchalal U, Kraus FT, Nelson DM. The use of needle biopsy for assessment of placental gene expression. *American Journal of Obstetrics and Gynecology*. 2006;194:1137

85. Rubin JM, Bude RO, Fowlkes JB, Spratt RS, Carson PL, Adler RS. Normalizing fractional moving blood volume estimates with power doppler us: Defining a stable intravascular point with the cumulative power distribution function. *Radiology*. 1997;205:757-765

86. Hernandez-Andrade E, Jansson T, Ley D, Bellander M, Persson M, Lingman G, Marsal K. Validation of fractional moving blood volume measurement with power doppler ultrasound in an experimental sheep model. *Ultrasound Obstet Gynecol*. 2004;23:363-368

87. Carter AS, Briggs-Gowan MJ. *Infant-toddler social and emotional assessment.* San Antonio: PsychCorp; 2006.

88. Xu W, Hedeker D. A random-effects mixture model for classifying treatment response in longitudinal clinical trials. *J Biopharm Stat*. 2001;11:253-273

89. Virzi L, Pemberton V, Ohye RG, Tabbutt S, Lu M, Atz TC, Barnard T, Dunbar-Masterson C, Ghanayem NS, Jacobs JP, Lambert LM, Lewis A, Pike N, Pizarro C, Radojewski E, Teitel D, Xu M, Pearson GD. Reporting adverse events in a surgical trial for complex congenital heart disease: The pediatric heart network experience. *J Thorac Cardiovasc Surg*. 2011;142:531-537

90. Yanushpolsky E, Hurwitz S, L G, Racowsky C, Hornstein M. Crinone vaginal gel is equally effective and better tolerated then intramuscular progesterone for luteal phase support in in vitro fertilization-embryo transfer cycles: A prospective randomized study. *Fertility Sterility*. 2010;94:2596

91. Shellock F. *Reference manual for magnetic resonance safety, implants and devices*. Los Angeles: Biomedical research publishing group; 2007.

92. Myers C, Duncan KR, Gowland PA, Johnson IR, Baker PN. Failure to detect intrauterine growth restriction following in utero exposure to mri. *Br J Radiol*. 1998;71:549-551

93. Radiology ACoRaSfP. Practice guideline for the safe and optimal performance of fetal magnetic resonance imaging (mri), resolution (13). *American Journal of Radiology Journal*. 2010

94. Baker PN, Johnson IR, Harvey PR, Gowland PA, Mansfield P. A three-year follow-up of children imaged in utero with echo-planar magnetic resonance. *Am J Obstet Gynecol*. 1994;170:32-33

95. Reeves MJ, Brandreth M, Whitby EH, Hart AR, Paley MN, Griffiths PD, Stevens JC. Neonatal cochlear function: Measurement after exposure to acoustic noise during in utero mr imaging. *Radiology*. 2010;257:802-809

96. Haacke EM, Brown RW, Thompson MR, Venkatesan R. *Magnetic resonance imaging*. John Wiley & Sons, Inc.; 1999.

97. Licht DJ, Wang J, Silvestre DW, Nicolson SC, Montenegro LM, Wernovsky G, Tabbutt S, Durning SM, Shera DM, Gaynor JW, Spray TL, Clancy RR, Zimmerman RA, Detre JA. Preoperative cerebral blood flow is diminished in neonates with severe congenital heart defects. *Journal of Thoracic & Cardiovascular Surgery*. 2004;128:841-849

APPENDIX

Appendix 1 Data Elements from Medical Record Review from Section 5

Section 5.1.1 Pre-Natal Medical Record Review

Date of Birth

Race& Ethnicity

Marital Status

Occupation

Demographic: address, phone number(s), email address(s), alternate contacts

Physical Examination

Vital Signs: Blood pressure/ pulse (at each visit)

Growth Parameters

Concomitant medications

Allergies

Last Menstrual Period

Gestational Age

Estimated Date of Confinement

IVF fertilization date or embryo transfer

Pre-pregnancy weight

Body Mass Index

Weights and weight gain

OB History: Gravida, Para, Term, Preterm, TAB, SAB, Ect, Mult Living

Antepartum, intrapartum, postpartum complications

History of STD's

Abnormal Pap Smears

Genitourinary Abnormalities

Family medical history

Medical and surgical history

Psychiatric history

Smoking

Drugs

Alcohol

Exposure to Teratogens

Stress

Mental Status

Social Support

History of past or current physical abuse

Pregnancy Readiness Planned/Unplanned

Housing

Finances

Clinically indicated laboratory evaluations obtained by referring physician or CHOP's CFDT (Section 5.1.5)

Section 5.1.2 Post-Natal Medical Record Review

BIRTH

- Date of Birth
- Gender
- Location of Birth
- Gestational Age
- Mode of Delivery
- Apgar Scores
- Weight at Birth
- Length at Birth
- Head Circumference at Birth
- Adverse Events at delivery [Seizures, arrest, intubation, PGE]

INPATIENT ADMISSION(s)

Date of Admission
Medical Record Number
Diagnosis: CHD
Genetic Anomaly
Extra-Cardiac Anomaly
Data from clinical genetic consult(s)
Daily Weight
Daily Feeding Status
Daily Lowest Diastolic Blood Pressure
Daily Arterial Blood Gas [lowest PO2, lowest PCO2, lowest pH, and highest pH]
Type and duration of mechanical ventilation
Type and duration of intrathoracic catheters
Type and duration of intracardiac lines
Daily Events [NeuroCardiac Research Program database daily event code list]
Date of Surgery
Weight at Surgery
Surgical Procedure
Surgeon
Number of CPB Runs
CPB Time(s)
DHCA Time(s)
Cross Clamp Time(s)
Cooling Time(s)
Rewarming Time(s)
MUF

NP Temperature

Baseline Hematocrit

Hematocrit on CPB

Hematocrit following modified ultrafiltration

Hematocrit on admission to CICU

Anesthesiologist

Arterial Blood Gas 5 minutes after Commencement of Bypass

Blood Product Usage

Medication Usage

Cardiac Arrest Events

Reoperations

ECMO/VAD

Delayed Sternal Closure

Death

Date of Death

Date of Discharge

Cardiologist

OUTPATIENT

Events including procedures between discharge from neonatal surgery and 18month follow up
