# Supplementary material for: Progesterone for Neurodevelopment in Fetuses With Congenital Heart Defects: A Randomized Clinical Trial
Source: JAMA Netw Open. 2024 May 28;7(5):e2412291. doi: 10.1001/jamanetworkopen.2024.12291 (PMC11134212; doi:10.1001/jamanetworkopen.2024.12291)
Supplement: Supplement 3. — Data Sharing Statement [file jamanetwopen-e2412291-s003.pdf]

Data Sharing Statement

Gaynor. Effect of Progesterone on Neurodevelopment in Fetuses With Congenital Heart Defects. *JAMA Netw Open*. Published May 28, 2024.
doi:10.1001/jamanetworkopen.2024.12291

Data
Data available: No

Additional Information
Explanation for why data not available: We will consider all requests and share upon reasonable requests. Based on Institutional regulations, sharing de-identified data will require a DUA.
